# A symmoriiform from the Late Devonian of Morocco demonstrates a derived jaw function in ancient chondrichthyans

**DOI:** 10.1038/s42003-020-01394-2

**Published:** 2020-11-17

**Authors:** Linda Frey, Michael I. Coates, Kristen Tietjen, Martin Rücklin, Christian Klug

**Affiliations:** 1 Paläontologisches Institut und Museum, University of Zurich, Karl-Schmid-Strasse 4, CH-8006 Zürich, Switzerland; 2 Department of Organismal Biology and Anatomy, University of Chicago, 1027 E. 57th St., Chicago, IL 60637 USA; 3 Biodiversity Institute, University of Kansas, 1345 Jayhawk Blvd, Lawrence, KS 66045 USA; 4 Naturalis Biodiversity Center, Vertebrate Evolution Development and Ecology, Postbus 9517, 2300 RA Leiden, The Netherlands

**Keywords:** Biomechanics, Palaeontology, Ichthyology

## Abstract

The Palaeozoic record of chondrichthyans (sharks, rays, chimaeras, extinct relatives) and thus our knowledge of their anatomy and functional morphology is poor because of their predominantly cartilaginous skeletons. Here, we report a previously undescribed symmoriiform shark, *Ferromirum oukherbouchi*, from the Late Devonian of the Anti-Atlas. Computed tomography scanning reveals the undeformed shape of the jaws and hyoid arch, which are of a kind often used to represent primitive conditions for jawed vertebrates. Of critical importance, these closely fitting cartilages preclude the repeatedly hypothesized presence of a complete gill between mandibular and hyoid arches. We show that the jaw articulation is specialized and drives mandibular rotation outward when the mouth opens, and inward upon closure. The resultant eversion and inversion of the lower dentition presents a greater number of teeth to prey through the bite-cycle. This suggests an increased functional and ecomorphological disparity among chondrichthyans preceding and surviving the end-Devonian extinctions.

## Abstract (web summary)

Frey and colleagues describe a new fossil shark from the Late Devonian of Morocco in which the specialized jaw articulation is unusually well preserved. Biomechanical modelling reveals that the mandible rolled throughout its movement arc, engaging more of its tooth battery in a clutching bite action.

## Introduction

The Symmoriiformes is a widely distributed group of early chondrichthyans ranging from the Late Devonian through to the early Permian, and perhaps extending as far as the Cretaceous^1–4^. Like the vast majority of early chondrichthyans, symmoriiforms have mostly been understood from two-dimensional skeletal and, more rarely, soft tissue remains, supplemented with insights from hard tissue histology^5–10^. Symmoriiforms, occasionally including the classic Devonian genus *Cladoselache*, have often been used to exemplify early chondrichthyan conditions, and, from entrenched views of sharks as intrinsically primitive, generalised gnathostome conditions. Consistent with this treatment, both traditional and some of the more current phylogenetic hypotheses have placed this group (whether grade or clade) on the chondrichthyan stem^11,12^. However, an alternative series of analyses resolve symmoriiforms as stem holocephalans^2,3,10,13,14^. Notably, the most recent of these hypotheses employ the abundance of new data from X-ray tomography, especially concerning the neurocranium^3,11,15,16^.

Here, we describe a symmoriiform chondrichthyan from the Devonian of Morocco and investigate the morphology and biomechanics of the superbly preserved jaws and hyoid arch. These jaws display the classic ‘cleaver’ shape^17^ palate seen in a vast array of early crown-gnathostomes, and especially among chondrichthyans^2^. However, in this unique specimen, the areas of attachment to the neurocranium and the jaw joint are undistorted, and likewise the slender cartilages of the hyoid arch. Thus, the aims of this study are threefold: first, to describe morphology and characterise the new taxon; second, to add these data to a revised taxon and character matrix and test the robustness of recent phylogenetic hypotheses; third, to investigate the motion of the jaws. Importantly, the preservation of the hyoid and mandibular arches is such that a physical reconstruction of the feeding apparatus is possible, allowing an investigation of jaw movements (kinematics) in three dimensions^18^, and thus, a more detailed comparison with the feeding mechanics of living chondrichthyans^19^. By these means, it may be possible to gain a better appreciation of symmoriid chondrichthyans as early specialists or (simply) generalist predators, and add to an emerging picture of functional disparity and ecomorphological partitioning among these early members of the modern vertebrate biota^18,20^.

## Results

### Systematic palaeontology

Chondrichthyes Huxley, 1880^21^

Total-group Holocephali Bonaparte, 1832^22^

Symmoriiformes Zangerl, 1981^1^

*Ferromirum* gen. nov.

**Etymology.** Derived from *ferrum* (lat.—iron) and *mirus* (lat.—miraculous). *Ferrum* refers to the preservation of the holotype in a reddish ferruginous nodule, which is characteristic for fossils from the Thylacocephalan Layer of the Maïder. *Mirus* refers to our initial misinterpretation of the gill remains of the holotype as crustacean appendages, before preparation and the miracle-like revelation that the specimen was, in fact, a chondrichthyan.

**Type species.** *F. oukherbouchi* sp. nov.

**Diagnosis.** A small symmoriid with slender body; head with short triangular rostrum and subterminal gape; supraorbital shelf with concave lateral margin; orbits large (ca. 30% of neurocranium length) with sclerotic ring; narrow interorbital space; narrow suborbital shelf (ca. 7% of neurocranium width); cleaver-shaped palatoquadrate with anterolaterally directed articulation with postorbital process; scalloped margins of gape suggest around nine upper and lower tooth families; teeth small, cladodont with a prominent medium cusp with small lateral cusps and minute intermediate cusps; slender ceratohyal with posteroventral lateral flange engaging with Meckel’s cartilage; paired hypohyals anteriorly directed; no basihyal; distinguished from all other symmoriids by presence of pectoral-level, slender dorsal fin spine, smooth with posteriorly curved apex.

*Ferromirum oukherbouchi* sp. nov.

**Etymology.** The species name *oukherbouchi* honours the finder of the specimen Said Oukherbouch (Tafraoute).

**Holotype.** PIMUZ A/I 4806

**Material.** Holotype alone.

**Locality.** Madene El Mrakib, Maïder Basin, southeastern Anti-Atlas, Morocco.

**Formation and age**. Ibâouane Formation (middle Famennian, Upper Devonian), Lahfira Member, Thylacocephalan Layer (formerly described as Phyllocarid Layer^14,23,24^), *Planitornoceras euryomphalum* to *Afrolobites mrakibensis* Zone.

**Diagnosis.** As for genus.

**Fig. 1:**
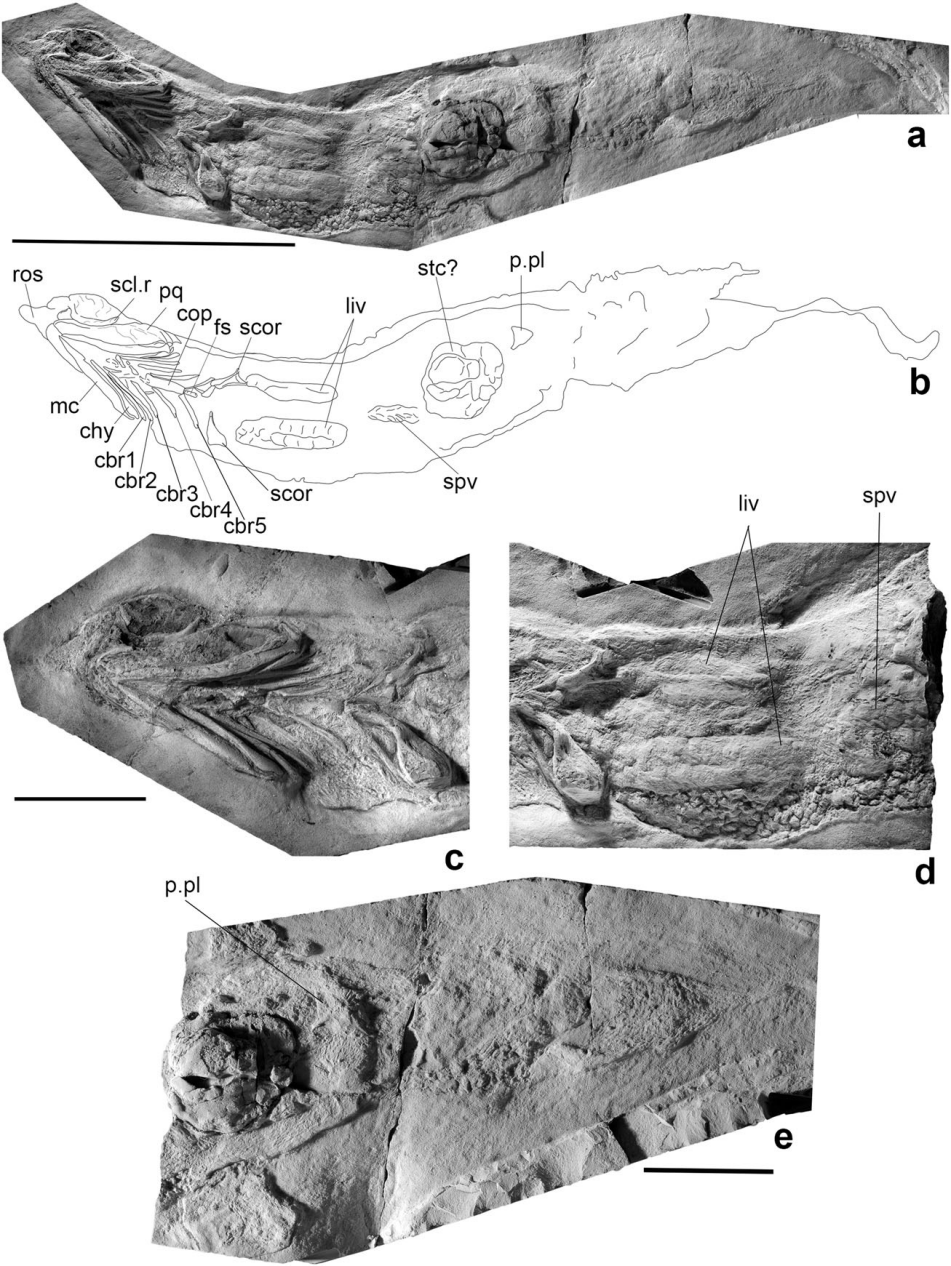
Photos of the holotype of *Ferromirum oukherbouchi* gen. et sp. nov. PIMUZ A/I 4806, early/ middle Famennian, Madene el Mrakib. **a** Photo and **b** line drawing of the specimen. **c** Head region including parts of the rostrum, sclerotic ring, mandibular arch, hyoid arch, branchial skeleton and shoulder girdle in ventral view. **d** Soft tissue remains, including liver and spiral valves. **e** Pelvic and caudal region. Scale bars, 100 mm (**a**, **b**), and 30 mm (**c**–**e**). chy, ceratohyal; cop, copula; cbr, ceratobranchials; fs, fin spine; liv, liver; mc, Meckel’s cartilage; p.pl, pelvic plate; pq, palatoquadrate; ros, rostrum; scl.r, sclerotic ring; scor, scapulocoracoid; stc?, stomach content; spv, spiral valves.

**Description.** The estimated body length of *Ferromirum oukherbouchi* gen. et sp. nov. is 330 mm. The specimen was prepared and exposed from its ventral side, revealing parts of the left orbit, mandibular, hyoid and branchial arches, pectoral and pelvic girdles (Fig. 1a–e and Supplementary Fig. 1). Substantial replacements of soft tissues are present throughout the body. Anteriorly, these reveal that the snout forms a short, pointed rostrum, resembling examples preserved in *Falcatus* and *Damocles*^7,8^. As in these genera, there is no evidence of skeletal support for the rostral apex, which likely housed arrays of electro- and mechanoreceptive organs (possible remains are visible as minute circular pits on its venter; arrows in Supplementary Fig. 2i). The trunk region includes two elongate lobes of the liver (Fig. 1d), extending for at least 50% of the visceral cavity length (it is unclear if the complete length of the liver lobes is visible; in modern elasmobranchs, the liver can be proportionally longer). Part of the digestive tract is evident as a spiral valve, exposed in the midline between the caudalmost extremities of both liver lobes (Fig. 1a, b). Directly anterior to the pelvic plates, a large bolus of material might represent a mass of pre-rectal gut contents (Fig. 1a, b, e).

**Fig. 2:**
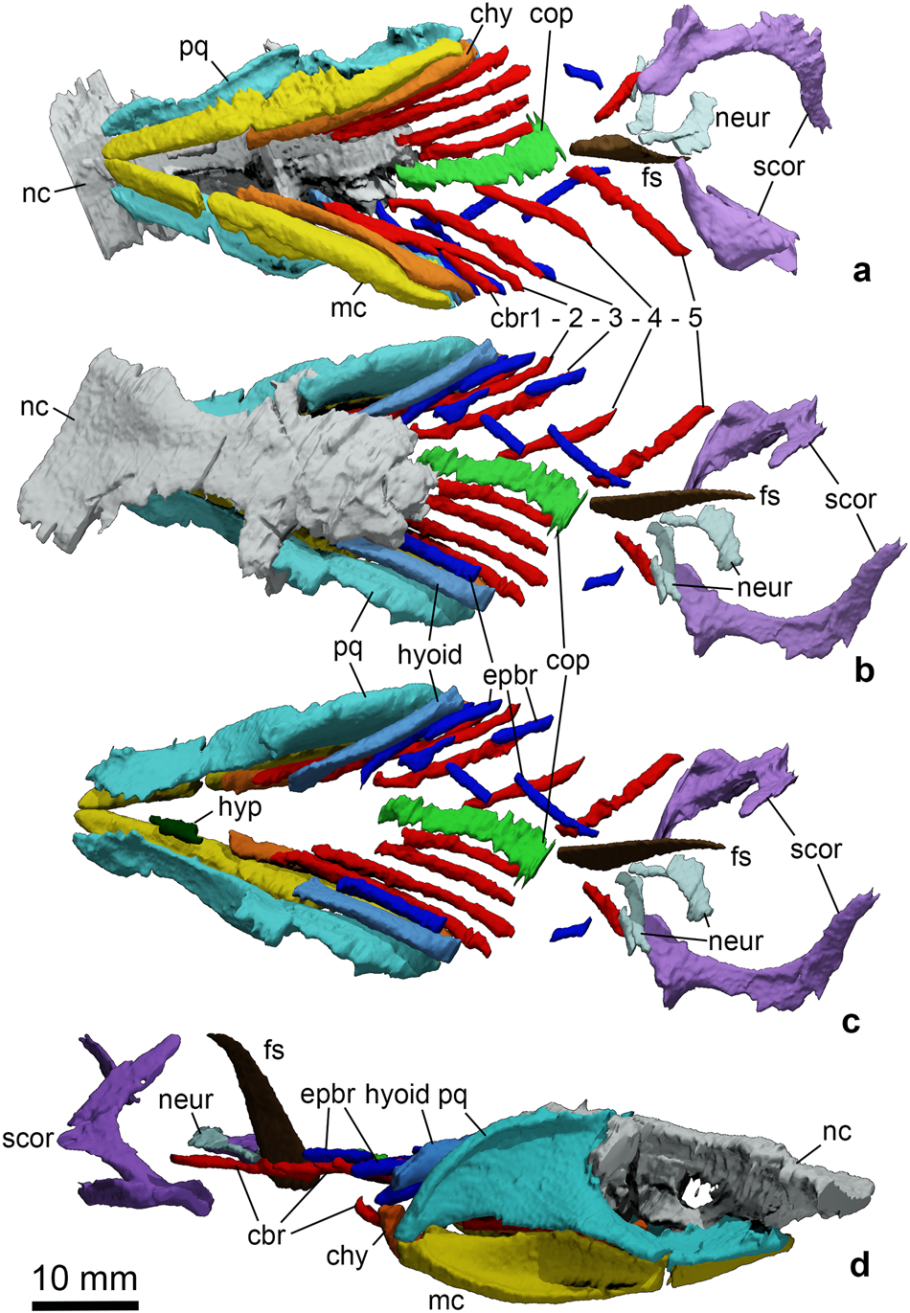
Virtual rendering of the holotype of *Ferromirum oukherbouchi* gen. et sp. nov. PIMUZ A/I 4806, based on CT-data. The virtual rendering shows the neurocranium, visceral arches, pectoral girdle and dorsal fin spine. **a** Ventral and **b** dorsal view with and **c** without braincase. **d** lateral view. Colour coding: grey, neurocranium (nc); turquoise, palatoquadrate (pq); yellow, Meckel’s cartilage (mc); dark green, hypohyal (hyp); light blue, hyoid (hyoid); orange, ceratohyal (chy); blue, epibranchials (epbr); red, ceratobranchials (cpbr); green, copula (cop); brown, fin spine (fs); purple, pectoral girdle (scor); light turquoise,? neural arches (neur).

**Fig. 3:**
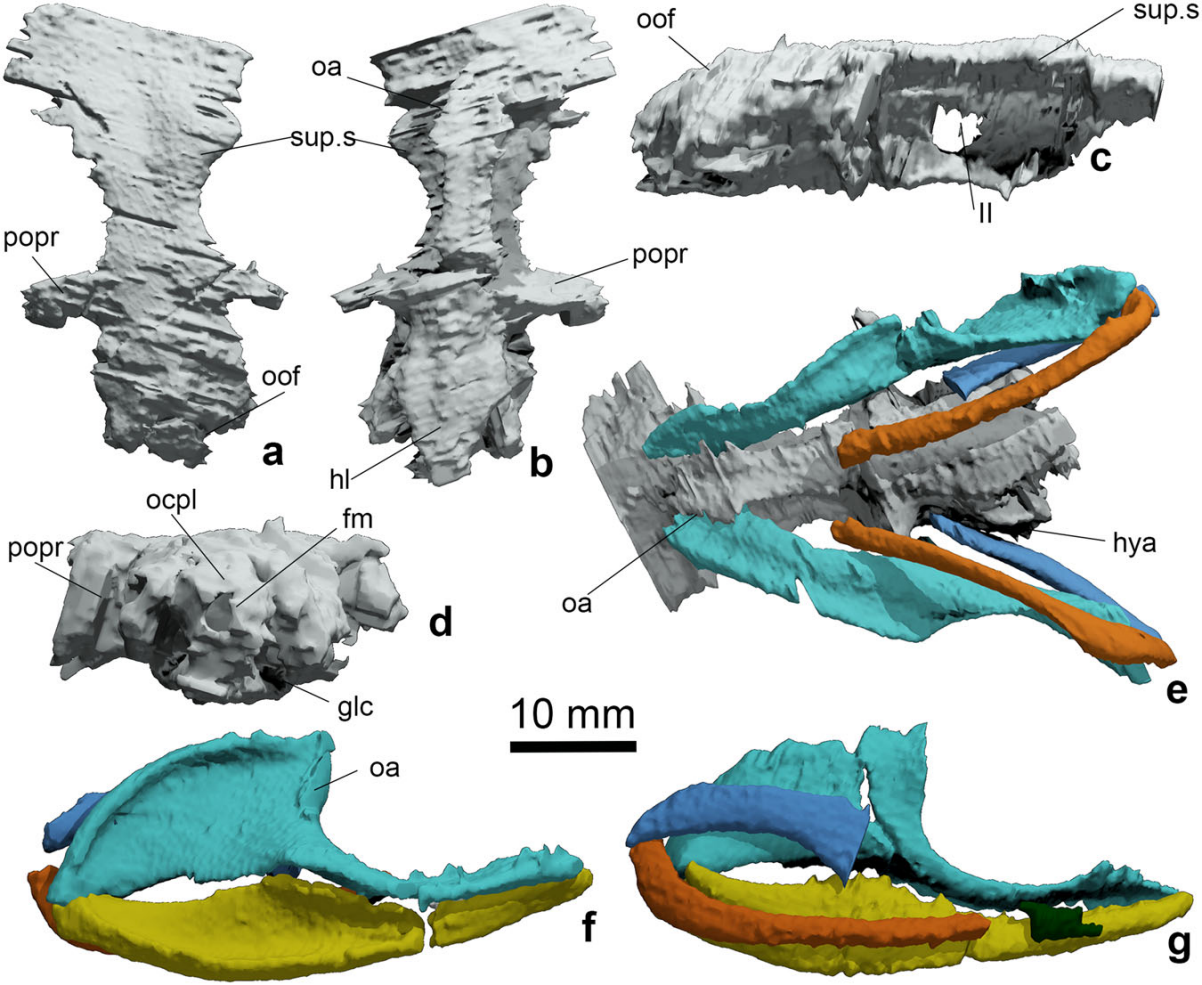
Neurocranium and jaws of *Ferromirum oukherbouchi* gen. et sp. nov., PIMUZ A/I 4806. Neurocranium in **a** dorsal, **b** ventral, **c** lateral, and **d** posterior, occipital views. **e** Articulation between braincase, palatoquadrate and hyoid arch in ventral view. **f**, **g** Arrangement of mandibular and hyoid arches in lateral and medial views respectively. Colour coding: grey, braincase; turquoise, palatoquadrate; yellow, Meckel’s cartilage; dark green, hypohyal; light blue, hyoid; orange, ceratohyal. Note that the rostral roof includes an excess of poorly resolved cartilage or matrix left in place in the computer renderings. fm, foramen magnum; hl, hypotic lamina; hya, hyomandibular articulation; glc, glossopharyngeal canal; oa, orbital articulation; ocpl, occipital plate; oof, otico-occipital fissure; popr, postorbital process; sup.s, supraorbital shelf; II, optic nerve.

The computed tomograms reveal details of the braincase, jaws, hyoid arch, gill skeleton, pectoral girdle and dorsal fin spine (Figs. 2, 3 and Supplementary Fig. 2i). However, the radiographic contrast between calcified cartilage and the surrounding matrix is often poor. The general shape of the neurocranium resembles that of *Ozarcus*^15,16^ and *Dwykaselachus*^3^, but the *Ferromirum* gen. nov. neurocranium has suffered post mortem compaction, possibly losing around 25% of its dorsoventral height (Figs. 2d, 3c, d). The orbit is large: the maximum span equals the rostrocaudal lengths of otic and occipital regions combined^3^. Remains of a slender sclerotic ring are exposed (Fig. 1a–c), but more detailed morphological information is not preserved. Sclerotic rings are known in *Cladoselache*^25^, *Denaea*^6^, *Falcatus*^7^ and *Damocles*^8^. A large opening for the optic nerve II perforates the mid-ventral part of the interorbital wall (Fig. 3b), and the slightly expanded anterior margin of the narrow suborbital shelf (Fig. 3b) signals presence of an articulation surface for the palatine ramus of the palatoquadrate.

The postorbital process and arcade (Fig. 3a–d) does not appear to have projected as far laterally and ventrally as those of *Dwykaselachus*, *Ozarcus* and *Akmonistion*. Rather, the process in *Ferromirum*, although likely incomplete, appears shorter, perhaps slightly more robust, proximally, and resembles that of *Gutturensis*^26^. Similarly, the *Ferromirum* supraorbital shelf is narrow with a concave lateral margin, a feature also shared by *Ozarcus* as well as *Gutturensis*. Little detail of the otic region is recognizable. The condition of the endolymphatic duct(s) is unclear, but there is no trace of a parietal fossa (Fig. 3a). Traces of the otico-occipital fissure are preserved, revealing that the dorsal portion of the occipital unit is wedged between the otic capsules. The otic wall is too poorly preserved to reveal the presence of a periotic process. The ventral surface of the neurocranium (Fig. 3b) includes the characteristic narrow waist of symmoriids^3,16^. The otic region and the glossopharyngeal canals are floored by a hypotic lamina (Fig. 3b). The openings of any canals or grooves for the dorsal aortae, expected to be present in the basicranium, are not visible. However, the occipital plate (Fig. 3d) retains a discernible foramen magnum.

Unlike the braincase, the three-dimensional form of the mandibular arch (Fig. 2a–d, 3e–f and Supplementary Fig. 2, 3) is outstandingly well preserved. The cleaver-shaped^17^ palatoquadrate has the high otic process and low palatine process^1,27^ common to many early sharks. Otic and palatine portions each account for around half of the total length. A narrow, semi-elliptical surface on the leading edge of the otic process (Fig. 3f) articulates with the postorbital process and arcade of the neurocranium. The external surface of the quadrate and otic portion of the palatoquadrate is strongly concave, forming a broad and deep attachment space for the adductor (quadratomandibularis) muscles. The medial surface is correspondingly convex, but with a gently rounded, oblique ridge marking the ventral boundary of an area that might have been occupied by the spiracular pouch (see Brazeau and Ahlberg^28^ for a comparable condition in early sarcopterygians). The otic process rim is prominent throughout all of the posterior and most of the dorsal boundary, thinning-out only at the anterodorsal extremity, just below a ridge and groove that likely supported a mandibular branch of nerve VII (Fig. 3). In lateral aspect (Fig. 3f, g), the sigmoid ventral margin of the palatoquadrate is concave downward in the quadrate region and convex downward in the palatine (cf. *Orthacanthus*^29^). In dorsal view the palatine process is mediolaterally broad, forming a substantial portion of the orbit floor (Fig. 2, Supplementary Fig. 3). The ventral surface bears a shallow dental trough (Fig. 3e and Supplementary Fig. 2c), divided into about nine concavities for generative tooth sets. Anteriorly, a slight additional medial expansion of palatine process bears a ridge and groove articulation with the suborbital process of the neurocranium. The primary articulation with Meckel’s cartilage, the quadrate condyle, is at the posterolateral extremity of the palatoquadrate (Fig. 3e and Supplementary Fig. 3). The secondary, medial articulation, the glenoid recess for the mandibular knob or process of Meckel’s cartilage, is offset both dorsally and anteriorly (Supplementary Fig. 3). The axis connecting these two surfaces subtends an angle of about 45 degrees relative to the long axis of Meckel’s cartilage, and slopes dorsomedially to ventrolaterally at an angle of 45 degrees relative to a horizontal plane connecting left and right quadrates (Fig. 3e).

**Fig. 4:**
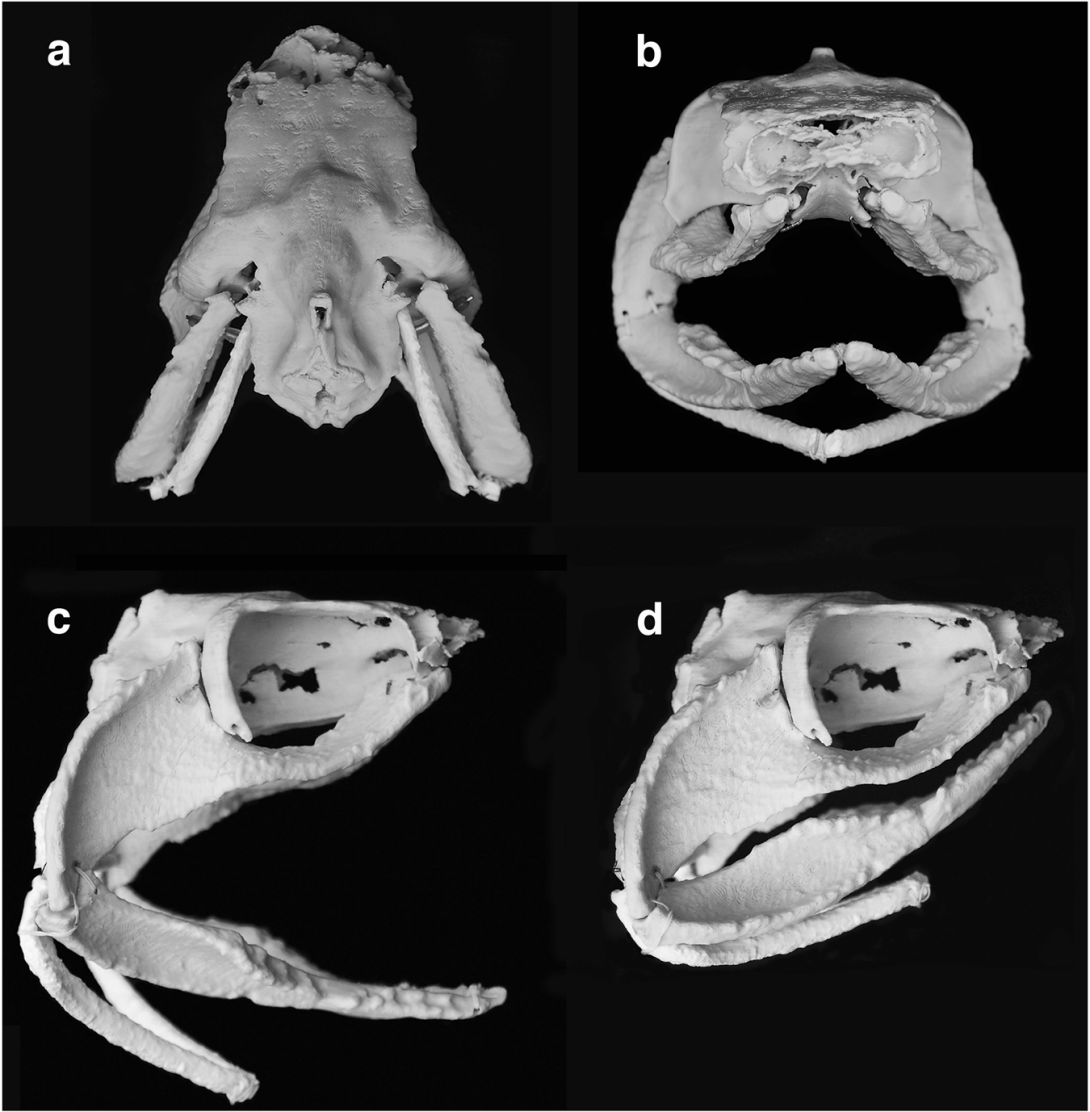
Composite model of *Ferromirum* gen. et sp. nov. jaws and hyoid arch and *Dwykaselachus* neurocranium. Composite model in **a** dorsal, **b** anterior, **c** lateral view with mandible and ceratohyal depressed, and **d** with mandible and ceratohyal raised.

The lateral, external surface of Meckel’s cartilage (Fig. 3f) is deeply concave for the posterior two thirds of its length, providing a space for adductor muscle insertion. Dorsally, the laterally expanded margin (anterior to the adductor recess) forms a platform for the dentition. The platform is narrow anteriorly but broadens posteriorly: the breadth is considerable (Supplementary Fig. 3), unlike narrower dental platforms present in taxa such as xenacanths^30^. Like the palatoquadrate, the dental trough is divided into about nine shallow concavities (Fig. 4c, e, f). A slight posterior rise of the dental platform resembles a coronoid process, but this is simply the posterior limit of the gently concave dental platform, matching the convexity of the corresponding palatine process. The articular region of the lower jaw is situated on the dorsal margin at the posterior extremity (Fig. 4e, f and Supplementary Fig. 1). The chief articular facet is a posterolateral concavity, the articular cotylus, which receives the articular process of the palatoquadrate. Anterior to the articular cotylus, the mesial margin is produced into a strong, dorsally directed mandibular knob (Fig. 3g and Supplementary Fig. 3), and this is received by the articular cotylus of the palatoquadrate. As for the upper jaw, the axis (of rotation) of this hinge lies at an angle of about 45° relative to the long axis of the lower jaw. The medial surface of the mandible is generally convex, except for the posteroventral margin, which forms a smooth concavity. In dorsal view, the long axis of Meckel’s cartilage is remarkably straight, and shows none of the characteristic curvature evident in the lower jaws of *Tristychius*^19^, *Gogoselachus*^31^, or xenacanths^29,30^. The anterior terminus of the mandible is slender, rounded and shallow, signalling the absence of any substantial, stable symphysial connection: the two halves of the lower jaw meet more or less point-to-point.

The dentition is exposed but damaged (Supplementary Fig. 4). It consists of numerous, small, symmetrical, cladodont teeth, the largest observable bases of which are barely 2 mm across. Each tooth bears a prominent median cusp flanked by diminutive slender lateral cusps; broken sections through some of the larger tooth bases suggest the presence of intermediate cusps.

Like *Ozarcus*^16^, the hyoid arch includes paired hyomandibulae, ceratohyals, and hypohyals (Fig. 2a–c and 3e, g). Each hyomandibula is gently curved, anteriorly expanded and laterally compressed. The shape matches that of the Tennessee cladoselachian^32,33^ rather than the more linear outline of the *Ozarcus* hyomandibula, and extends forward to just behind the orbit. However, unlike *Ozarcus*, the hyomandibula meets the ceratohyal directly posterior and medial to the articulation of the mandibular arch.

The ceratohyal is slender and elongate, with a dorsally curved posterior process resembling a walking stick handle (Fig. 3g and Supplementary Fig. 5). This ‘handle’ fits snugly within a shallow recess between the articular surfaces at the posterior of Meckel’s cartilage, from which it ascends to meet the hyomandibula. The ceratohyal-hyomandibula articulation aligns, dorsoventrally, with the apex of the mandibular knob of Meckel’s cartilage: i.e., level with the secondary articulation of the mandibular arch. There is a deep fossa in the posterior part of the external surface of the ceratohyal, at the point of maximum dorsal curvature (Supplementary Fig. 5). The ventrolateral margin of the fossa is extended laterally to form a gently convex flange or process that fits neatly within a matching recess in the ventromedial surface of Meckel’s cartilage (Figs. 1c, 2a). The hypohyal is simple, short, cylindrical and directed anteriorly. There is no trace of a basihyal, once again resembling conditions in *Ozarcus* 16, although we note that such absence might be a taphonomic artefact.

Five gill arches are preserved, including an apparently complete set of ceratobranchials from left and right sides (Fig. 2a–c). Epibranchials, too, include up to five members. All paired cartilages of the gill skeleton are simple rods. There are no remains of hypobranchials or pharyngobranchials. The basibranchial series is represented by a broad posterior copula^34^, somewhat like that of *Gutturensis*^26^.

The scapulocoracoid (Fig. 2 and Supplementary Fig. 6) resembles those of other symmoriiform chondrichthyans^1,10^. The flat scapula blade has a well-developed anterior process at the dorsal apex, but the posterolateral process, although broken, appears rounded. The ventral part of the scapula is mediolaterally broad as it blends into the roof of the articular surface for the pectoral fin. In ventral view, the base of the scapulacoracoid appears triangular and its posterior portion shows a concavity for the articulation with the proximal radials of the pectoral fin. The coracoid region is convex anteriorly and concave posteriorly. A procoracoid has not been detected, although present in other symmoriids^5,10^.

The pelvic girdle is known only from a small, simple triangular plate visible in the posterior of a pyrite concretion, which is located near the middle of the body (Fig. 1b,e). In other symmoriiform chondrichthyans such as *Akmonistion*, *Cobelodus*, *Denaea*, and *Symmorium*, the pelvic plate varies from subtriangular to oval^1,10^.

A dorsal fin spine is preserved at the level of the pectoral girdle (Fig. 2 and Supplementary Fig. 6f). The fin spine resembles those of cladoselachians^1,34^ in having a strong, caudally recurved dorsal apex and smooth surface bearing no ridges or tubercles. However, the overall shape and proportions of the spine are considerably narrower and longer than cladoselachian examples in lateral view. Dorsal fin conditions are unknown.

## Discussion

### Phylogenetic significance

**Fig. 5:**
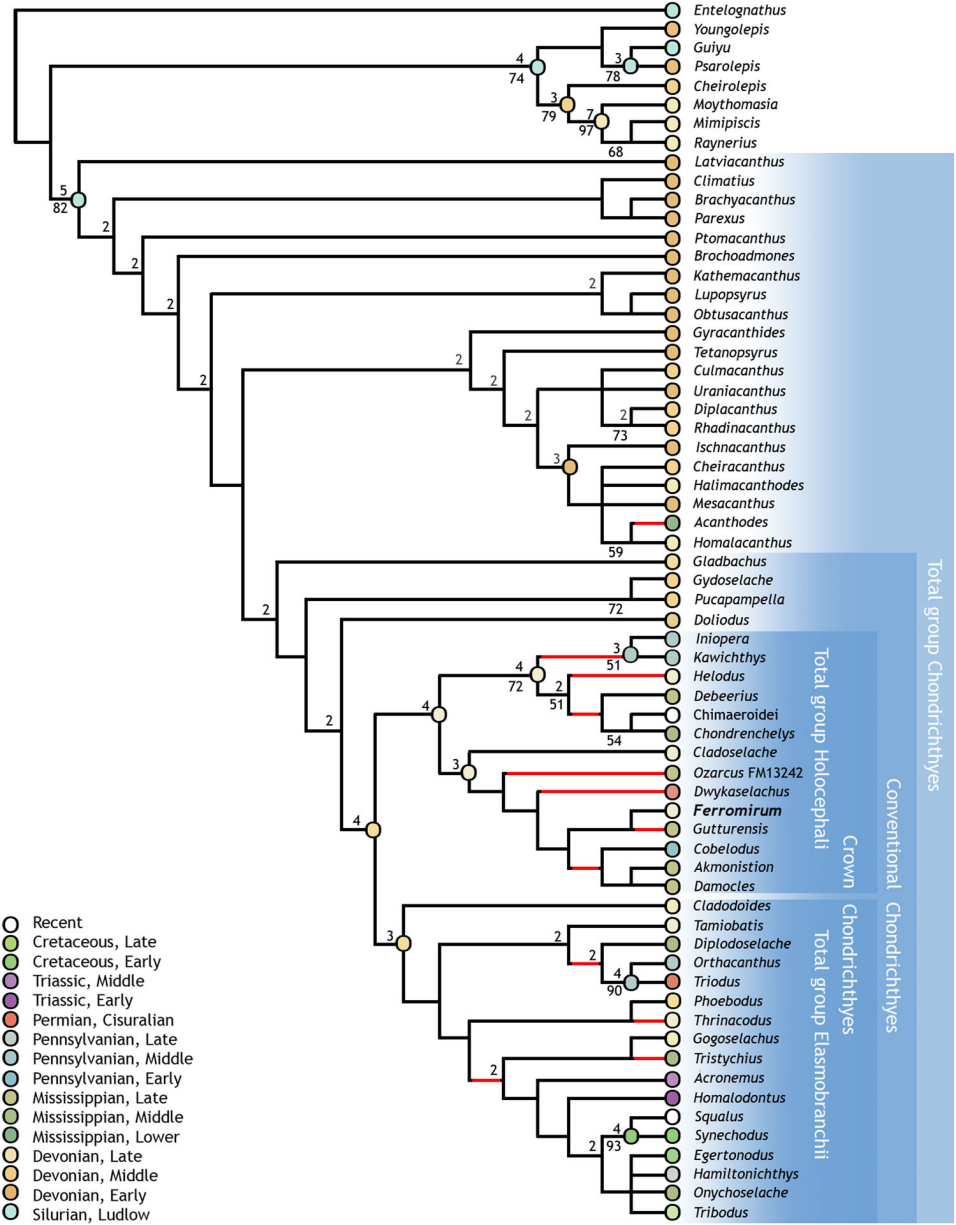
Chondrichthyan phylogeny including *Ferromirum* gen. nov. Strict consensus of 90 trees showing phylogenetic affiliation of *Ferromirum* gen. nov. among early chondrichthyans. Bremer decay values above nodes; bootstrap support scores below. Circle colours (nodes and termini) denote earliest occurrence of taxon; red branches denote clade crossing Devonian-Carboniferous boundary.

Results of phylogenetic analysis place the symmoriiform sharks, including *Ferromirum*, as a clade branching from the holocephalan stem, consistent with recent and related results^3,13,14,35^ (Fig. 5). Changes are mostly confined to the chondrichthyan stem branching pattern. Notably, *Gladbachus* no longer branches from close to the base of the total group^13^, but is instead sister group to *Pucapampella* plus *Gydoselache*, *Doliodus*, and crown chondrichthyans, corroborating results found by Dearden et al^35^. The structure of the chondrichthyan crown is reasonably robust, and signals from data partitions are mostly consistent with the overall result. Exclusion tests limiting the characters to neurocranial conditions (characters 1–4; 100–180) recover the branching structure of the chondrichthyan crown obtained from the complete data set (Adams consensus). Similarly, symmoriiforms emerge as stem-holocephalans in searches excluding neurocranial characters. However, in these trees, *Squalus* is the immediate sister of the holocephalan total-group, and putative stem-elasmobranchs (all of those genera identified as such in analyses of the complete data set: Fig. 4) are excluded from the chondrichthyan crown. Nevertheless, these putative stem-elasmobranchs persist in forming a monophyletic clade that branches from the chondrichthyan stem apex, and this result reoccurs in trees obtained from characters restricted to scales (characters 5–48), teeth (characters 65–86), and fin spines (characters 215–230).

Relationships within the symmoriiforms are unstable, and all resolution is lost in a consensus of tree lengths of only one extra step. Even so, the nested position of *Ferromirum* suggests that multiple symmoriiform lineages extended back into the Late Devonian (Fig. 5). Notably, this pattern is found throughout the major divisions of early members of the chondrichthyan crown-clade, and our results imply that much of the diversity of late Palaeozoic Chondrichthyes results from cladogenic events occurring before the Devonian-Carboniferous boundary.

### Anatomical significance

The exceptional 3D-preservation of the jaws and hyoid arch of *Ferromirum* provides insights into early chondrichthyan cranial structure and function. Initially using computer models with subsequent assembly of a physical model derived from STL files, we found that the jaws and hyoid apparatus could be rescaled to achieve a remarkably precise fit to the undistorted 3D-neurocranium of the much younger, early Permian symmoriiform *Dwykaselachus oosthuizeni*^3^ (Fig. 4a–d and Supplementary Fig. 3, 7, 8). This result both tests and corroborates an implicit assumption that the articulations between the neurocranium and the mandibular plus hyoid arches barely changed throughout the history of the Symmoriiformes^12,16^. Moreover, this striking morphological conservatism spanning a ~75 million-year age difference between these specimens, echoes genomic evidence of remarkably slow evolutionary rates within the holocephalan lineage relative to all other gnathostome clades^36^.

Importantly, in both *Ferromirum* as preserved (Figs. 1, 2) and in the *Ferromirum*-*Dwykaselachus* composite model (Fig. 4), the hyomandibula-ceratohyal joint is directly medial to the mandibular arch joint. This is quite unlike the recent interpretation of *Ozarcus*^16^, where the joints and arches are spaced one behind the other. Thus, unlike *Ozarcus*, *Ferromirum* includes no space for an enlarged pseudobranch-bearing spiracular pouch or fully respiratory gill pouch. The precise fit between the flanged rear of the ceratohyal and the ventromedial profile of Meckel’s cartilage shown in Fig. 1c is also present in an undescribed specimen of *Cobelodus aculeatus* (FMNH PF 7351) in the Field Museum (Chicago, IL) collection of sharks from the Mecca fauna of the Pennsylvanian black shales of central North America^5,6^. This specimen, like *Ferromirum*, is preserved in a barely disturbed posture with the ventral surface exposed. We suggest, after comparison with the extant frilled shark *Chlamydoselachus*, that this ceratohyal flange provided the origin of the mandibulo-ceratohyal ligament (the interpretation is based on position and proximity; *Chlamydoselachus*^37^ bears no such ceratohyal flange). Taken together, these data strongly imply that the apparent gap between mandibular and hyoid arches in *Ozarcus* and *Cobelodus*^5,16,38^ is an artefact of post-mortem lateral compression. Furthermore, rather than having a non-suspensory function, the symmoriiform hyoid arch is specialized and intimately involved in the jaw mechanism, *contra* the tenets of the aphetohyoidean hypothesis and related scenarios of visceral arch evolution^1,39–41^.

### Functional morphology of the mandibular and hyoid arches

**Fig. 6:**
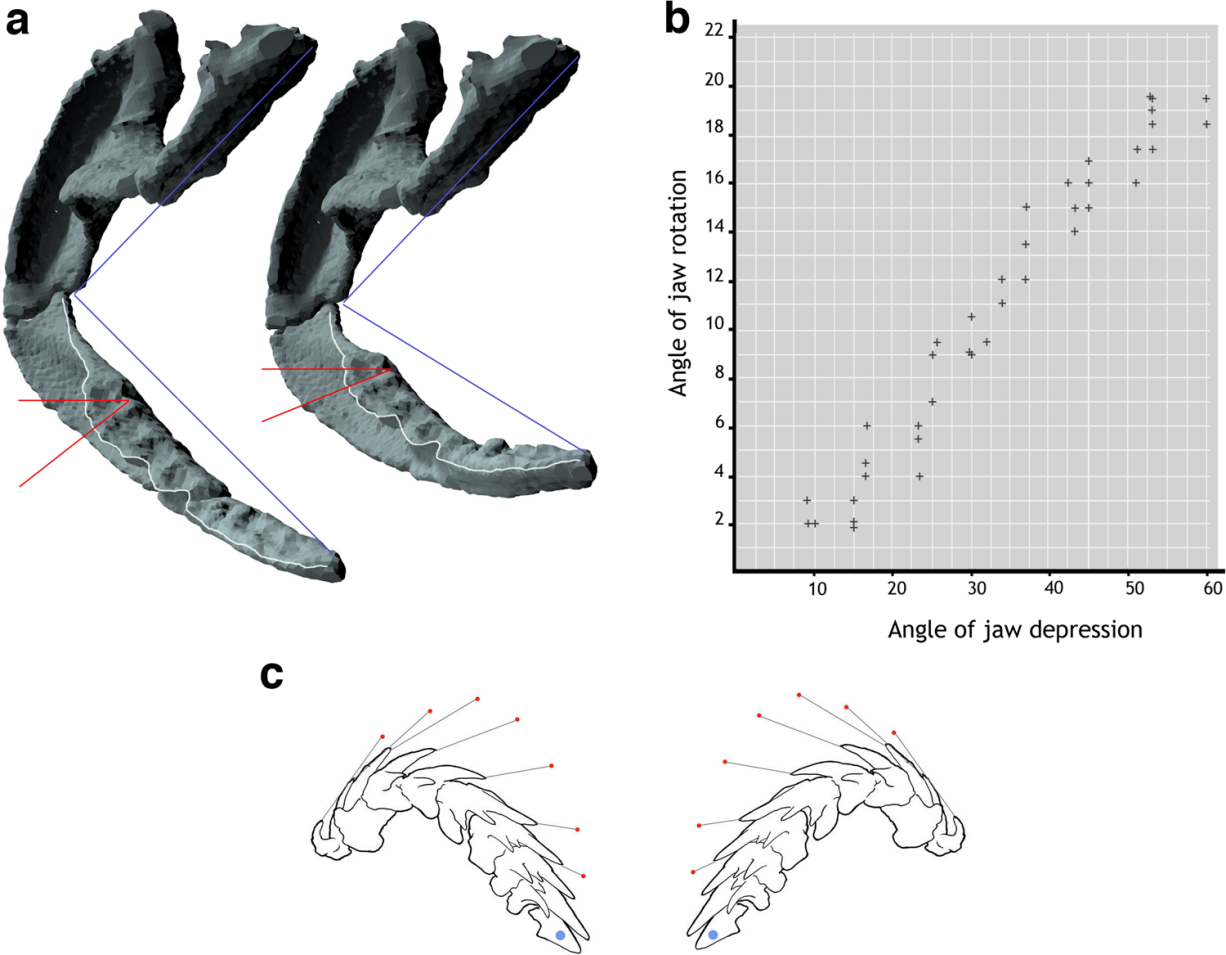
Jaw movement of *Ferromirum* gen. nov. **a** *Ferromirum* gen. et sp. nov. computer rendering of jaws in different degrees of Meckel’s cartilage depression, in anterolateral view. **b** Graphed angle of Meckel’s cartilage rotation relative to angle of depression, measurements taken from model in Fig. 5. **c** Line drawing of opposing lower jaw tooth sets, adapted from *Ozarcus*^16^. Lines and red dots and mark displacement of crowns when rotated through 22 degrees (graphic convention adapted from ref. ^44^).

The physical model (Fig. 4; Supplementary Fig. 8), combining hyoid and mandibular arches of *Ferromirum* and the braincase of *Dwykaselachus*, allowed direct investigation of jaw and hyoid arch motion. The amphistylic^42^, or, rather, archaeostylic^43^ mode of jaw suspension includes an articulation between the leading edge of the palatoquadrate otic process and the convex rear of the postorbital arcade, and between the palatoquadrate palatine ramus and the orbital articulation. In *Ferromirum*, the sigmoid profile of the jaw joint (Fig. 6a) forms a hinge, but, as noted in the description, the rotational axis between primary and secondary articulations is offset relative to the cardinal axes of the cranium (Supplementary Fig. 3). The resulting jaw motion seems counter-intuitive. Meckel’s cartilage rolls laterally (eversion: biting surface outwards) along its long axis while the jaw is opening, and medially (inversion: biting surface inwards) as the jaw closes (Fig. 6a, b). There is no broad mandibular symphysis to restrict this rotation during jaw depression and elevation (Fig. 4b). At a gape of 60 degrees, each side of the lower jaw has rolled outwards by approximately 20 degrees relative to its orientation when the jaws are closed (Fig. 6b), and the span of left and right mandibles in ventral view is slightly narrowed: the symphysial angle reduced from 50 (jaws closed) to 45 degrees (jaws open). Thus, as the gape opens, the dentition is everted and a greater proportion of the broad tooth battery is presented to the surrounding water column and prey. Comparison with analysis of tooth whorl function in *Helicoprion*^44^ suggests that older teeth, closer to being shed or displaced onto the lateral surface of the jaw, would have a slightly greater velocity advantage as the jaws close and the dentition is rolled inwards (the turning movement accelerated the more labial teeth more than the younger teeth during jaw closure). Such teeth are likely to impale or snag prey before such items are pushed or drawn into the mouth. Lingually located younger and larger, and thus less worn teeth might also became functional within the bite because of this rotation. Dental batteries of left and right sides would be rotated medially through a combined angle of 40 degrees (Fig. 6c), scooping material into the gape where the younger, newer, and hence more pointed teeth, deeper in the jaws with sharper and larger crowns, could be used to puncture and push prey deeper into the buccal space.

The role of the hyoid arch is not completely understood, but it is clear that it fits closely within the gently recessed rear of the palate and mandible. The archaeostylic palate might be considered self-supporting, but the hyomandibula likely served as an important structural brace limiting dorsoventral movement. Furthermore, manipulation of the model reveals that slight raising of the distal end of the hyomandibula contributes to depression of the ceratohyal and mandible.

**Fig. 7:**
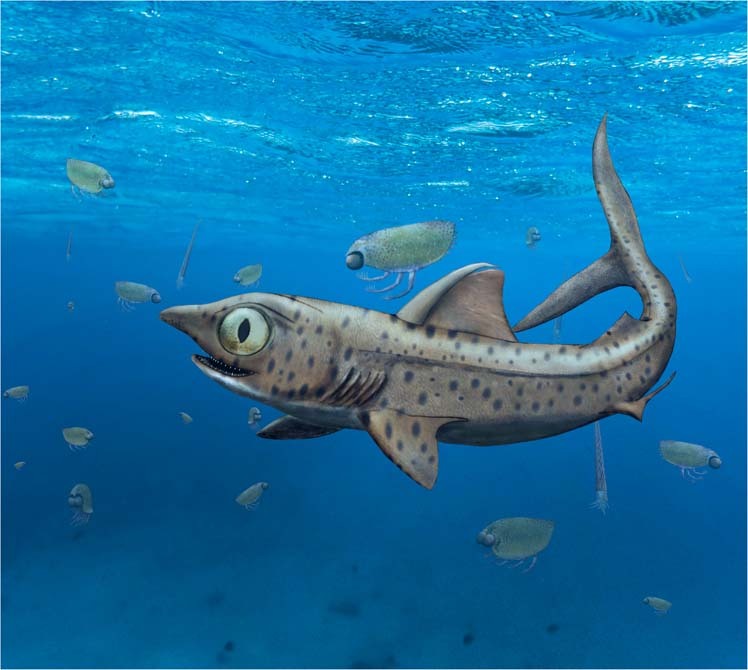
Reconstruction of *Ferromirum oukherbouchi* gen. et sp. nov. *Ferromirum oukherbouchi* gen. et sp. nov. reconstructed in association with invertebrates (orthocerid cephalopods and thylacocephalans: *Concavicaris submarinus*) from the Famennian of the Maider region (Morocco).

To the best of our knowledge, the pattern of jaw motion described here is unknown in living fish *sensu lato* However, a hemimandibular roll is considered crucial to oral processing in early mammals, and an important factor in the evolution of mammalian feeding systems^45^. Among Palaeozoic sharks, *Ferromirum* is quite unlike the specialized saw-jawed stem-holocephalan *Helicoprion*^44^ and the suction feeding stem-elasmobranch *Tristychius*^19^. However, key aspects of the feeding apparatus in *Ferromirum* are widespread among early chondrichthyans^1,2,5–8^: a slender ceratohyal, an elongate hyomandibula, a jaw joint far behind the orbit (at the extremity of a cleaver-shaped palate), and no trace of labial cartilages. For these reasons, we suggest that *Ferromirum* likely provides a glimpse of more general functional conditions in early sharks. Notably, in the Maïder Basin, *Ferromirum* is one of several chondrichthyans occurring within sediments rich in thylacocephalan crustaceans^23,46^ (Fig. 7). It appears likely that ram feeding, employing a large gape lined with generative sets of cladodont teeth, with those of the lower jaw rotating symmetrically inwards as the mouth snaps shut, would provide an effective means of capturing and retaining such seemingly abundant invertebrate prey.

Preservation quality has limited previous studies of early chondrichthyan jaw mechanics^43^ but insights from exceptionally preserved 3D-material might be used to identify morphological correlates of similar function among richer collections of flattened specimens. 2D-jaw shape measures of biomechanical traits have already been used to estimate functional disparity and implied ecological variety across early gnathostomes^47,48^. Thus, from the present work, the location of a mesial process (mandibular knob) relative to the primary articulation surface might be used similarly as a predictor of 3D jaw motion; especially hemimandibular long-axis rotation if also associated with a slender mandibular symphysis. In this regard, the jaws of symmoriiforms such as *Denaea* and *Symmorium*^6^ closely match those of *Ferromirum*. Moreover, the mandibular mesial process is a well-established chondrichthyan synapomorphy^47^ occurring deep within the stem lineage. It would be interesting to learn how the distribution of this process, and specialized jaw hinge, correlates with the evolution of the classic, tooth-whorl dominated, shark dentition.

## Methods

### Studied material

The type and only specimen (PIMUZ A/I 4806) of *Ferromirum oukherbouchi* gen. et sp. nov. is housed at the Palaeontological Institute and Museum of the University of Zurich, Switzerland. The specimen was prepared out of a ferruginous reddish nodule (rich in haematite) from the Famennian (Late Devonian) of Madene El Mrakib in the Maïder region of the southeastern Anti-Atlas (Morocco).

### Phylogenetic analysis

We performed a heuristic search in PAUP* 4.167^49^ using a parsimony ratchet^50^ with an initial 10,000 random sequence additions. The character matrix consists of 64 taxa, 56 ingroup and eight outgroup taxa, coded for 230 morphological characters. The analysis recovered 90 equally most parsimonious trees (MPTs) of 535 steps (consistency index 0.46; retention index 0.78; RC 0.36). We assessed nodal support through bootstrapping and Bremer Decay indices for the consensus tree. Please see Supplementary Note 1 and 2 for further details. The nexus-file is available via Dryad.

### X-ray micro-tomography

High resolution data were obtained using an industrial computed tomography scanner (Nikon XT H 225 ST) at the University of Zurich, Switzerland. Data acquisition and image reconstruction parameters are: 224 kV, 474 µA; filter: 4 mm of copper; isotropic voxel dimensions of 0.091 mm; 16-bit TIFF images were acquired; 1854 8-bit TIFF images were used for reconstruction. Images were analysed and 3D models reconstructed using Mimics v.17 (http://www.biomedical.materialise.com/mimics; Materialise, Leuven, Belgium). The 3D-object was edited (smoothing, colours and lightning) in MeshLab v. 2016 (http://www.meshlab.net;^41^) and blender v2.79b (https://www.blender.org; Amsterdam, Netherlands).

### Virtual and physical models

3D anatomical reconstructions of *Ferromirum* palatoquadrate, Meckel’s cartilage, hyomandibula and ceratohyal, and *Dwykaselachus* neurocranium used Mimics v. 18 (biomedical.materialise.com/mimics; Materialise, Leuven, Belgium) for three-dimensional modelling, STL polygon creation and kinematic simulations. Further editing of the STLs (colour, texture, lighting), kinematics and mirroring for the final restorations (virtual images, virtual manipulations, and 3D printouts of STL files) used 3D Studio Max (Autodesk.com/products/3ds-max; Autodesk, San Rafael, USA).

### Nomenclatural acts

This published work has been registered in ZooBank, the proposed online registration system for the International Code of Zoological Nomenclature (ICZN). The ZooBank LSIDs (Life Science Identifiers) can be resolved and associated information viewed through any standard web browser by appending LSID to the prefix “http://zoobank.org/.” The LSID for this publication is: urn:lsid:zoobank.org:pub:77DF56E3-58AC-4293-95B1-2E14F552D178.

### Reporting summary

Further information supporting the results and discussion of this study is available in the Nature Research Reporting Summary linked to this article.

## Supplementary information

Supplementary Information

Reporting Summary

## Data Availability

The authors declare that all data supporting the findings of this study are available at Dryad: 10.5061/dryad.qrfj6q5d2.
