# Supplementary material for: A symmoriiform from the Late Devonian of Morocco demonstrates a derived jaw function in ancient chondrichthyans
Source: Commun Biol. 2020 Nov 17;3:681. doi: 10.1038/s42003-020-01394-2 (PMC7672094; doi:10.1038/s42003-020-01394-2)
Supplement: Supplementary file 1 — Supplementary Information [file 42003_2020_1394_MOESM1_ESM.pdf]

Supplementary information

A symmoriiform from the Late Devonian of Morocco demonstrates a derived jaw function in ancient chondrichthyans

Linda Frey, Michael I. Coates, Kristen Tietjen, Martin Rücklin and Christian Klug

Content

Supplementary Figures 1 to 8	2
Supplementary Note 1 - Gape measurements	10
Supplementary Note 2 – Cladistic analyses	10
Taxon list	11
Character list	15
Syapomorphy list	28
Supplementary References	29

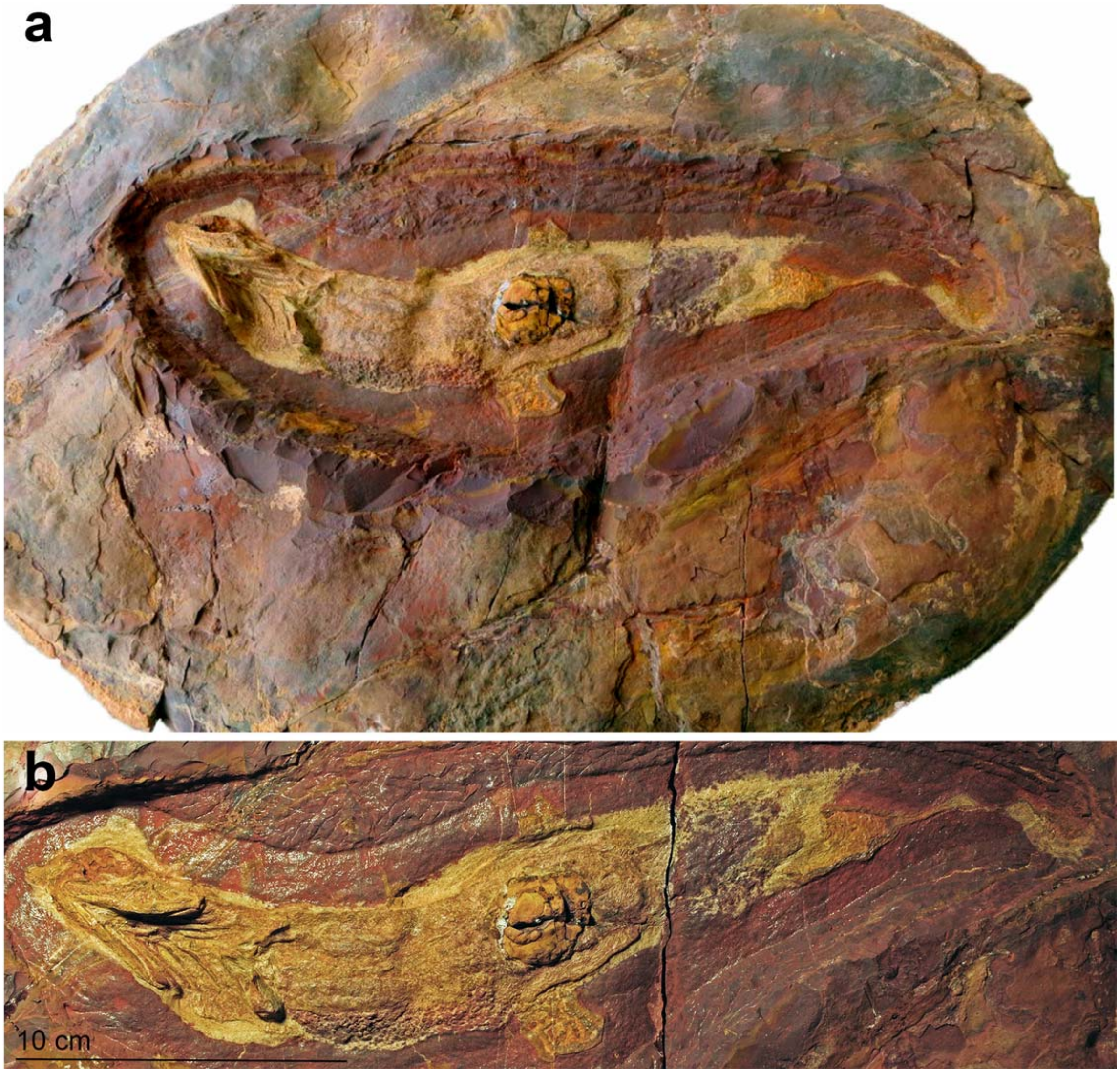

Supplementary Fig. 1. *Ferromirum oukherbouchi* gen. et sp. nov. Holotype. **a**, the entire nodule, which was later reduced to facilitate CT-scanning. **b**, detail of a.

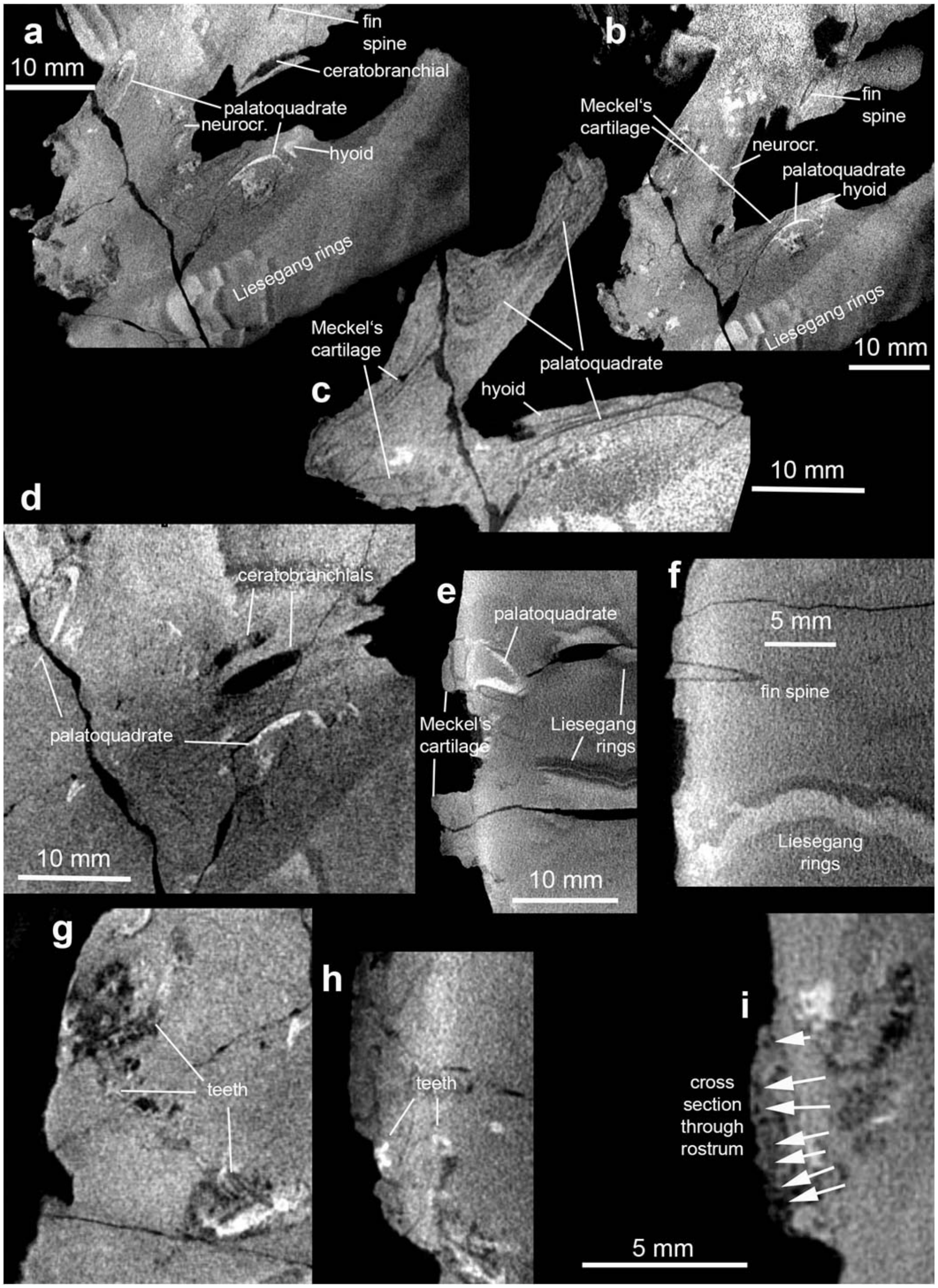

Supplementary Fig. 2. Images taken from the dcm-stack of *Ferromirum oukherbouchi* gen. et sp. nov. a to d, g, h, planes parallel to bedding. e, f, i, planes perpendicular to bedding and the plane of bilateral symmetry of the animal. Arrows in i point at the minute circular pits that might represent traces of electroreceptive or mechanoreceptive organs. g to i share the same scale bar at the bottom.

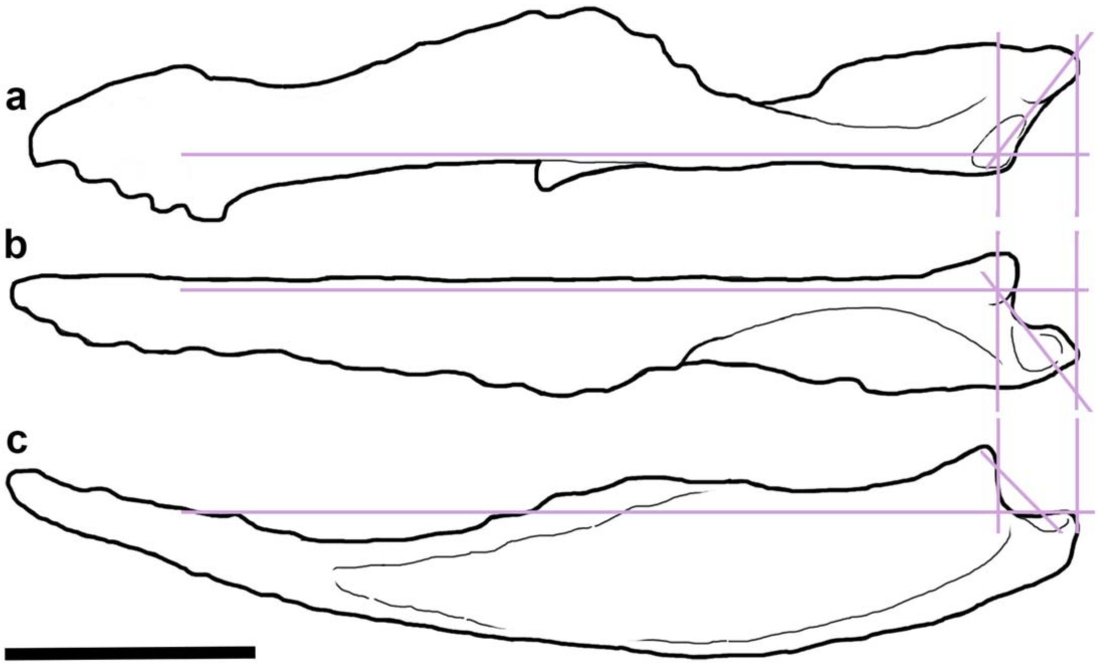

Supplementary Fig. 3. *Ferromirum oukherbouchi* gen. et sp. nov. Both jaws restored to show width of dental platform and offset angle of articulation surfaces. Scale bar is 10 mm. **a**, Palatoquadrate (left side) in ventral view. **b**, Meckel's cartilage (left side) in dorsal and, **c**, lateral view. Anterior to left of figure. For explanations of the method to measure the angle see Supplementary Note 1 - Gape measurements.

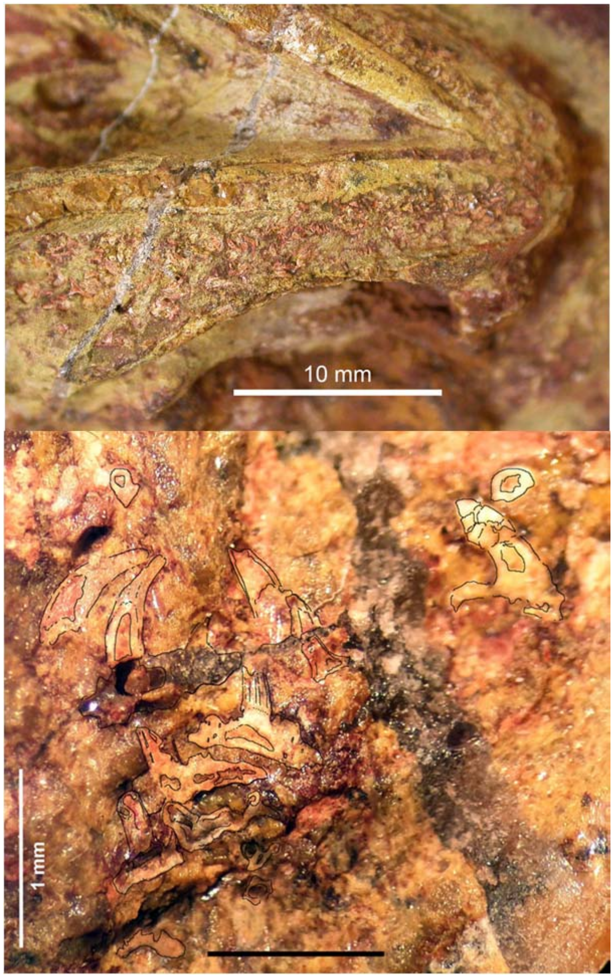

Supplementary Fig. 4. *Ferromirum oukherbouchi* gen. et sp. nov. dentition. Top: overview over left ramus of Meckel's cartilage with corroded dentition. Bottom: detail to show tooth morphology and tooth family arrangement. Morphological details traced with black lines.

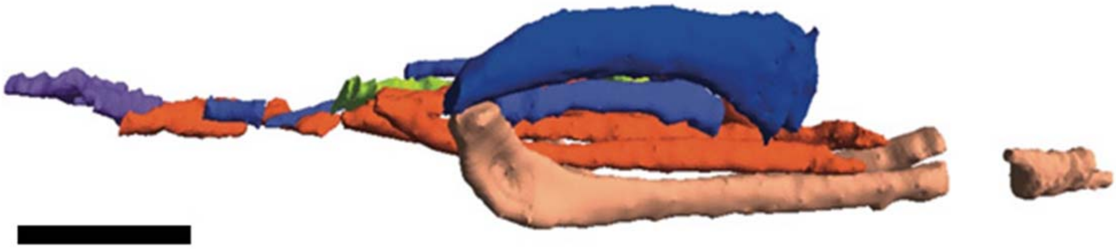

Supplementary Fig. 5. *Ferromirum oukherbouchi* gen. et sp. nov. hyoid arch and gill skeleton in lateral view (right side, anterior to right of figure). Scale bar is 10 mm long. Hyomandibula in dark blue; ceratohyal and hypohyal in pale brown.

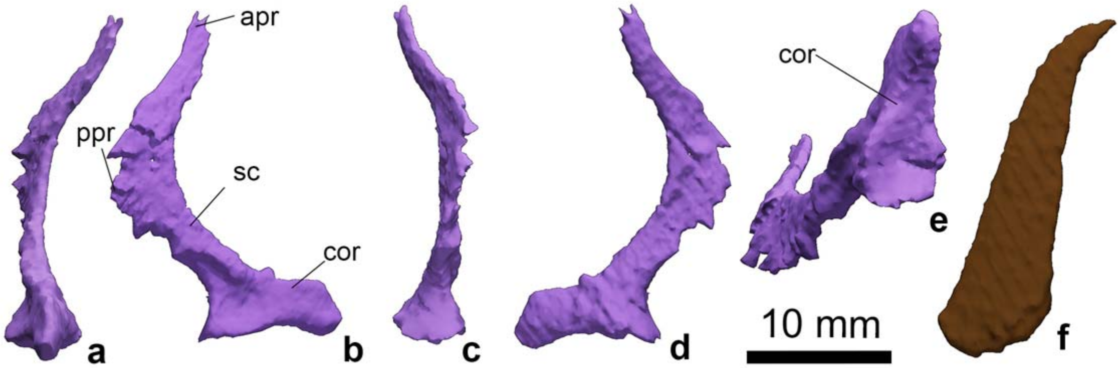

Supplementary Fig. 6. Pectoral girdle of *Ferromirum oukherbouchi* gen. et sp. nov. a to e, right scapulocoracoid in anterior (a), lateral (b), posterior and slightly dorsal (c), mesial (d), and ventral views. f, lateral view of dorsal fin spine; note the recurved tip. The transverse striation is an artefact from scanning and rendering. Abbreviations: apr (anterior process); cor (coracoid); ppr (posterior process); sc (scapula).

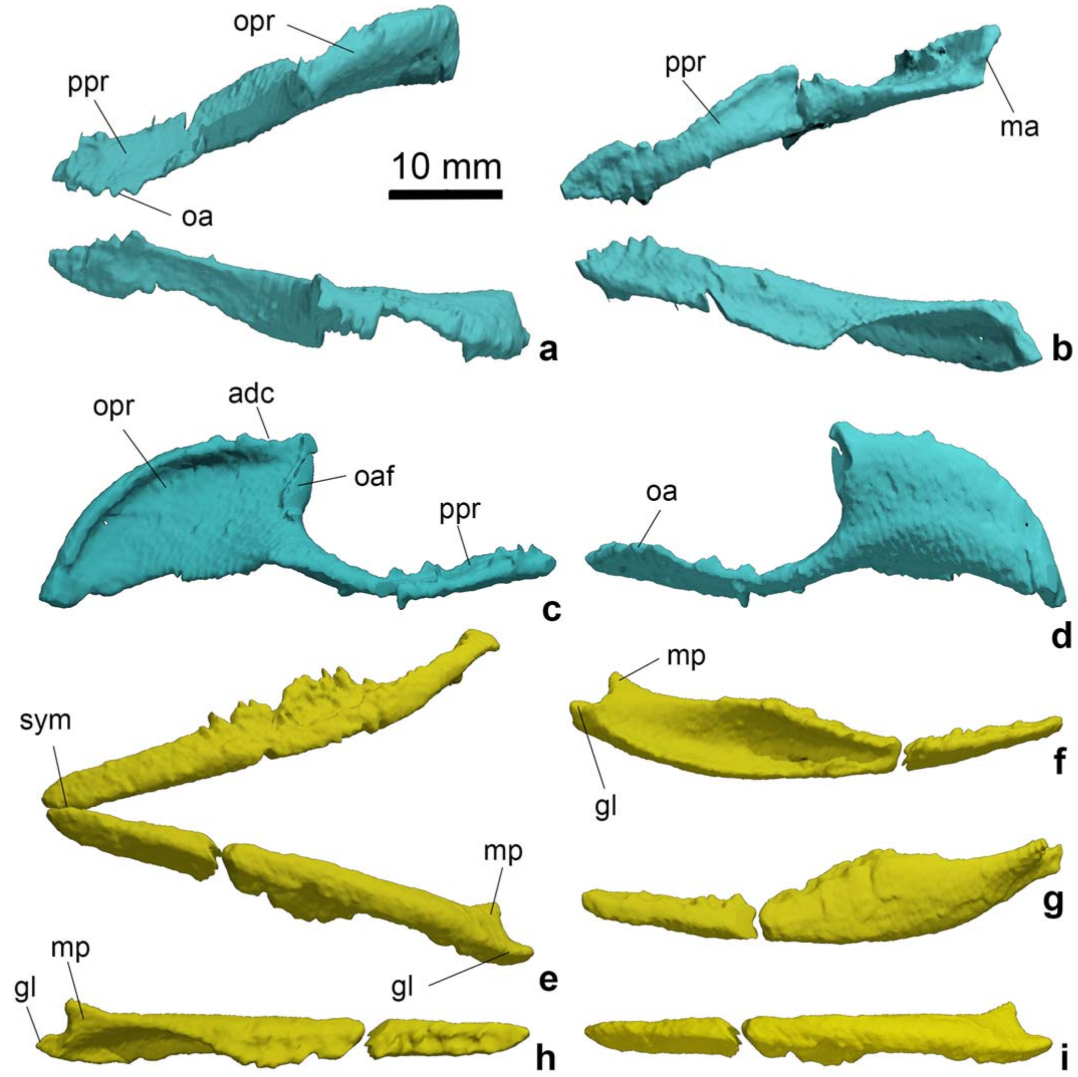

Supplementary Fig. 7. *Ferromirum oukherbouchi* gen. et sp. nov. jaws restored. a to d, palatoquadrates. a, dorsal, and b, ventral views. c, lateral (external) and d, mesial (internal) views of right palatoquadrate. e to i, Meckel's cartilage. e, ventral view. f, lateral (labial) view of right ramus. g, mesial (lingual) and slightly dorsal view of right ramus - hence mesial process of articulation level with glenoid (cotylus). h, dorsal view of right ramus. i, ventral view of right ramus. Abbreviations: gl (glenoid); ma (mandibular articulation); mp (mesial process); ppr (palatine process); oa (orbital articulation); oaf (otic articulation facet); opr (otic process); sym (symphysis).

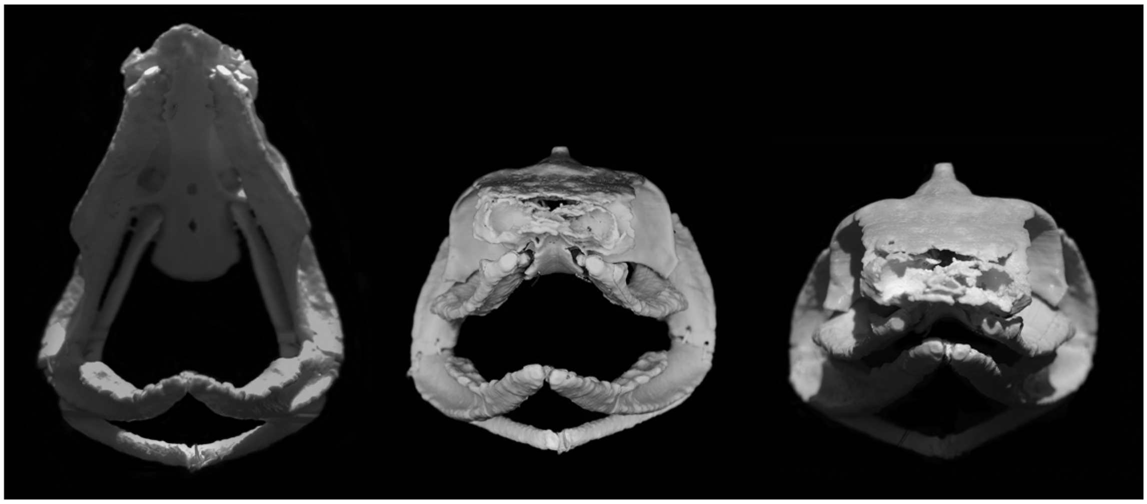

Supplementary Fig. 8. Composite model of *Ferromirum oukherbouchi* gen. et sp. nov. jaws and hyoid arch and *Dwykaselachus* neurocranium in anterior view, gapes of ~50 degrees, ~20 degrees, and closed.

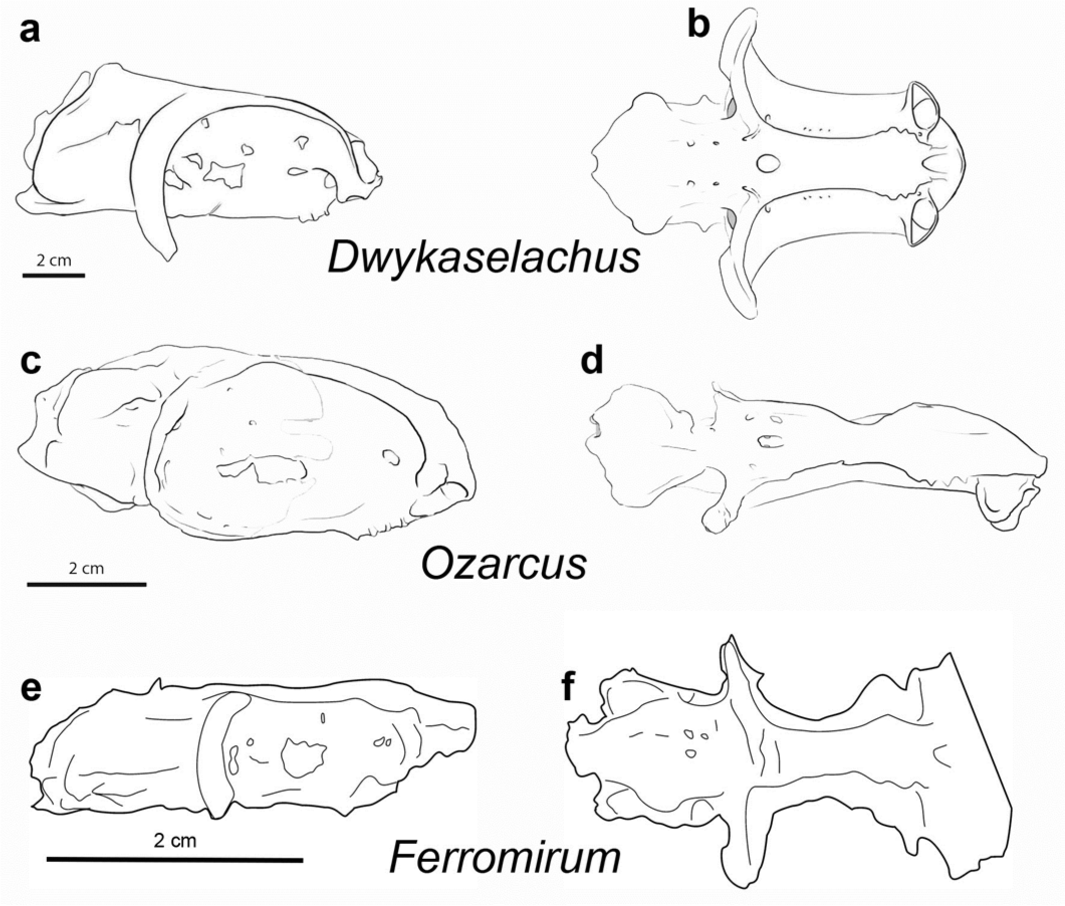

Supplementary Fig. 9. Comparison of neurocrania of the Palaeozoic chondrichthyans *Dwykaselachus* (a, b), *Ozarcus* (c, d), and *Ferromirum* (e, f). Except *Ferromirum*, all drawing from Coates et al. (2016). Right (b, d, f): right lateral views; left (a, c, e): ventral views.

SUPPLEMENTARY NOTE 1 – GAPE MEASUREMENTS

Gape angles and angles of lower jaw rotation (roll) were measured directly from the physical model of the composite symmoriid skull. For the gape angle, a horizontal rod was inserted between the quadrates at the level of the mesial articulation surface. The distance from the rod midpoint to the mandibular symphysis was measured (105 mm), and the same distance used to mark a midline location at the anterior of the palate (approximated by blue lines, Fig. 6a). A slender (2 mm diameter) wooden dowel rod of measured length was inserted to span the gap between mandibular symphysis and marked palatal location. This dowel was cut shorter by arbitrary lengths (recorded) so that the gape was reduced through a series of irregularly spaced, but measured, intervals.

For the rotation angle, a 60 mm rod was attached to the dental platform of Meckel's cartilage at the level of the recess of the second tooth set (counting from rear), where the platform is widest. The rod was fixed so that when the jaws were closed, the rod projected laterally and parallel to a second rod laid across both lower jaws at the same antero-posterior level. The distal tip of the second rod was aligned with that of the first. At different degrees of gape span (dictated by gape-spaced rod length), the distance between distal tips of these jaw rods varied (approximated by red lines, Fig. 6a) and was measured.

In both instances (gape and jaw rotation), measured sides of triangles were used to calculate angle subtended by lengthening or shortening side. The procedure was repeated using a series of 'gape' rods, with successive shortening. The scattered plot (Fig. 6b) reflects imperfections in the physical model: unevenness of the jaw joint articulation surfaces and wobble at the jaw joint.

SUPPLEMENTARY NOTE 2 – CLADISTIC ANALYSES

Bootstrap values (Felsenstein 1985) were calculated in PAUP* using the stepwise addition option with 10,000 replicates. Bremer decay indices were calculated through searches using 1,000 random sequence additions with a limit of 300,000 trees saved at each increment. At one extra step, crown group resolution is lost within a strict consensus of all trees. This is attributable to the inclusion of extremely incomplete taxa such as *Gogoselachus*, for which 82% of character scores are unknown, uncertain, or inapplicable. Notably, *Gogoselachus* exclusion has no effect on the topology of the strict consensus of most parsimonious trees. For this reason, the calculation of Bremer decay analyses excluded *Gogoselachus*. Character state transitions by node for the strict consensus cladogram of the MPTs were reconstructed in PAUP*[31] employing DELTRAN optimization, employing the complete taxon set.

Taxon and Character lists

Nexus file: **Ferro10 (inc.Gogo).nex**

Taxon list updates relative to Frey *et al*. 2019 matrix.

Gogoselachus: Long *et al*. 2015; *Gutterensis*: Sequeira & Coates 2000; *Gydoselache*: Maisey *et al*. 2019; *Helodus*: Patterson 1965; Stahl 1999; Coates *et al.* in press; Johanson *et al.* in press; *Ptomacanthus*: Brazeau 2009, 2012; Denison 1979; Miles 1973a; Dearden *et al*. 2019; *Tristychius*: Dick1978; Coates & Gess 2007; Coates & Tietjen 2018; Coates *et al*. 2019.

Character list updates relative to Frey *et al*. 2019 matrix.

Discussion, notes, and references supplied in character list.

16. Cranial sensory line canal passes between or beneath scales (0); passes over scales and/or is partially enclosed or surrounded by scales (1); perforates and passes through scales (2).

17. Postcranial sensory line canal passes between or beneath scales (0); passes over scales and/or is partially enclosed or surrounded by scales (1); perforates and passes through scales (2).

47. Opercular cover of branchial chamber complete or partial (0); separate gill covers and gill slits (1).

49. Gill skeleton mostly beneath otico-occipital region (0); mostly posterior to occipital region (1).

50. First branchial arch meets neurocranium ventral to otic region (0); posterior to otic region (1).

53. Ceratohyal with posterior/proximal external fossa: absent (0); present (1).

54. Ceratohyal with broad posteroventral flange or shelf, projecting laterally into recess in Meckel's cartilage: absent (0); present (1).

55. Ceratohyal spatulate or bladed anteriorly: absent (0); present (1).

56. Hypohyals: absent (0); present (1).

57. Basihyal: absent, hyoid arch articulates directly with basibranchial (0); present (1).

63. Multiple unpaired basibranchial mineralisations absent (0); present (1).

64. Elongate posterior copula projects posteriorly, beyond rearmost branchial arch: absent (0); present (1).

96. Meckel's cartilage with flange or shelf projecting posteriorly from the lateral cotylus (glenoid): absent (0); present (1).

112. Large prootic foramen separated from optic fenestra by antotic pillar bearing optic pedicel: absent (0); present (1).

177. Posterior openings of lateral aortic canals positioned lateral to occipital cotylus: absent (0); present (1).

218. Dorsal fin spine apex curved posteriorly: absent (0); present (1).

Taxa and Sources

Acanthodes: Benznosov 2009; Brazeau & de Winter 2015; Coates 1994; Davis *et al.* 2012; Heidtke 1993, 2011a,b; Jarvik 1977, 1980; Miles 1968, 1973a, b; Nelson 1968; Watson 1937.

Acronemus: Maisey 2011; Rieppel 1982.

Akmonistion: Coates & Sequiera 1998, 2001a, b; Coates *et al.* 1998; Coates et al. 2017.

Brachyacanthus: Denison 1979; Miles 1973a; Watson 1937.

Brochoadmones: Bernacsek & Dineley 1977; Gagnier & Wilson 1996b; Hanke & Wilson 2006.

Callorhinchus/Hydrolagus: Cole 1896; De Beer 1937; De Beer & Moy-Thomas 1935; Didier 1995; Didier *et al*. 1994, 1998, 2012; Howard *et al*. 2013; Kesteven 1937; Patterson 1965, 1992; Pradel *et al*. 2013; Stahl 1999.

Cheiracanthus: Denison 1979; Miles 1973a; Watson 1937.

Cheirolepis: Arratia & Cloutier 1996; Pearson & Westoll 1979; Giles *et al*. 2015a.

Chondrenchelys: Finarelli & Coates 2012, 2014; Lund 1982; Moythomas 1935.

Cladodoides: Gross 1937, 1938; Maisey 2005.

Cladoselache: Bendix-Almgreen 1975; Harris 1938a, b; Maisey 1989a, 2007; Schaeffer 1981; Williams 2001; Woodward & White 1938.

Climatius: Miles 1973a, b; Watson 1937.

Cobelodus: Zangerl & Case 1976; Zidek 1992.

Culmacanthus: Long 1983.

Damocles: Lund 1986.

Debeerius: Grogan & Lund 2000.

Diplacanthus: Gagnier 1996; Miles 1973a; Watson 1937.

Diplodoselache: Dick 1981.

Doliodus: Miller *et al.* 2003; Maisey *et al.* 2009, 2013, 2017; Long *et al*. 2015.

Dwykaselachus: Coates *et al.* 2017; Oelofsen 1986.

Egertonodus: Maisey 1982, 1983; Lane, 2010.

Entelognathus: Zhu *et al*. 2013.

Ferromirum oukherbouchi gen. et sp. nov.: holotype specimen PIMUZ XX.

Gladbachus: Heidtke & Krätschmer 2001; Heidtke 2009; Burrow & Turner 2013; Coates 2005; Coates *et al*. 2018.

Gogoselachus: Long *et al*. 2015.

Guiyu: Zhu *et al*. 2009.

Gutterensis: Sequeira & Coates 2000.

Gydoselache: Maisey *et al*. 2019.

Gyracanthides: Miles 1973a; Warren *et al*. 2000; Turner *et al*. 2005.

Halimacanthodes: Burrow *et al*. 2012.

Hamiltonichthys: Maisey 1989b.

Helodus: Patterson 1965; Stahl 1999; Coates *et al.* in press; Johanson *et al.* in press.

Homalacanthus: Gagnier 1996; Watson 1937.

Homalodontus: Mutter *et al*. 2007, 2008.

Iniopera: Zangerl & Case 1973; Pradel *et al*. 2009b, Pradel 2010; Pradel *et al*. 2010.

Ischnacanthus; Miles 1973a; Watson 1937.

Kawichthys: Pradel *et al*. 2011.

Kathemacanthus: Gagnier & Wilson 1996a; Hanke & Wilson 2010.

Latviacanthus: Schultze & Zidek 1982.

Lupopsyrus: Hanke & Davis 2012; Bernacsek & Dineley 1977.

Mesacanthus: Miles 1973a; Watson 1937.

Mimipiscis: Gardiner & Bartram 1977; Gardiner 1984; Choo 2011; Giles & Friedman 2014.

Moythomasia: Gardiner & Bartram 1977; Gardiner 1984; Coates et al. 2017; specimen MV P222915.

Obtusacanthus: Hanke & Wilson 2004; specimen UALVP 41488.

Onychoselache: Dick & Maisey 1980; Coates & Gess 2007.

Orthacanthus: Hampe 2003; Heidtke 1982, 1999; Hotton 1952; Schaeffer 1981; Maisey 1983; Lane & Maisey 2009.

Ozarcus and FMNH PF 13242: Maisey 2007; Pradel *et al.* 2014; Coates *et al*. 2017.

Parexus: Watson 1937; Miles 1973a.

Phoebodus: Frey *et al*. 2019

Psarolepis: Yu 1998; Zhu & Schultze 1997; Zhu *et al.* 1999; Qu *et al.* (2013a).

Ptomacanthus: Brazeau 2009, 2012; Denison 1979; Miles 1973a; Dearden *et al*. 2019.

Pucapampella: Maisey 2001a; Maisey & Anderson 2001; Maisey & Lane 2010; Janvier & Maisey 2010; Maisey *et al*. 2019.

Raynerius: Giles *et al*. 2015b.

Rhadinacanthus: Burrow *et al*. (2016).

Squalus: Schaeffer 1981; Gans & Parsons 1964; Marinelli & Strenger 1959.

Synechodus: Maisey 1985.

Tamiobatis: Schaeffer 1981.

Tetanopsyrus: Gagnier & Wilson 1995; Gagnier *et al*. 1999; Hanke *et al.* 2001.

Thrinacodus: Grogan & Lund 2008**.**

Tribodus: Maisey & de Carvalho 1997; Lane 2010; Lane & Maisey 2009, 2012.

Triodus: Solér-Gijon, R. & Hampe 1998; Hampe 2003; Heidtke *et al*. 2004.

Tristychius: Dick1978; Coates & Gess 2007; Coates & Tietjen 2018; Coates *et al*. 2019.

Uraniacanthus: Bernacsek & Dineley 1977; Hanke & Davis 2008; Newman *et al.* 2012; Burrow *et al*. 2016.

Youngolepis: Chang & Yu 1981; Chang 1982, 1991, 2004.

Characters

Skeletal tissues

1. **Tessellate calcified cartilage: absent (0); present (1**). Dean & Summers (2006); Dean *et al*. (2009); Maisey (1984, 2001, 2013); Seidel *et al*. (2016) Lund & Grogan (1997, 2004a, b); Coates & Sequeira (2001a, b); Brazeau (2009); Davis *et al.* (2012); Grogan *et al*. (2012); Pradel *et al.* (2014).

2. **Perichondral bone: present (0); absent (1).** Janvier (1996); Donoghue & Aldridge (2001); Brazeau (2009); Davis *et al.* (2012).

3. **Extensive endochondral ossification: absent (0); present (1).** Forey (1980); Gardiner (1984); Brazeau (2009); Davis *et al.* (2012).

4. **Extensive calcified cartilage: absent (0); present (1).** To capture all taxa in which the neurocranium, jaws, hyoid and gill arches, as well as parts of the axial and appendicular skeleton are mineralized in the absence of perichondral bone.

5. **Tubular dentine: absent (0); present (1).** Stahl (1999), see also Patterson (1965): present in chimaeroids, edestids, *Helodus*, and petalodonts, but absent in symmoriids and iniopterygians (Zangerl & Case 1973).

6. **Pore canal network: absent (0); present (1).** Lu *et al*. (2016).

7. **Acrodin tooth caps (enameloid cap restricted to crown apex): absent (0); present (1).** Friedman & Brazeau (2010); Zhu *et al.* (2013, 2009); Lu *et al*. (2016).

Squamation & related structures

8. **Trunk scales monocuspid (0); multicuspid (1).** Revised after Davis *et al.* (2012); see Burrow *et al*. (2016) and Coates *et al.* (2018).

9. **Scale growth concentric: absent (0); present (1).** Hanke & Wilson (2004); Brazeau (2009); Davis *et al.* (2012); Burrow *et al*. (2016).

10. **Peg-and-socket articulation: absent (0); present (1).** Gardiner (1984); Coates (1999); Brazeau (2009); Davis *et al.* (2012).

11. **Anterodorsal process on scale: absent (0); present (1).** Zhu *et al.* (2009, 2013); Gardiner (1984); Coates (1999); Brazeau (2009); Davis *et al.* (2012). Notably present in *Cheirolepis*, see Pearson and Westoll (1979).

12. **Body scales with bulging base: absent (0); present (1).** Brazeau (2009); Davis *et al.* (2012); Burrow *et al*. (2016).

13. **Body scales with flattened base: absent (0); present (1).** Brazeau (2009) ; Davis *et al.* (2012); Burrow *et al*. (2016). Note that both flat- and bulge-based scales occur in conjunction in *Ptomacanthus* (Brazeau 2012).

14. **Body scales with basal canal or open basal vascular cavity: absent (0); present (1).** Chondrichthyan placoid scales are distinguished, in part, by the presence of a basal canal (Reif 1978). Early chondrichthyan scales that depart from the standard placoid morphology also exhibit basal canals, e.g. *Akmonistion* (Coates & Sequeira 2001a, fig. 12E) and *Antarctilamna* (Young 1982, p.830). Basal canals are present in putative chondrichthyan scale forms such as *Elegestolepis* (Karatajūte-Talimaa 1992), *Polymerolepis* and *Seretolepis* (Hanke & Wilson 2010).

15. **Neck canal: absent (0) present (1).** Although a standard feature of placoid scales, presence of a neck canal is widespread among early vertebrates: Coates *et al.* (2018).

16. **Cranial sensory line canal passes between or beneath scales (0); passes over scales and/or is partially enclosed or surrounded by scales (1); perforates and passes through scales (2).** Character revised after Dearden *et al*. (2019).

17. **Postcranial sensory line canal passes between or beneath scales (0); passes over scales and/or is partially enclosed or surrounded by scales (1); perforates and passes through scales (2).** Character revised after Dearden *et al*. (2019).

18. **Lepidotrichia: absent (0); present (1).**

19. **Fringing fulcra: absent (0); present (1).** Zhu *et al.* (2009; 2013); Coates (1999). Scored inapplicable in taxa lacking lepidotrichia.

20. **Scute-like ridge scales (fulcra): absent (0); present (1).** Giles *et al.* (2015c).

Cranial dermal skeleton

21. **Cranial cap denticles, single-crowned, non-growing: absent (0); present (1).** Scored absent in *Ferromirum*; if present, it appears likely that they would have been preserved.

22. **Sclerotic ring: absent (0); present (1).** Giles *et al*. (2015, c.52), Qiao *et al*. (2016, c.277), Zhu *et al*. (2016, c.275); Burrow *et al.* (2016); Coates et al. (2018).

23. **Number of sclerotic plates: four or less (0); more than four (1).** Zhu *et al*. (2013, c170); Qiao *et al*. (2016, c.241); Zhu *et al*. (2016, c.239); Burrow *et al.* (2016).

24. **Dermal skull roof includes large dermal plates (0); consists of plates, tesserae or scales (1); naked or largely scale free (2).** Forey (1980); Gardiner (1984); Brazeau (2009); Davis *et al.* (2012); Zhu *et al.* (2013); Brazeau & Friedman (2014).

25. **Dermal ornamentation: smooth (0); parallel, vermiform ridges (1); concentric ridges (2); tuberculate (3).** Giles *et al*. (2015c). Coded inapplicable where dermal plates absent.

26. **Cranial tessera morphology: large interlocking plates (0); microsquamose, no larger than body squamation (1).** Brazeau (2009) through to Giles *et al.* (2015c). Coded inapplicable where tesserae are absent.

27. **Anterior or mesial edge of nasal notched for anterior nostril: absent (0); present (1)**. Contra Zhu *et al.* (2013), the anterior rim of the nasal in *Cheirolepis* is notched.

28. **Supraorbital: absent (0); present (1).** Zhu *et al.* (2009, 2013).

29. **Broad supraorbital vaults: absent (0); present (1).** Dennis & Miles (1981); Giles *et al.* (2015c). As noted by Giles *et al*., this character is contingent on the presence of a dermal skull roof composed of large dermal bones.

30. **Large median bone contributes to posterior margin of skull roof: absent (0); present (1).** Zhu *et al.* (2016).

31. **Pineal opening perforates dermal skull roof: present (0); absent (1).** Davis *et al.* (2012); Giles *et al.* (2015c).

32. **Consolidated cheek plates: absent (0); present (1).** Davis (2002); Brazeau (2009); Davis *et al.* (2012); Zhu *et al.* (2013); Burrow *et al.* (2016).

33. **Enlarged postorbital tessera separate from orbital series: absent (0); present (1).** Brazeau (2009); Davis *et al.* (2012); Zhu *et al.* (2013); Burrow *et al.* (2016).

34. **Dermal intracranial joint: absent (0); present (1).** Zhu *et al.* (2009, 2013).

35. Sensory line network preserved as open grooves (sulci) in dermal bones (0); sensory lines pass through canals enclosed within dermal bones (1). (Davis 2002); Davis *et al.* (2012); Zhu *et al.* (2013).

36. Sensory canal or pit-line associated with maxilla: absent (0); present (1). Contra Zhu *et al.* (2013) this structure is not reported or figured in *Mimipiscis* (Gardiner 1984, Choo 2011); Friedman (2007) notes correctly that this feature is present in onychodonts, *Moythomasia* and *Cheirolepis*.

37. Jugal portion of infraorbital canal joins supramaxillary canal: present (0); absent (1). Brazeau (2009), but see redefinition in Davis *et al.* (2012); Zhu *et al.* (2013).

38. Anterior pit line of skull roof: absent (0); present (1). Giles *et al.* (2015c).

39. Spiracular opening in dermal skull roof bounded by bones carrying otic canal: absent (0); present (1). Giles *et al*. (2015); Lu *et al*. (2016).

40. Dermohyal (submarginal) ossification: absent (0); present (1). Alternative homology hypotheses discussed in Coates *et al*. (2018).

41. Branchiostegal series: absent (0); present (1). Davis (2002); Hanke & Wilson (2004); Brazeau (2009); Davis *et al.* (2012); Zhu *et al.* (2013); Burrow et al. (2016).

42. Opercular and subopercular bones: absent (0); present (1). Scores for opercular bones contingent on branchiostegal series presence.

43. Branchiostegal plate series along ventral margin of lower jaw: absent (0); present (1). Davis (2002); Hanke & Wilson (2004); Brazeau (2009); Davis *et al.* (2012); Zhu *et al.* (2013).

44. Branchiostegal ossifications plate-like (0); narrow and ribbon-like (1); filamentous (2). Hanke & Wilson (2004); Brazeau (2009); Davis *et al.* (2012); Lu *et al.* (2016).

45. Branchiostegal ossifications ornamented (0); unornamented (1). Brazeau (2009); Davis *et al.* (2012); Zhu *et al.* (2013).

46. Branchiostegals imbricated: absent (0); present (1). Brazeau (2009); Davis *et al.* (2012); Zhu *et al.* (2013).

47. Opercular cover of branchial chamber complete or partial (0); separate gill covers and gill slits (1). Scores revised after Dearden *et al*. (2019), Watson (1937) and reference to specimen NHMUK P49979. Although *Acanthodes* has an elongate pharynx, with the gill skeleton arranged postcranially much as in a modern elasmobranch, it appears that an extended opercular flap was present. As noted by Watson (1937, p.110), a vertical portion of a scale-enclosed opercular sensory canal passes vertically, uninterrupted, across the more posterior members of the gill arch series. The canal must have been flap-supported. Close examination of NHMUK P49979 confirms this feature.

48. Gular plates: absent (0); present (1). Gardiner (1984); Brazeau (2009); Davis *et al.* (2012); Zhu *et al.* (2013).

Hyoid and gill arches

49. Gill skeleton mostly beneath otico-occipital region (0); mostly posterior to occipital region (1). Zangerl (1981); Lund & Grogan (1997); Stahl (1999). Both conditions (states '0' and '1') are likely present among acanthodians; scores have been revised to be consistent with Dearden *et al*. (2019).

50. First branchial arch meets neurocranium ventral to otic region (0); posterior to otic region (1). Included after Dearden *et al*. (2019), but scores differ. In *Acanthodes* NHMUK specimens P34914, P49979, and P49990, the first branchial arch is positioned posterior to the hyoid arch. The head of the anteriormost branchial arch is post-otic, and not nested within or

below the hyoid arch, as reconstructed by Dearden *et al*. (2019, Fig. 3). Dearden *et al*. score *Cladoselache* and *Cobelodus* as state '0'; here, the scores are revised to state '1'.

51. Perforate hyomandibula: absent (0); present (1). Zhu *et al.* (2009, 2013); Lu *et al.* (2016).

52. Interhyal: absent (0); present (1). Davis *et al.* (2012); Zhu *et al.* (2013).

53. Ceratohyal with posterior/proximal external fossa: absent (0); present (1). Posteriorly, the ceratohyal curves, to a greater or lesser extent, both dorsally and laterally. The lateral surface encloses a hollow: a distinct, broad fossa. Fossil examples include *Tristychius* (Coates *et al*. 2019) *Egertonodus* (Maisey, 1983), *Orthacanthus* (Hotton, 1952; pers. obs. M.I.C.), *Gogoselachus* (Long *et al*. 2015), *Cladoselache* (Maisey 1989), and as shown here, *Ferromirum*. Further examples are evident in the partly crushed ceratohyals in specimens of *Akmonistion* (Coates & Sequeira 2001), and the similarly preserved ceratohyal of *Gladbachus* (Coates *et al*. 2018, supplementary fig. 7b).

54. Ceratohyal with broad posteroventral flange or shelf, projecting laterally into recess in Meckel's cartilage: absent (0); present (1). Present in *Ferromirum*, this flange is likely also present in *Cladoselache*, identified as a 'ventral process' (Maisey 1989). In *Ferromirum*, the flange fits snugly into a smooth recess within the posteroventral extremity of the medial surface of Meckel's cartilage. Such a flange is also clearly present in *Cobelodus aculeatus* PF 7351 (not figured, but listed in Zangerl & Case 1976 as nearly whole, articulated skeleton, lacking tail), and is likely present in many symmoriids. Like that of *Ferromirum*, the flange engages with a ventrolateral recess in the posterior of Meckel's cartilage (personal observation, MIC). See also *Debeerius* (Grogan & Lund 2000, fig. C,D,E): a similarly broad flange is present, although the specimen is flattened.

55. Ceratohyal spatulate or bladed anteriorly: absent (0); present (1). Present in *Tristychius* and *Phoebodus*. Alternative characterisation: in general, main shaft of the ceratohyal is laterally compressed and/or slender. The apomorphic condition is to have a dorsoventrally flattened and anteriorly expanded shaft. *Ferromirum* exhibits the laterally compressed, elongate condition.

56. Hypohyals: absent (0); present (1). Friedman & Brazeau (2010); Pradel *et al.* (2014); scores updated after Dearden *et al*. (2019). Notably, the reconstructed arrangement in *Triodus* (Heidtke *et al*. 2004) showing large hypohyals is consistent with details in photograph of the type specimen of *T. sessilis* (Hampe, 2004, Fig. 18). However, such hypohyals appear absent in *Orthacanthus* (Heidtke 1999, Abb.5), a less derived member of the xenacanth clade (Hampe, 2004). Presence in *Ferromirum* is consistent with conditions *Ozarcus*, *Cobelodus*, and *Akmonistion*.

57. Basihyal: absent, hyoid arch articulates directly with basibranchial (0); present (1). Pradel *et al.* (2014); Carr *et al.* (2009); Brazeau *et al.* (2017); Dearden *et al*. (2019) in part. Acknowledging the discussion in Dearden *et al.*, here the definition of a basihyal is the unpaired, median unit of the hyoid arch, separated from other basibranchials, and in some taxa also articulating with the first branchial arch. Thus, a basihyal is distinguished from the elongate single basibranchial of early osteichthyans. A T-shaped basihyal is present in *Acanthodes* (Watson 1937; Heidtke 2011), resembling the similarly formed basihyal in *Orthacanthus* (Heidtke 1999).

58. Separate supra- and infra-pharyngobranchials absent (0); present (1). Gardiner (1984); Pradel *et al*. (2014). Scored as uncertain for *Ferromirum*.

59. Pharyngobranchials directed anteriorly (0); posteriorly (1). Pradel *et al.* (2014). Scored as uncertain for *Ferromirum.*

60. Posteriormost branchial arch bears epibranchial unit: absent (0); present (1). Scored as uncertain for *Ferromirum.*

61. Epibranchials bear posterior flange: absent (0); present (1).

62. **Hypobranchials directed anteriorly (0); hypobranchials of second and more posterior gill arches directed posteriorly (1).**

63. **Multiple unpaired basibranchial mineralisations absent (0); present (1).** Included after Dearden *et al*. (2019), but scores differ. Among the hybodontids, *Hamiltonichthys* is scored present; conditions in other hybodontids are unknown. The xenacanth *Triodus* is scored absent rather than present (Heidtke *et al*., 2004, identify a series of paired hypobranchials as basibranchials; revision here consistent with Dearden et al. 2019 Fig. 4h), but *Orthacanthus* is scored present rather than unknown.

64. **Elongate posterior copula projects posteriorly, beyond rearmost branchial arch: absent (0); present (1).** Present in many early chondrichthyans, including *Ferromirum*, *Gutturensis*, *Debeerius*, and *Gladbachus*; also manifest in modern chimaeroids and elasmobranchs. Thus, this possibly constitutes an autapomorphy at some level of the chondrichthyan clade. Notably absent in *Tristychius* and xenacanths.

Dentition & tooth-bearing bones

65. **Oral dermal tubercles borne on jaw cartilages: absent (0); present (1).** Hanke & Wilson (2004); Brazeau (229); Davis *et al.* (2012); Zhu *et al.* (2013). This character is concerned only with position and form, and not with histological, and by inference, developmental distinctiveness (cf. Rücklin *et al.* 2012).

66. **Pharyngeal teeth or denticles: absent (0); present (1).**

67. **Tooth families/generative tooth sets: absent (0); present (1).** Davis (2002); Hanke & Wilson (2004); Brazeau (2009); Davis *et al.* (2012); Zhu *et al.* (2013).

68. **Tooth families/generative sets restricted to symphysial region (0); distributed along jaw margin (1).** Hanke & Wilson (2004); Brazeau (2009); Davis *et al.* (2012); Zhu *et al.* (2013). Revised in light of discussion by Tucker and Fraser (2013): tooth whorl restricted to symphysial region appears to be the more general condition, whereas tooth whorls distributed continuously along the gape margin likely represents a chondrichthyan synapomorphy.

69. **Number of generative tooth sets per jaw ramus: 15 or fewer (0); 20 or more (1).**

70. **Bases of tooth families/generative sets: single, continuous plate (0); some or all whorls consist of separate tooth units (1).** Adjusted from Brazeau (2009); Davis *et al.* (2012); Zhu *et al.* (2013); Giles *et al*. (2015).

71. **Lingual torus: absent (0); present (1).** This platform or ledge (Ginter *et al.* 2010) is notably absent in early elasmobranchs and many of the non-toothplate dentitions of stem holocephalans.

72. **Basolabial shelf: absent (0); present (1).** (Ginter *et al.* 2010.)

73. **Teeth with three slim main cusps almost equal to each other, strongly recurved: absent (0); present (1).** Adapted from Ginter *et al*. (2010).

74. **Toothplates absent (0); present (1).** Patterson (1965); Stahl (1999).

75. **Toothplates consolidated into one to three large posterior plates, and one to three smaller anterior tooth plates, occupying each quadrant of the jaw: absent (0); present (1).** Adapted from Stahl (1999), the character captures conditions in chondrenchelyids, cochliodonts, and more derived taxa.

76. **Toothplate complement restricted to two pairs in the upper jaw and a single pair in the lower jaw: absent (0); present (1).** After Patterson (1965).

77. **Mandibular teeth fused to dermal plates on biting surfaces of jaw cartilages: absent (0); present (1).** Hanke & Wilson (2004); Brazeau (2009); Davis *et al.* (2012); Zhu *et al.* (2013).

78. Dermal plates on biting surface of jaw cartilages: absent (0); present (1). Brazeau (2009); Davis *et al.* (2012); Zhu *et al.* (2013); Giles et al. (2015c).

79. Gnathal plates mesial to and/or above (or below) jaw cartilage: absent (0); present (1). Zhu *et al*. (2016).

80. Maxilla and premaxilla *sensu stricto* (upper gnathal plates lateral to jaw cartilage without palatal lamina): absent (0); present (1). [Zhu *et al*. (2016).

81. Dentary bone encloses mandibular sensory canal: absent (0); present (1). Gardiner (1984); see also Zhu *et al.* (2009, 2013).

82. Infradentary foramen and groove, series: absent (0); present (1). Zhu *et al*. (2010): note discussion of the lower jaw attributed, to *Meemannia*.

83. Tooth-bearing median rostral: absent (0); present (1). Zhu *et al.* (2009, 2013).

84. Median dermal bone of palate (parasphenoid): absent (0); present (1). Gardiner (1984); Brazeau (2009); Davis *et al.* (2012); Zhu *et al.* (2013).

85. Denticulated field of parasphenoid: without spiracular groove (0); with spiracular groove (1). Friedman (2007); Zhu *et al.* (2009, 2013).

86. Denticle field of parasphenoid with multifid anterior margin: absent (0); present (1). Friedman (2007); Zhu *et al.* (2009, 2013); Lu *et al*. (2016).

Mandibular arch

87. Large otic process of the palatoquadrate: absent (0); present (1). Coates & Sequeira (2001a); Davis (2002); Brazeau (2009); Zhu *et al.* (2009, 2013).

88. Oblique ridge or groove along medial face of palatoquadrate: absent (0); present (1). Brazeau (2009); Davis *et al.* (2012); Zhu *et al.* (2013); Lu et al. (2016). Likely more widespread than previously thought: in *Ferromirum* the ridge matches the position of that in *Acanthodes*, although the cross-sectional profile is more rounded. Unable to score in *Ozarcus* because the more dorsal portion of the otic process is absent.

89. Fenestration of palatoquadrate at basipterygoid articulation: absent (0); present (1). Brazeau (2009); Davis *et al.* (2012); Zhu *et al.* (2013); Lu et al. (2016).

90. Perforate or fenestrate anterodorsal (metapterygoid) portion of palatoquadrate: absent (0); present (1). Davis (2002); Brazeau (2009); Davis *et al.* (2012); Zhu *et al.* (2013).

91. Articulation surface of the palatoquadrate with the postorbital process directed anteriorly (0); laterally (1); dorsally (2).

92. Palatoquadrate fused to the neurocranium: absent (0); present (1).

93. Pronounced dorsal process on Meckelian bone or cartilage: absent (0); present (1). Hanke & Wilson (2004); Brazeau (2009); Davis *et al.* (2012); Zhu *et al.* (2013); Burrow *et al.* (2016).

94. Mandibular knob or mesial process: absent (0); present (1). Brazeau (2009); Davis *et al.* (2012); Zhu *et al.* (2013); Burrow *et al.* (2016).

95. Jaw articulation located on rearmost extremity of mandible: absent (0); present (1). Davis *et al.* (2012); Zhu *et al.* (2013).

96. Meckel's cartilage with flange or shelf projecting posteriorly from the lateral cotylus (glenoid): absent (0); present (1). *Tristychius* (Coates *et al*. 2019) and *Gogoselachus* (Long *et al*. 2015, figs 1C and 2A, G) exhibit the same derived condition in which this retroarticular flange is an uninterrupted, smooth extension of the lateral wall of the mandible.

97. Dental trough adjacent to oral rim on Meckel's cartilage and palatoquadrate: absent (0); present (1).

98. Dental trough divided, scalloped tooth-bearing margin on Meckel's cartilage and palatoquadrate: absent (0); present (1).

99. Mandibular symphysis fused: absent (0); present (1).

Neurocranium

100. Internasal vacuities: absent (0); present (1). Lu *et al.* (2016).

101. Precerebral fontanelle: absent or minimal (0); present and large (1). Schaeffer (1981); Lund & Grogan (1997); Coates & Sequeira (1998, 2001a, b); Maisey (2001a); Brazeau (2009); Pradel *et al.* (2011) Davis *et al.* (2012); Zhu *et al.* (2013); Brazeau & Friedman (2014).

102. Space for forebrain and (at least) proximal portion of olfactory tracts narrow and elongate, extending between orbits: absent (0); present (1).

103. Rostral bar: absent (0); present (1). Maisey (1985).

104. Internasal groove absent (0); present (1).

105. Orbitonasal lamina expanded: absent (0); present (1). Patterson (1965).

106. Elongate, tooth-bearing, pre-nasal ethmo-rostral region: absent (0); present (1).

107. Palatobasal (or orbital) articulation posterior to the optic foramen (0); anterior to the optic foramen, grooved, and overlapped by process or flange of palatoquadrate (1); anterior to optic foramen, smooth, and overlaps or flanks articular surface on palatoquadrate (2). Pradel *et al.* (2011); Coates *et al.* (2017); Maisey (2005).

108. Trochlear nerve foramen anterior to optic nerve foramen: absent (0); present (1).

109. Supraorbital shelf broad with convex lateral margin: absent (0); present (1). Brazeau (2009); Davis *et al.* (2012); Zhu *et al.* (2013).

110. Interorbital space broad (0); narrow (1). Brazeau (2009); Davis *et al.* (2012); Zhu *et al.* (2013); Coates et al. (2017).1

111. Optic pedicel: absent (0); present (1). Dupret (2014); Zhu et al. (2009, 2013); Coates et al. (2017).

112. Large prootic foramen separated from optic fenestra by antotic pillar bearing optic pedicel: absent (0); present (1). Adapted from Maisey et al. (2019); likely synapomorphy of pucapampellids.

113. Ophthalmic foramen in anterodorsal extremity of orbit communicates with enclosed cranial space: absent (0); present (1).

114. Extended prehypophysial portion of sphenoid: absent (0); present (1). Brazeau (2009); Davis *et al.* (2012); Zhu *et al.* (2013).

115. Canal for efferent pseudobranchial artery within basicranial cartilage: absent (0); present (1). Brazeau (2009); Davis *et al.* (2012); Zhu *et al.* (2013).

116. Entrance of internal carotids: through separate openings flanking the hypophyseal opening or recess (0); through a common opening at the central midline of the basicranium (1). Schaeffer (1981); Coates & Sequeira (1998); Brazeau (2009); Davis *et al.* (2012); Zhu *et al.* (2013).

117. Internal carotids: entering single or paired openings in the basicranium from a posterolateral angle (0); entering basicranial opening(s) head-on from an extreme, lateral angle (1); absent (2).

118. Ascending basisphenoid pillar pierced by common internal carotid: absent (0); present (1). Miles (1973b); Brazeau (2009); Friedman & Brazeau (2010); Davis *et al.* (2012); Zhu *et al.* (2013).

119. Spiracular groove on basicranial surface: absent (0); present (1). Davis *et al.* (2012); Zhu *et al.* (2013).

120. Spiracular groove on lateral or transverse wall of jugular canal: absent (0); present (1). Davis *et al.* (2012); Zhu *et al.* (2013).

121. Spiracular groove open (0); enclosed by spiracular bar or canal (1). Lu *et al*. (2016); (Patterson 1982).

122. Orbit larger than otic capsule: absent (0); present (1). Lund & Grogan (1997); Coates *et al*. (2017).

123. Postorbital process and arcade: absent (0); present (1). Pradel *et al.* (2011).

124. Postorbital process and arcade short and deep - width not more than maximum braincase width (excluding arcade) (0); process and arcade wide - width exceeds maximum width of braincase, and anteroposteriorly narrow (1); process and arcade massive (2); arcade forms postorbital pillar (3).

125. Postorbital process downturned, with anhedral angle relative to basicranium: absent (0); present (1). (Maisey 2011).

126. Jugular canal diameter small (0); large (1); canal absent (2). Pradel *et al.* (2011).

127. Canal, likely for trigeminal nerve (V) mandibular ramus, passes through the postorbital process from proximal dorsal entry to distal and ventral exit: absent (0); present (1).

128. Postorbital process expanded anteroposteriorly: absent (0); present (1).

129. Postorbital process articulates with palatoquadrate: absent (0); present (1). Schaeffer (1981); Coates & Sequeira (1998); Maisey (2001a); Pradel *et al.* (2011); Davis *et al.* (2012); Zhu *et al.* (2013).

130. Trigemino-facial recess: absent (0); present (1). Goodrich (1930); Gardiner (1984); Pradel (2010); Pradel *et al*. (2011); Davis *et al.* (2012).

131. Jugular canal long, extends throughout most of otic capsule wall posterior to the postorbital process (0); short and/or groove present on exterior of otic wall (1); absent, path of jugular removed from otic wall (2). Brazeau (2009); Davis *et al.* (2012); Zhu *et al.* (2013); Giles *et al.* (2015c); Coates *et al.* (2017).

132. C-bout notch separates postorbital process from supraotic shelf: absent (0); present (1).

133. Hyoid ramus of facial nerve (N. VII) exits through posterior jugular opening: absent (0); present (1). Friedman (2007); Brazeau (2009); Friedman & Brazeau (2010); Davis *et al.* (2012); Zhu *et al.* (2013).

134. Periotic process: absent (0); present (1). Maisey (2007); Coates *et al*. (2017).

135. Relative position of jugular groove and hyomandibular articulation: hyomandibula dorsal or same level (i.e. on bridge) (0); jugular vein passing dorsal or lateral to hyomandibula (1). Brazeau & de Winter (2015).

136. Transverse otic process: absent (0); present (1). Lu *et al*. (2016); Giles *et al*. (2016); Giles *et al.* (2015).

137. Craniospinal process: absent (0); present (1). Giles *et al.* (2015); Lu *et al*. (2016).

138. **Lateral otic process: absent (0); present (1).** Schaeffer (1981); Coates & Sequeira (1998); Brazeau (2009); Davis *et al.* (2012); Zhu *et al.* (2013). Although identified present in *Gutturensis* (Sequeira & Coates 2000, Text-Fig. 3), this is not the prominent lateral otic process of *Xenacanths* or *Tamiobatis*.

139. **Hyomandibula articulates with neurocranium beneath otic shelf: absent (0); present (1).**

140. **Sub-otic occipital fossa: absent (0); present (1).**

141. **Postotic process: absent (0); present (1)** Pradel *et al.* (2011).

142. **Otic capsule extends posterolaterally relative to occipital arch: absent (0); present (1)** Maisey (1985).

143. **Otic capsules: widely separated (0); approaching dorsal midline (1).**

144. **Otic capsules project anteriorly between postorbital processes: absent (0); present (1)** (Maisey 1983).

145. **Endocranial roof anterior to otic capsules domelike, smoothly convex dorsally and anteriorly: absent (0); present (1).**

146. **Roof of skeletal cavity for cerebellum and mesencephalon significantly higher than dorsal-most level of semicircular canals: absent (0); present (1).**

147. **Roof of the endocranial space for telencephalon and olfactory tracts offset ventrally relative to level of mesencephalon: absent (0); present (1).** Coates *et al.* (2017).

148. **Labyrinth cavity separated from the main neurocranial cavity by a cartilaginous or ossified capsular wall (0); skeletal medial capsular wall absent (1).** Pradel *et al.* (2011); Davis *et al.* (2012); Zhu *et al.* (2013).

149. **Double octaval nerve foramena in chondrified mesial wall of otic capsule: absent (0); present (1).**

150. **External (horizontal) semicircular canal joins the vestibular region dorsal to posterior ampulla (0); joins level with posterior ampulla (1).** Davis *et al.* (2012); Zhu *et al.* (2013).

151. **Angle of external semicircular canal: in lateral view, straight line projected through canal intersects anterior ampulla, external ampullae, and base of foramen magnum: absent (0); present (1)** Maisey (2007).

152. **Left and right external semicircular canals approach or meet the posterodorsal midline of the hindbrain roof: absent (0); present (1).**

153. **Preampullary portion of posterior semicircular canal absent (0); present (1).**

154. **Crus commune connecting anterior and posterior semicircular canals: present (0); absent (1).**

155. **Sinus superior: absent or indistinguishable from union of anterior and posterior canals with saccular chamber (0); present, elongate and nearly vertical (1).** Davis *et al.* (2012); Zhu *et al.* (2013).

156. **Lateral cranial canal: absent (0); present (1).** Zhu *et al.* (2009, 2013); Lu *et al.* (2016). Gardiner (1984); Coates (1998).

157. **Endolymphatic ducts: posteriodorsally angled tubes (0); tubes oriented vertically through endolymphatic fossa/posterior dorsal fontanelle (1).** Schaeffer (1981); Coates & Sequeira (1998, 2001); Davis (2002); Brazeau (2009); Davis *et al.* (2012); Zhu *et al.* (2013).

158. **Posterior dorsal fontanelle connected to persistent otico-occipital fissure (0); posterior tectum separates fontanelle from fissure (1).** Schaeffer (1981); Coates & Sequeira (1998); Pradel *et al.* (2011).

159. **Subcircular endolymphatic foramen: absent (0); present (1).** Pradel *et al.* (2015); Maisey & Lane (2010).

160. External opening for endolymphatic ducts anterior to crus commune: absent (0); present (1). Coates *et al.* (2017).

161. Supraotic shelf broad: absent (0); present (1).

162. Dorsal otic ridge: absent (0); present (1). Coates & Sequeira (1998, 2001); Maisey (2001); Davis (2002); Davis *et al.* (2012); Zhu *et al.* (2013); Brazeau & Friedman (2014). As discussed by Brazeau and Friedman, the utility of this likely chondrichthyan synapomorphy is challenged by the difficulty of coding for presence in taxa with firmly attached dermatocranial bones.

163. Dorsal otic ridge forms a crest posteriorly: absent (0); present (1). Coates & Sequeira (1998, 2001); Pradel *et al.* (2011).

164. Endolymphatic fossa: absent (0); present (1). Pradel *et al.* (2011). The endolymphatic fossa identified in *Gutturensis* (Sequeira & Coates 2000, Text-Fig. 3) is likely an eroded surface revealing the dorsally ascending endolymphatic ducts.

165. Endolymphatic fossa elongate (slot-shaped), dividing dorsal otic ridge along midline: absent (0); present (1). Coates *et al.* (2017).

166. Perilymphatic fenestra within the endolymphatic fossa: absent (0); present (1). Pradel *et al.* (2011); Coates *et al.* (2017).

167. Ventral cranial fissure: absent (0); present (1). Janvier (1996); Coates & Sequeira (2001); Maisey (2001); Davis (2002); Brazeau (2009); Pradel *et al.* (2011); Davis *et al.* (2012); Zhu *et al.* (2013).

168. Endoskeletal intracranial joint: absent (0); present (1). Janvier (1996, and references therein); Davis *et al.* (2012); Zhu *et al.* (2013).

169. Metotic (otic-occipital) fissure: absent (0); present (1). Schaeffer (1981); Janvier (1996); Coates & Sequeira (1998); Maisey (2001); Davis (2002); Brazeau (2009); Pradel *et al.* (2011); Davis *et al.* (2012); Zhu *et al.* (2013).

170. Vestibular fontanelle: absent (0); present (1). Brazeau (2009); Friedman & Brazeau (2010). Davis *et al.* (2012); Zhu *et al.* (2013).

171. Hypotic lamina: absent (0); present (1). Schaeffer (1981); Maisey (1984, 2001); Brazeau (2009); Pradel *et al.* (2011, 2013); Davis *et al.* (2012); Zhu *et al.* (2013). Note recent discussions in Coates *et al.* (2017) and Maisey *et al.* (2019).

172. Glossopharyngeal nerve path: directed laterally, across floor of the saccular chamber and exits via foramen in side wall of the otic capsule (0); directed posteriorly, and exits through metotic fissure or foramen in posteroventral wall of otic capsule (1); exits laterally through a canal contained ventrally (floored) by the hypotic lamina (2); exits through a foramen anterior to the posterior ampulla (3). Coates et al. (2017), adapted from Schaeffer (1981); Coates & Sequeira (1998, 2001); Brazeau (2009): Davis *et al.* (2012); Zhu *et al.* (2013); Pradel *et al.* (2011, 2013).

173. Glossopharyngeal and vagus nerves share common exit from neurocranium: absent (0); present (1).

174. Basicranial morphology: platybasic (0); tropibasic (1). Brazeau (2009); Pradel *et al.* (2011); Davis *et al.* (2012); Zhu *et al.* (2013); Maisey (2007); Pradel *et al* (2011).

175. Channel for dorsal aorta and/or lateral dorsal aortae passes through basicranium (0): external to basicranium (1). Schaeffer (1981); Coates & Sequeira (1998); Brazeau (2009); Pradel *et al.* (2011); Brazeau & Friedman (2014); Coates et al. (2017).

176. Dorsal aorta divides into lateral dorsal aortae posterior to occipital level (0); anterior to level of the occiput (1). Pradel *et al.* (2011); Giles *et al.* (2015); Coates et al. (2017).

177. Posterior openings of lateral aortic canals positioned lateral to occipital cotylus: absent (0); present (1). Adapted from Maisey et al. (2019); likely synapomorphy of pucapampellids.

178. Ventral portion of occipital arch wedged between rear of otic capsules: absent (0); present (1). Schaeffer (1981); Coates & Sequeira (1998); Maisey (2001a); Brazeau (2009); Pradel *et al.* (2011); Davis *et al.* (2012); Coates *et al.* (2017).

179. Dorsal portion of occipital arch wedged between otic capsules: absent (0); present (1). Schaeffer (1981); Coates & Sequeira (1998); Maisey (2001a); Brazeau (2009); Pradel *et al.* (2011); Davis *et al.* (2012); Coates *et al.* (2017).

180. Occipital crest anteroposteriorly elongate, and extends from the roof of the posterior tectum: absent (0); present (1).

Axial and appendicular skeleton

181. Calcified vertebral centra: absent (0); present (1). Maisey (1985): biconcave calcified disc centra surrounding constricted notochord, suggested synapomorphy of crown elasmobranchs (Coates *et al.* 2017).

182. Chordacentra: absent (0); present (1). Stahl (1999); Coates and Sequeira (2001); Coates *et al*. (2017).

183. Chordacentra polyspondylous and consist of narrow closely packed rings: absent (0); present (1). Patterson (1965); Coates *et al*. (2017).

184. Synarcual: absent (0); present (1). Stahl (1999); Brazeau (2009); Davis *et al.* (2012); Zhu *et al.* (2013); Coates *et al.* (2017).

185. Macromeric dermal pectoral girdle (0); micromeric or lacking dermal skeleton entirely (1). Brazeau (2009); Davis *et al.* (2012); Zhu *et al.* (2013).

186. Macromeric dermal pectoral girdle composition: ventral and dorsal components (0); ventral components only (1). Brazeau (2009); Davis *et al.* (2012); Zhu *et al.* (2013).

187. Macromeric pectoral dermal skeleton forms complete ring around the trunk: present (0); absent (1). Goujet & Young (2004); Brazeau (2009); Davis *et al.* (2012); Zhu *et al.* (2013).

188. Median dorsal plate: absent (0); present (1). Brazeau (2009); Davis *et al.* (2012); Zhu *et al.* (2013).

189. Scapular process (dorsal) of shoulder endoskeleton: absent (0); present (1). Coates & Sequeira (2001a); Zhu & Schultze (2001); Davis (2002); Brazeau (2009); Davis *et al.* (2012); Zhu *et al.* (2013); Brazeau & Friedman (2014).

190. Ventral margin of separate scapular ossification: horizontal (0); deeply angled (1). Hanke & Wilson (2004); Brazeau (2009); Davis *et al.* (2012); Zhu *et al.* (2013).

191. Cross sectional shape of scapular process: flattened or strongly ovate (0); subcircular (1). Davis (2002); Brazeau (2009); Davis *et al.* (2012); Zhu *et al.* (2013); Burrow *et al.* (2016).

192. Flange on trailing edge of scapulocoracoid: absent (0); present (1). Davis (2002); Brazeau (2009); Davis *et al.* (2012); Zhu *et al.* (2013); Burrow *et al.* (2016).

193. Scapular process with posterodorsal process. Absent (0); present (1). Coates & Sequeira (2001a); Davis *et al.* (2012); Zhu *et al.* (2013).

194. Mineralisation of internal surface of scapular process: mineralised all around (0); un-mineralised on internal face forming a hemicylindrical cross-section. Brazeau (2009); Davis *et al.* (2012); Zhu *et al.* (2013); Burrow *et al.* (2016).

195. **Coracoid process: absent (0); present (1).** Brazeau (2009); Davis *et al.* (2012); Zhu *et al.* (2013).

196. **Procoracoid mineralisation: absent (0); present (1).** Davis (2002); Hanke & Wilson (2004): Brazeau (2009).

197. **Fin base articulation on scapulocoracoid: stenobasal, deeper than wide (0); eurybasal, wider than deep (1).** Lu et al. (2016).

198. **Pectoral fin articulation monobasal (0); dibasal (1); three or more basals (2).**

199. **Metapterygium pectinate subtriangular plate or bar supporting numerous (six or more) radials along distal edge: absent (0); present (1).**

200. **Metapterygial whip absent (0); present (1).** Coates *et al*. (2017).

201. **Biserial pectoral fin endoskeleton: absent (0); present (1).** Lu *et al*. (2016).

202. **Propterygium perforated: absent (0); present (1).** Rosen *et al.* (1981); Patterson (1982); Davis *et al.* (2012); Zhu *et al.* (2013).

203. **Pelvic girdle with fused puboischiadic bar: absent (0); present (1).** Maisey (1984); Coates & Sequeira (2001a); Coates *et al*. (2017).

204. **Mixipterygial/mixopterygial claspers: absent (0), present (1).** Brazeau & Friedman (2014); Coates & Sequeira (2001a,b); Compagno (1999); Liem & Summers (1999); Long *et al*. (2015); Trinajstic *et al*. (2014).

205. **Pre-pelvic clasper or tenaculum: absent (0); present (1).** Patterson (1965); Coates et al. (2017).

206. **Number of dorsal fins, if present: one (0); two (1); one, extending from pectoral to anal fin level (2).** Coates & Sequeira (2001a); Brazeau (2009); Davis *et al.* (2012); Zhu *et al.* (2013).

207. **Brush complex of bilaterally distributed calcified tubes flanking or embedded in calcified cartilage core: absent (0); present (1).**

208. **Posterior or pelvic-level dorsal fin with calcified base plate: absent (0); present (1).** Coates & Sequeira (2001a, b).

209. **Posterior dorsal fin with delta-shaped cartilage: absent (0); present (1).** Coates & Sequeira (2001a, b).

210. **Posterior or pelvic-level dorsal fin shape, base approximately as broad as tall and not broader than other median fins (0); base much longer than fin height, substantially longer than other median fins (1).** Brazeau & deWinter (2015); Lu *et al*. (2017).

211. **Anal fin: absent (0); present (1).** Coates & Sequeira (2001); Brazeau (2009); Davis *et al.* (2012); Zhu *et al.* (2013).

212. **Anal fin base narrow, posteriormost proximal segments radials broad: absent (0); present (1).**

213. **Caudal radials restricted to axial lobe (0); extend beyond level of body wall and deep into hypochordal lobe (1).** Davis *et al.* (2012); Zhu *et al.* (2013).

214. **Caudal neural and/or supraneural spines or radials short (0); long, expanded, and supporting high aspect-ratio (lunate) tail with notochord extending to posterodorsal extremity (1); notochord terminates pre-caudal extremity, neural and heamal radial lengths near symmetrical and support epichordal and hypochordal lobes respectively (2).**

Spines: fins, cranial and elsewhere

215. Dorsal fin spine or spines: absent (0); present (1). Zhu *et al*. (2001); Zhu & Yu (2002); Friedman (2007); Brazeau (2009); Davis *et al.* (2012); Zhu *et al.* (2013); Lu *et al*. (2016).

216. Dorsal fin spine at anterior (pectoral level) location only: absent (0); present (1).

217. Dorsal fin spine cross section: horseshoe shaped (0); flat sided with rectangular profile (1); subcircular (2). Hampe (2002); Brazeau & de Winter (2015).

218. Dorsal fin spine apex curved posteriorly: absent (0); present (1). Present in *Ferromirum* and *Cladoselache*.

219. Anterior dorsal fin spine leading edge concave in lateral view: absent (0); present (1).

220. Anal fin spine: absent (0); present (1). Maisey (1986); Davis (2002); Brazeau (2009).

221. Pectoral fin spines: absent (0); present (1). Davis (2002); Brazeau (2009); Davis *et al.* (2012); Zhu *et al.* (2013).

222. Pectoral fin spine with denticles along posterior surface: absent (0); present (1). Burrow *et al*. (2016).

223. Prepectoral fin spines: absent (0); present (1). Davis (2002); Hanke & Wilson (2004); Brazeau (2009); Davis *et al.* (2012); Zhu *et al.* (2013). Present in *Doliodus* (Maisey *et a*l. 2017); see also Dearden *et al*. (2019).

224. Admedian pectoral spines absent (0); present (1). Burrow *et al*. (2016).

225. Median fin spine insertion: shallow, not greatly deeper than dermal bones/ scales (0); deep (1). Davis (2002); Hanke & Wilson (2004); Brazeau (2009). Davis *et al.* (2012); Zhu *et al.* (2013).

226. Intermediate (pre-pelvic) fin spines: absent (0); present (1). Davis (2002); Hanke & Wilson (2004); Brazeau (2009); Davis *et al.* (2012); Zhu *et al.* (2013).

227. Fin spines with ridges: absent (0); present (1). Davis (2002); Brazeau (2009); Davis *et al.* (2012); Zhu *et al.* (2013).

228. Fin spines with nodes: absent (0); present (1). Davis (2002); Hanke & Wilson (2004); Brazeau (2009). Davis *et al.* (2012); Zhu *et al.* (2013).

229. Fin spines (dorsal) with rows of large denticles: absent (0); on posterior surface (1); on lateral surface (2). Maisey (1989b); Davis *et al.* (2012); Zhu *et al.* (2013); Brazeau & Friedman (2014).

230. Cephalic spines: absent (0); present (1). Maisey (1989); Coates *et al.* (2017).

Synapomorphy lists (deltran optimisation)

Asterisk indicates synapomorphy with CI 1.0.

Total group Chondrichthyes

32 Absence of cheek plates
40 Absence of dermohyal
43 Absence of branchiostegal series
48* Absence of gular plates
67 Generative tooth sets
72 Generative tooth sets distributed along jaw margin
78 Absence of dermal plates along biting margin
189* Scapular process
228 Prepelvic (intermediate) fin spines

Acanthodian rump group

Conventional Chondrichthyes?

Crown group Chondrichthyes

Total group Holocephali

Ferromirum and Gutturensis

Total group Elasmobranchii

Supplementary References

Adrain, J. M., & Wilson, M. V. H. Early Devonian cephalaspids (Vertebrata: Osteostraci: Cornuata) from the southern Mackenzie Mountains. *J. Vertebr. Paleontol.* **14**, 301–319 (1994).

Andrews, S. M., & Westoll, T. S. The postcranial skeleton of *Eusthenopteron foordi* Whiteaves. *Trans. R. Soc. Edinb.* **68**, 207-329 (1970).

Andrews, S. M., Long, J. A., Ahlberg, P. E., Barwick, R. E. & Campbell, K. S. W. The structure of the sarcopterygian *Onychodus jandemarri n. sp.* From Gogo, Western Australia: with a functional interpretation of the skeleton. *Trans. R. Soc. Edinb. Earth Sci.* **96**, 197–307 (2006).

Arratia, G. & Cloutier, R. in *Devonian Fishes and Plants of Miguasha, Quebec, Canada* (eds. Schultze, H.-P. & Cloutier, R.) 165-197 (Verlag Dr. Friedrich Pfeil, Munich, 1996).

Basden, A. M. & Young, G. C. A primitive actinopterygian neurocranium from the Early Devonian of Southeastern Australia. *J. Vertebr. Paleontol.* **21**, 754-766 (2001).

Basden, A. M., Young, G. C., Coates, M. I. & Ritchie, A. The most primitive osteichthyan braincase? *Nature* **403**, 185–188 (2000).

Bernacsek, G.M., & Dineley, D.L. New acanthodians from the Delorme Formation (Lower Devonian) of N.W.T., Canada. *Paleontographica Abt.A* **158,** 1-25 (1977).

Bendix-Almgreen, S. E. The paired fins and shoulder girdle in *Cladoselache*, their morphology and phyletic significance. *Colloques Internationaux du Centre National de la Recherche Scientifique* **218**, 111-123 (1975).

Beznosov, P. A redescription of the Early Carboniferous acanthodian *Acanthodes lopatini* Rohon, 1889. *Acta Zoologica* **90** (supplement 1), 183-193 (2009).

Botella, H., Blom, H., Dorka, M., Ahlberg, P. E. & Janvier, P. Jaws and teeth of the earliest bony fishes. *Nature* **448**, 583-586 (2007).

Brazeau, M. D. The braincase and jaws of a Devonian 'acanthodian' and modern gnathostome origins. *Nature* **457**, 305-308 (2009).

Brazeau, M. D. A revision of the anatomy of the early Devonian jawed vertebrate *Ptomacanthus anglicus* Miles. *Palaeontology* **55**, 355-367 (2012).

Brazeau, M. D. & Friedman, M. The characters of Palaeozoic jawed vertebrates. *Zoological Journal of the Linnean Society*, **170**, 779-821 (2014).

Brazeau, M. D. & de Winter, V. The hyoid arch and braincase anatomy of *Acanthodes* support chondrichthyan affinity of 'acanthodians'. *Proc. R. Soc.* B **282**: 20152210 (2015).

Brazeau, M. D., Friedman, M., Jerve, A., & Atwood, R. C. A three-dimensional placoderm (stem-group gnathostome) pharyngeal skeleton and its implications for primitive gnathostome pharyngeal architecture. *Journal of Morphology*. DOI: 10.1002/jmor.20706 (2017).

Bremer, K. The limits of amino-acid sequence data in angiosperm phylogenetic reconstruction. *Evolution* **42,** 795-803 (1988).

Burrow, C. J. A new lophosteiform (Osteichthyes) from the Lower Devonian of Australia. *Geobios M. S.* **19**, 327-333 (1995).

Burrow, C. J. A partial articulated acanthodian from the Silurian of New Brunswick, Canada. *Can. J. Earth Sci* **48**, 1329-1341 (2011).

Burrow, C. J., den Blaauwen, J., Newman, M. & Davidson, R. The diplacanthid fishes (Acanthodii, Diplacanthiformes, Diplacanthidae) from the Middle Devonian of Scotland. *Palaeontologica Electronica* **19**, 1-83 (2016).

Burrow, C. J., Newman, M. J., Davidson, R. G., & den Blaauwen, J. L. Sclerotic plates or

circumorbital bones in early jawed fishes? *Palaeontology* **54**, 207- 214 (2011).

Burrow, C. J. & Rudkin, D. Oldest near-complete acanthodian: the first vertebrate from the Silurian Bertie formation konservat-Lagerstätte, Ontario. *PLoS ONE* **9**(8), DOI:10.1371/journal.pone.0104171 (2014).

Burrow, C. J., Trinajstic, K. & Long, J. First acanthodian from the Upper Devonian (Frasnian) Gogo Formation, Western Australia. *Historical Biology* 24, DOI:10.1080/08912963.2012.660150 (2012).

Burrow, C. J. & Turner, S. Devonian placoderm scales from Australia. *Journal of Vertebrate Paleontology* **18**, 677-695 (1998).

Burrow, C. J. & Turner, S. A review of placoderm scales, and their significance in placoderm phylogeny. *Journal of Vertebrate Paleontology* **19**, 204-219 (1999).

Burrow, C.J., & Turner, S. Reassessment of "*Protodus*" *scoticus* from the Early Devonian of Scotland. In *Morphology, Phylogeny and Paleobiogeography of Fossil Fishes*. (eds Elliot, D.K., Maisey, J.G., Yu, X. & Miao, D.) 123–144 (Freidrich Pfeil, 2010).

Burrow, C. J. & Turner, S. Scale structure of the putative chondrichthyan *Gladbachus adentatus* Heidtke & Krätschmer, 2001 from the Middle Devonian Rheinisches Schiefergebirge, Germany. *Historical Biology* **25**, 385-390 (2013).

Burrow, C. J., Turner, S. & Heidtke, U. H. J. The squamation of *Gladbachus adentatus* Heidtke & Kratschmer, 2001. *UNESCO-IUGCP 491: The Gross Symposium 2 Advances in Palaeoichthyology, Riga, Latvia, September* 7-14, 2003, p 16-17 (2003).

Carr, R. K., Johanson, Z. & Ritchie, A. The phyllolepid placoderm *Cowralepis mclachlani*: insights into the evolution of feeding mechanisms in jawed vertebrates. *J. Morph* **270**, 775-804 (2009).

Chang, M. M. *The braincase of* Youngolepis*, a Lower Devonian crossopterygian from Yunnan, south-western China*. Doctoral dissertation, Department of Geology, Stockholm University (1982).

Chang, M.-M. Head exoskeleton and shoulder girdle of *Youngolepis*. In *Early Vertebrates and Related Problems of Evolutionary Biology*, M.-M. Chang, Y.-H. Liu and G.-R. Zhang, eds. (Beijing: Science Press), pp. 355–378 (1991).

Chang, M.-M. Synapomorphies and scenarios - more characters of *Youngolepis* betraying its affinity to the Dipnoi. In *Recent Advances in the Origin and Early Radiation of Vertebrates*, G. Arratia, M. V. H. Wilson and R. Cloutier, eds. (München: Verlag Dr. Friedrich Pfeil), pp. 665–686 (2004).

Chang, M.-M. & Yu, X.-B. A new crossopterygian, *Youngolepis praecursor*, gen. et sp. nov., from Lower Devonian of E. Yunnan, China. *Sci. Sin.* **24**, 89–97 (1981).

Chang, M.-M. & Yu, X.-B. Reexamination of the relationship of Middle Devonian osteolepids - fossil characters and their interpretations. *American Museum Novitates* **3189**, 1-20 (1997).

Chang, M.-M. & Zhu, M. A new Middle Devonian osteolepidid from Qujing, Yunnan. *Memoirs of the Association of Australasian Palaeontologists* **15**, 183-198 (1993).

Choo, B. Revision of the actinopterygian genus *Mimipiscis* (= *Mimia*) from the Upper Devonian Gogo Formation of Western Australia and the interrelationships of early Actinopterygii. *Earth and Environmental Science Transactions of the Royal Society of Edinburgh*, **102**: 77-104 (2011)

Clement, G. Nouvelles données anatomiques et morphologie générale des "Porolepididae" (Dipnomorpha, Sarcopterygii). *Revue de Paléobiologie, Genève* **Volume spéciale 9**, 193-211 (2004).

Cloutier, R., & Ahlberg, P. E. in *Interrelationships of Fishes* (eds Stiassny, M. L. J., Parenti, L. R., & Johnson, G. D.) 445–479. (Academic, 1996).

Coates, M. I. The origin of vertebrate limbs. *Development* Supplement, 169-180 (1994).

Coates, M. I. Actinopterygians from the Namurian of Bearsden, Scotland, with comments on the early evolution of actinopterygian neurocrania. *Zool. J. Linn. Soc.* **122**, 27-59. (1998).

Coates, M. *Gladbachus adentatus* Heidtke & Kratschmer; is it a chondrichthyan, and if so, where does it fit in phylogeny? *Paleobios* **25**, 31-32 (2005).

Coates, M. I., Finarelli, J. A., Sansom, I. J., Andreev, P. S., Criswell, K. E., Tietjen, K., Rivers, M. L., & La Riviere, P. J. An early chondrichthyan and the evolutionary assembly of a shark body plan. *Proc. R. Soc. B.* 20172418 (2018).

Coates, M. I. & Friedman, M. in *Morphology, Phylogeny and Paleobiogeography of Fossil Fishes*. (eds Elliot, D.K., Maisey, J.G., Yu, X. & Miao, D.) 389–416 (Freidrich Pfeil, 2010).

Coates, M. I. & Gess, R. W. A new reconstruction of *Onychoselache Traquairi*, comments on early chondrichthyan pectoral girdles and hybodontiform phylogeny. *Palaeontology* **50**, 1421-1446 (2007).

Coates, M. I., Gess, R. W., Finarelli, J. A., Criswell, K. E. & Tietjen, K. A symmoriiform chondrichthyan braincase and the origin of chimaeroid fishes. *Nature* **541**, 209-211 (2017).

Coates, M. I., Sansom, I. J. & Smith, M. M. Spines and tissues of ancient sharks. *Nature* **396**, 729-730 (1998).

Coates, M. I. & Sequeira, S. E. K. The braincase of a primitive shark. *Trans. R. Soc. Edinb. (Earth Sci.)* **89**, 63–85 (1998).

Coates, M. I. & Sequeira, S. E. K. A new stethacanthid chondrichthyan from the Lower Carboniferous of Bearsden, Scotland. *J. Vertebr. Paleontol.* **21**, 754–766 (2001a).

Coates, M. I. & Sequeira, S. E. K. in *Major Events in Early Vertebrate Evolution* (ed. Ahlberg, P. E.) 241–262 (Taylor and Francis, 2001b).

Coates, M. I. & Tietjen, K. The neurocranium of the Lower Carboniferous shark *Tristychius arcuatus* (Agassiz, 1837). *Earth and Environmental Science Transactions of the Royal Society of Edinburgh* (in press).

Coates, M. I., Tietjen, K., Olsen, A. M. & Finarelli, J. A. High-performance suction feeding in an early elasmobranch. *Sci. Adv.* **5,** eaax2742 (2019).

Coates, M. I., Tietjen, K., Johanson, Z., Friedman, M., Sang, S. The cranium of *Helodus simplex* (Agassiz, 1838) revised. In: John Maisey symposium, Ichthyol explanation freshwaters (eds. Denton, J., Pradel, A. & Janvier, P.). (Verlag Pfeil Forthcoming 2020).

Cole, F. J. On the cranial nerves of *Chimaera monstrosa* (Linn.); with a discussion of the lateral line system and of the morphology of the chorda tympani. *Trans. R. Soc. Edinb.* **38**, 631-680 (1896).

Compagno, L. J. V. in *Sharks, Skates, and Rays, the Biology of Elasmobranch Fishes* (ed. Hamlett, W. C.) 69-92 (Johns Hopkins University, 1999).

Creanza, N., Schwarz, J. S. & Cohen, J. E. Intraseasonal Dynamics and Dominant Sequences in H3N2 Influenza. *PLoS ONE* **5**, (2010), doi: 10.1371/journal.pone.0008544

Daniel, J. F. *The Elasmobranch Fishes* (Univ. California Press, Berkeley, 1922).

Davis, J. C. *Statistics and Data Analysis in Geology* (Wiley, New York, 1986).

Davis, S. P. Comparative anatomy and relationships of the acanthodian fishes. *Unpublished PhD thesis, Unv. London*, 1–318. (2002).

Davis, S. P., Finarelli, J. A., & Coates, M. I. *Acanthodes* and shark-like conditions in the last common ancestor of modern gnathostomes. *Nature* **486**, 247-250 (2012).

DeBeer, G. R. *The Development of the Vertebrate Skull* (Univ. Chicago, 1937).

De Beer, G. R. & Moy-Thomas, J. A. On the skull of Holocephali. *Philos. Trans. R. Soc. London B. Biol. Sci.* **224**: 287-312 (1935).

Dean, M. N. & Summers, A. P. Cartilage in the skeleton of cartilaginous fishes. *Zoology* **109**,164–168 (2006).

Dean, M. N., Mull, C. G., Gorb, S. N. & Summers, A. P. Ontogeny of the tessellated skeleton: insight form the skeletal growth of the round stingray *Urobatis halleri*. *J. Anat.* DOI:10.1111/j.1469-7580.2009.01116x (2009).

Dearden, R. P., Stockey C. & Brazeau, M.D. The pharynx of the stem-chondrichthyan *Ptomacanthus* and the early evolution of the gnathostome gill skeleton. *Nature Communications*, **10**, 2050. doi.org/10.1038/s41467-019-10032-3 (2019).

Denison, R. in *Handbook of Paleoichthyology* Vol. 2 (ed. Schultze, H.-P.) (Gustav Fischer Verlag, Stuttgart, 1978).

Denison, R. in *Handbook of Paleoichthyology* Vol. 5 (ed. Schultze, H.-P.) (Gustav Fischer Verlag, Stuttgart, 1979).

Dennis, K. D. & Miles, R. S. A pachyosteomorph arthrodire from Gogo, Western Australia. *Zoological Journal of the Linnean Society* **73**, 213-258 (1981).

Dick, J. R. R. On the Carboniferous shark *Tristychius arcuatus* Agassiz from Scotland. *Trans. R. Soc. Edinb.* **70**, 63–109 (1978).

Dick, J. R. R. *Diplodoselache woodi* gen. et sp. nov., an early Carboniferous shark from the Midland Valley of Scotland. *Trans. R. Soc. Edinb.* **72**, 99–113 (1981).

Dick, J. R. F. & Maisey, J. G. The Scottish Lower Carboniferous shark *Onychoselache traquairi*. *Palaeontology* **23**, 363-374 (1980).

Didier, D. A. Phylogenetic systematics of extant chimaeroid fishes (Holocephali, Chimaeroidei). *Am. Mus. Novit.* **3119**, 1–86 (1995).

Didier, D. A., Kemper, J. M. & Ebert, D. A. in *Biology of Sharks and Their Relatives* (eds Carrier, J. C., Musick, J. & Heithaus, M. R.) 97-122 (CRC Press, 2012).

Didier, D. A., Stahl, B. J. & Zangerl R. Development and growth of compound tooth plates in *Callorhinchus milii* (Chondrichthyes, Holocephali). *Journal of Morphology* **222**, 73-89 (1994).

Donoghue, P.C.J., & Aldridge, R. J. in *Major events in early vertebrate evolution: palaeontology, phylogeny, genetics and development.* (ed Ahlberg, P. E.) 85–105 (Taylor & Francis, 2001).

Donoghue, P. C. J., Forey, P. L., & Aldridge, R. J. Conodont affinity and chordate phylogeny. *Biol. Rev.***75**, 191–251 (2000).

Downs, J. P. & Donoghue, P. C. J. Skeletal histology of *Bothriolepis canadensis* (Placodermi, Antiarchi) and evolution of the skeleton at the origin of jawed vertebrates. *Journal of Morphology* **270**, 1364-1380 (2009).

Dupret, V., Sanchez, S., Goujet, D., Tafforeau, P. & Ahlberg, P. E. A primitive placoderm sheds light on the origin of the jawed vertebrate face. *Nature* **507**, 500-503 (2014).

Dupret, V., Sanchez, S., Goujet, D. & Ahlberg, P. E. The internal cranial anatomy of *Romundina stellina* Orvig, 1975 (Vertebrata, Placodermi, Acanthothoraci) and the origin of jawed vertebrates-Anatomical atlas of a primitive gnathostomes. *PLoS ONE* **12**: e0171241. DOI:10.1371/journal.pone.0171241 (2017).

Eriksson, T., *AutoDecay Version 5.0.* (2001).

Felsenstein, J. Confidence-limits on phylogenies - an approach using the bootstrap. *Evolution* **39**, 783-791 (1985).

Finarelli, J. A. & Coates, M. I. First tooth-set outside the jaws in a vertebrate. *Proceedings of the Royal Society of London Series B-Biological Sciences* **279,** 775-779 (2012).

Finarelli, J. A. & Coates, M. I. *Chondrenchelys problematica* (Traquair, 1888) redescribed: a Lower Carboniferous, eel-like holocephalan from Scotland. *Earth and Environmental Science Transactions of the Royal Society of Edinburgh* **105**, 1-25 (2014).

Fletcher, T., Altringham J., Peakall, J., Wignall, P., & Dorrell, R. Hydrodynamics of fossil fishes. *Proc. R. Soc. B* **281**, 20140703 (2014).

Forey, P. L. *Latimeria*: a paradoxical fish. *Proc. R. Soc. London, Ser. B* **208**, 369–384 (1980).

Frey, L., Coates, M., Ginter, M., Hairapetian, V., Rücklin, M., Jerjen, I., & Klug, C. The early elasmobranch *Phoebodus*: phylogenetic relationships, ecomorphology and a new time-scale for shark evolution. Proc. R. Soc. B 286: 20191336 (2019).

Friedman, M. *Styloichthys* as the oldest coelacanth: implications for early osteichthyan interrelationships. *J. Syst. Palaeontol.* **5**, 289–343 (2007).

Friedman, M. & Brazeau, M. A reappraisal of the origin and basal radiation of the Osteichthyes. *J. Vertebr. Paleontol.* **30**, 36–56 (2010).

Gagnier, P. Y. in *Devonian Fishes and Plants of Miguasha, Quebec, Canada.* (eds Schultze, H. P. & Cloutier, R.) 149–163 (Freidrich Pfeil, 1996).

Gagnier, P.-Y., Hanke, G. F. & Wilson, M. V. H. *Tetanopsyrus lindoei* gen. et sp. nov., an Early Devonian acanthodian from the Northwest Territories, Canada. *Acta Geologica Polonica* **49**, 81-96 (1999).

Gagnier, P.-Y. & Wilson, M. V. H. New evidences on jaw bones and jaw articulations in acanthodians. *Geobios* **19** (1995).

Gagnier, P.-Y. & Wilson, M. V. H. Early Devonian acanthodians from northern Canada. *Palaeontology* **39**, 241–258 (1996a).

Gagnier P. –Y. & Wilson M. V. H. An unusual acanthodian from Northern Canada: revision of *Brochoadmones milesi*. *Modern Geology* **20**, 235-251 (1996b).

Gai Z., Donoghue, P. C. J., Zhu, M., Janvier, P. & Stampanoni, M. Fossil jawless fish from China foreshadows early jawed vertebrate anatomy. *Nature* **476**, 324-367 (2011).

Gans, C. & Parsons, T. S. *A Photographic Atlas of Shark Anatomy* (Academic Press, New York, 1964).

Gardiner, B. G. The relationships of the palaeoniscoid fishes, a review based on new specimens of *Mimia* and *Moythomasia* from the Upper Devonian of Western Australia. *Bull. Br. Mus. Nat. Hist. (Geol.*) **37**, 173–428 (1984).

Gardiner B. G. & Bartram A. W. H. in *Problems in Vertebrate Evolution* (eds Andrews, S. M., Miles, R. S., Walker & A. D.) 227-245 (Linnean Symposium Series, London, 1977).

Gess R. W. & Coates M. I. High-latitude chondrichthyans from the Late Devonian (Famennian) Witpoort formation of South Africa. *Paläontol Z* DOI:10.1007/s12542-014-0221-9 (2014).

Giles, S., Coates, M., Garwood, R. J., Brazeau, M. D., Atwood, R., Johanson, Z. & Friedman, M. Enodskeletal structure in *Cherolepis* (Osteichthyes, Actinopterygii), an early ray-finned fish. *Palaeontology* 2015, DOI:10.1111/pala. 12182 (2015a).

Giles, S., Darras, L., Clément, G., Blieck, A. & Friedman, M. An exceptionally preserved Late Devonian actinopterygian provides a new model for primitive cranial anatomy in ray-finned fishes. *Proc. R. Soc.* B **282**: 20151485 (2015b).

Giles, S., Friedman, M. & Brazeau, M. D. Osteichthyan-like cranial conditions in an Early Devonian stem gnathostome. *Nature* **520,** 82-85 (2015c).

Goodrich E. S. *Studies on the structure and development of vertebrates* (Univ. Chicago Press, 1930).

Goujet D, & Young G. C. in *Recent Advances in the Origin and Early Radiation of Vertebrates*, (eds Arratia, G., Wilson, M. V. H. & Cloutier, R.) 109–26 (Friedrich Pfeil, 2004).

Grogan, E. D. & Lund, R. *Debeerius ellefseni* (Fam. Nov., Gen. Nov., Sped. Nov.) an Autodiastylic Chondrichthyan From the Mississippian Bear Gulch Limestone of Montana (USA), the Relationships of the Chondrichthyes, and Comments on Gnathostome Evolution. *J. Morphol.* **243**, 219–245 (2000).

Grogan, E. D., Lund, R. & Greenfest-Allen, E. in *Biology of Sharks and Their Relatives* (eds Carrier, J. C., Musick, J. A. & Heithaus, M. R.) 3-29 (CRC Press, 2012).

Gross, W. Histologischen Studien am Aussenskelett fossiler Agnathen und Fische. *Palaeontographica Abteilung A* **83**, 1-60 (1935).

Gross, W. Das Kopfskelett on *Cladodus wildungensis* Jaekel; 1. Teil. Endocranium und Palatoquadratum. *Senckenbergiana* **19**, 80-107 (1937).

Gross, W. Das Kopfskelett on Cladodus wildungensis Jaekel; 2. Teil. Der Kieferbogen. Anhang: *Protractodus vetusus* Jaekel. *Senckenbergiana* **20**, 123-145 (1938).

Gross, W. *Gemuendina stuertzi* Traquair. *Neuuntersuchung. Notizbl. Hess. Landesamt. Bodenf.* **91**, 36-73. (1963).

Gross, W. *Lophosteus superbus* Pander, ein Teleostome aus dem Silur Oesels. *Lethaia* **2**, 15-47 (1969).

Gross, W. *Lophosteus superbus* Pander: Zähne, Zahnknochen und besondere Schuppenformen. *Lethaia* **4**, 131-152 (1971).

Gross, W. Kleinschuppen, Flossenstacheln und Zähne von Fischen aus europäischen und nordamerikanischen Bonebeds des Devons. *Palaeontographica Abt A* **142**: 51–155 (1973)

Hampe, O. Revision of the Xenacanthida (Chondrichthyes: Elasmobranchii) from the Carboniferous of the British Isles. *Trans. R. Soc. Edinb. (Earth Sci.)* **93**, 191–237 (2003).

Hanke G. F. *Promesacanthus eppleri* n. gen., n. sp., a mesacanthid (Acanthodii, Acanthodiformes) from the Lower Devonian of northern Canada. *Geodiversitas* **30**, 287–302 (2008).

Hanke, G. F. & Davis, S. P. Redescription of the acanthodian *Gladiobranchus probaton* Bernacsek & Dineley, 1977, and comments on diplacanthid relationships. *Geodiversitas* **30**, 303-330 (2008).

Hanke, G. F. & Davis, S. P. A re-examination of *Lupopsyrus pygmaeus* Bernacsek & Dineley, 1977 (Pisces, Acanthodii). *Geodiversitas* **34**, 469-487 (2012).

Hanke, G. F., Davis, S. P. & Wilson, M. V. H. A new species of the acanthodian genus *Tetanopsyrus* from Northern Canada, with comments on related taxa. *J. Vert. Paleontol*. **21**, 740-753 (2001).

Hanke, G. F., & Wilson, M. V. H. in *Recent Advances in the Origin and Early Radiation of Vertebrates*. (eds Arratia, G., Wilson, M. V. H., & Cloutier, R.) 189–216 (Freidrich Pfeil, 2004).

Hanke, G. F. & Wilson, M. V. H. Anatomy of the Early Devonian Acanthodian *Brochoadmones milesi* based on nearly complete body fossils, with comments on the evolution and development of paired fins. *J. Vert. Paleontol*. **26**, 526-537 (2006).

Hanke, G. F., & Wilson, M. V. H. in *Morphology, Phylogeny and Paleobiogeography of Fossil Fishes*. (eds Elliot, D.K., Maisey, J.G., Yu, X. & Miao, D.) 159–182 (Freidrich Pfeil, 2010).

Harris, J. E. The dorsal fin spine of *Cladoselache*. *Scientific Publications of the Cleveland Museum of Natural History* **8**, 1-6 (1938a).

Harris, J. E. The neurocranium and jaws of *Cladoselache*. *Scientific Publications of the Cleveland Museum of Natural History* **8**, 7-12 (1938b).

Heidtke, U. H. J. Der Xenacanthide *Orthacanthus senckenbergianus* aus dem pfälzischen Rotliegenden (Unter-Perm). *Pollichia* **70**, 65-86 (1982).

Heidtke, U. Studien über *Acanthodes*. 4. *Acanthodes boyi* n. sp., die dritte Art der Acanthodier (Acanthodii: Pisces) aus dem Rotliegend (Unterperm) des Saar-Nahe-Beckens (S-W-Deutschland. *Paläont. Z.* **67**, 331-341 (1993).

Heidtke, U. H. J. *Orthacanthus* (*Lebachacanthus*) *senckenbergianus* Fritsch 1889 (Xenacanthida: Chondrichthyes): revision, organisation und phylogenie. *Freiberger Forschungsheft* **481**, 63-106 (1999).

Heidtke, U. H. J. *Gladbachus adentatus*, die Geschichte des weltweit ältesten Hais – untersucht und beschriebenaus dem AK Geowissenschaften. *Pollichia Kurrier* **25**, 24-26 (2009).

Heidtke, U. H. J. Neue Erkenntnisse über *Acanthodes bronni* Agassiz 1833. *Mitt. Pollichia* **95**, 1-14 (2011a).

Heidtke, U. H. J. Revision der unterpermischen Acanthodier (Acanthodii: Pisces) des südwestdeutschen Saar-Nahe-Beckens. *Mitt. Pollichia* **95**, 15-41 (2011b).

Heidtke, U. H. J. & Krätschmer K. *Gladbachus adentatus* nov. gen. et sp., ein primitiver Hai aus dem Oberen Givetium (Oberes Mitteldevon) der Bergisch Gladbach – Paffrath-Mulde (Rheinisches Schiefergebirge). *Mainzer geowiss. Mitt.* **30**, 105-122 (2001).

Heidtke, U. H. J., Schwind, C. & Krätschmer K. Über die Organisation des Skelettes und die verwandschaftlichen Beziehungen der Gattung Triodus Jordan 1849 (Elasmobranchii: Xenacanthida). *Mainzer geowiss. Mitt.* **32**, 9-54 (2004).

Hemmings, S. K. A monograph of the Old Red Sandstone antiarchs of Scotland: *Pterichthyodes* and *Microbrachius*. *Palaeontographical Society [Monographs]* **131**, 1-64 (1973).

Holland, T. Pectoral girdle and fin anatomy of *Gogonaus andrewsae* Long, 1985: Implications for tetrapodomorph limb evolution. *Journal of Morphology* **274**, 147-164 (2013).

Holland, T. & Long, J. A. On the phylogenetic position of *Gogonasus andrewsae* Long 1985, within the tetrapodomorpha. *Acta Zoologica* **90** (supplement 1), 285-296 (2009).

Hotton, N. Jaws and teeth of American xenacanth sharks. *Journal of Paleontology* **26**, 489-500 (1952).

Howard, L. E., Holmes, W. M., Ferrando, S., Maclaine, J. S., Kelsh, R. N., Ramsey, A., Abel R. L. & Cox P. L. J. Functional nasal morphology of chimaeroid fishes. *Journal of Morphology* **274**: 987-1009 (2013).

Hu, Y., Lu, J. & Young, G. C. New findings in a 400 million-year-old Devonian placoderm shed light on jaw structure and function in basal gnathostomes. *Scientific Reports* **7**: 7813. DOI:10.1038/s41598-017-07674-y (2017).

Janvier, P. *Les Céphalaspides du Spitsberg. Anatomie, phylogénie et systématique des Ostéostracés siluro-dévoniens. Révision des Ostéostracés de la Formation de Wood Bay (Dévonien inférieur du Spitsberg)* (Cahiers de Paléontologie, Section Vertebres, Centre National de la Recherche Scientifique, 1985).

Janvier, P. *Early Vertebrates* (Oxford Univ. Press, 1996).

Janvier, P. & Maisey, J. G. (2010) in *Morphology, Phylogeny and Paleobiogeography of Fossil Fishes*. (eds Elliot, D.K., Maisey, J.G., Yu, X. & Miao, D.) 431–459 (Friedrich Pfeil, 2010).

Jarvik, E. Middle and Upper Devonian Porolepiformes form East Greenland with special reference to *Glyptolepis groenlandica* n. sp. *Meddelelser om Grønland* **187**, 1-307 (1972).

Jarvik, E. in *Problems in Vertebrate Evolution.* (eds Andrews, S. M., Miles, R. S., Walker & A. D.) 199-225 (Linnean Symposium Series, London, 1977).

Jarvik, E. *Basic Structure and Evolution of Vertebrates* (Academic, 1980).

Johanson, Z. Placoderm branchial and hypobranchial muscles and origins in jawed vertebrates. *Journal of Vertebrate Paleontology* **23**, 735-749 (2003).

Johanson, Z., Underwood, C., Coates, M. I., Fernandez, V., Clark, B., Smith, M. M. (in press, 2020) . The stem-holocephalan Helodus (Chondrichthyes; Holocephali) and the evolution of modern chimaeroid dentitions. In: John Maisey symposium, Ichthyol explanation freshwaters (eds. Denton, J., Pradel, A. & Janvier, P.). (Verlag Pfeil, 2020).

Karatajūte-Talimaa, V. in *Fossil Fishes as Living Animals* (ed. Mark-Kurik, E.) 223-232 (Academy of Sciences of Estonia, Tallinn, 1992).

Kesteven, H. L. The anatomy of the head of *Callorhynchus antarcticus*. *Journal of Anatomy* **67**, 443-474 (1937).

King B., Qiao T., Lee M. S. Y., Zhu M. & Long J.A. Bayesian morphological clock methods resurrect placoderm monophyly and reveal rapid early evolution in jawed vertebrates. *Syst. Biol.* **2016**, syw107 (doi:10.1093/sysbio/syw107) (2016).

Lane, J. A. Morphology of the braincase in the Cretaceous hybodont shark *Tribodus linnae* (Chondrichthyes: Elasmobranchii), based on CT scanning. *Amer. Mus. Novitates* **2758**, 1-70 (2010).

Lane, J. A. & Maisey, J. G. Pectoral anatomy of *Tribodus limae* (Elasmobranchii: Hybodontiformes) from the Lower Cretaceous of Northeastern Brazil. *Journal of Vertebrate Paleontology* **29,** 25-38 (2009).

Lane, J. A. & Maisey, J. G. The visceral skeleton and jaw suspension in the durophagous hybodontid shark *Tribodus limae* from the Lower Cretaceous of Brazil. *Journal of Paleontology* **86,** 886-905 (2012).

Liem, K., & Summers, A. P. in *Sharks, Skates, and Rays, the Biology of Elasmobranch Fishes* (ed. Hamlett, W. C.) 93-114 (Johns Hopkins University, 1999).

Long, J. A. A new diplacanthoid acanthodian from the Late Devonian of Victoria. *Memoirs of the Association of Australasian Palaeontologists* **1**, 51-65 (1983).

Long, J. A. A new osteolepidid fish from the Upper Devonian Gogo Formation, Western Australia. *Records of the Western Australia Museum* **12**, 361-377 (1985).

Long, J. A. A new camuropiscid arthrodire (Pisces: Placodermi) from Gogo, Western Australia. *Zoological Journal of the Linnean Society* **94**, 233-258 (1988a).

Long, J. A. New palaeoniscoid fishes from the Late Devonian and Early Carboniferous of Victoria. *Memoirs of the Association of Australian Palaeontologists* **7**, 1-64 (1988b).

Long, J. A. Ptyctodontid fishes (Vertebrata, Placodermi) from the Late Devonian Gogo Formation, Western Australia, with a revision of the European genus *Ctenurella* Orvig, 1960. *Geodiversitas* **19**, 515–555. (1997).

Long, J. A. *The Rise of Fishes* second edition (John Hopkins Univ. Press, 2011).

Long, J. A., Barwick, R. E., & Campbell. Osteology and functional morphology of the osteolepiform fish *Gogonasus andrewsae* Long 1995, from the Upper Devonian Gogo Formation, Western Australia. *Records of the Western Australian Museum* Supplement **53,** 1-89 (1997).

Long, J. A., Burrow, C. J., Ginter, M., Maisey, J., Trinajstic, K. M., Coates, M. I., Young, G. C. & Senden, T. J. First shark from the Late Devonian (Frasnian) Gogo Formation, Western Australia sheds new light on the development of tessellated calcified cartilage**.** *PLoS ONE* **10**: e0126066 DOI:10.1371/journal.pone.0126066 (2015).

Long, J. A., Mark-Kurik, E., Johanson, Z, Lee, M. S. Y., Young, G. C., Zhu, M., Ahlberg, P. E., Newman, M., Jones, R., den Blaauwen, J., Choo, B. & Trinajstic, K. Copulation in antiarch placoderms and the origin of gnathostome internal fertilization. *Nature* **517**, 196-199 (2015).

Long, J. A., Trinajstic, K. M., & Johanson, Z. Devonian arthrodire embryos and the origin of internal fertilization in vertebrates. *Nature* **457**, 1124-1127 (2009).

Long, J. A., Young, G. C., Holland, T., Senden, T. J. & Fitzgerald, E. M. G. An exceptional Devonian fish from Australia sheds light on tetrapod origins. *Nature* **444,** 199-202 (2006).

Lu, J., Giles, S., Friedman, M., den Blaawen, J. L. & Zhu, M. The oldest actinopterygian highlights the cryptic early history of the hyperdiverse ray-finned fishes. *Current Biology* **26**, 1-7 (2016).

Lund, R. *Harpagofututor volsellorhinus* new genus and species (Chondrichthyes, Chondrenchelyiformes) from the Namurian Bear Gulch Limestone, *Chondrenchelys problematica* and their sexual dimorphism. *Journal of Paleontology* **56**, 938-958 (1982).

Lund, R. The morphology of *Falcatus falcatus* (St. John and Worthen), a Mississippian stethacanthid chndrichthyan from the Bear Gulch Limestone of Montana. *Journal of Vertebrate Paleontology* **5**, 1-19 (1985).

Lund, R. On *Damocles serratus* nov. gen. et sp., (Elasmobranchii: Cladodontida) from the Upper Mississippian Bear Gulch Limestone of Montana. *Journal of Vertebrate Paleontology* **6**, 12-19 (1986).

Lund, R. & Grogan, E. D. Relationships of the Chimaeriformes and the basal radiation of the Chondrichthyes. *Reviews in Fish Biology and Fisheries* **7**, 65-123 (1997).

Lund, R. & Grogan, E. D. in *Recent Advances in the Origin and Early Radiation of Vertebrates* (eds Arratia, G., Wilson, M. V. H. & Cloutier, R.) 171–187 (Friedrich Pfiel, 2004a).

Lund, R. & Grogan, E. D. in *Recent Advances in the Origin and Early Radiation of Vertebrates* (eds Arratia, G., Wilson, M. V. H. & Cloutier, R.) 505–531 (Friedrich Pfiel, 2004b).

Maisey, J. G. The anatomy and interrelationships of Mesozoic hybodont sharks. *Am. Mus. Novit.* **2724**, 1–48 (1982).

Maisey, J. G. Cranial anatomy of *Hybodus basanus* Egerton from the Lower Cretaceous of England. *Am. Mus. Novit.* **2758**, 1–64 (1983).

Maisey, J. G. Cranial Morphology of the Fossil Elasmobranch *Synechodus dubrisiensis*. *Am. Mus. Novit.* **2804**, 1-28 (1985).

Maisey, J. G. Visceral skeleton and musculature of a Late Devonian shark. *J. Vertebr. Paleontol*. **9**, 174–190 (1989a).

Maisey, J. G. *Hamiltonichthys mapesi* g. & sp. nov. (Chondrichthyes; Elasmobranchii), from the Upper Pennsylvanian of Kansas. *American Museum Novitates* **2931**, 1-42 (1989b).

Maisey, J. G. in *Major Events in Early Vertebrate Evolution* (ed. Ahlberg, P. E.) 263–288 (Taylor and Francis, 2001a).

Maisey, J. G. Remarks on the inner ear of elasmobranchs and its interpretation from skeletal labyrinth morphology. *J. Morph.* **250**, 236-264. (2001b).

Maisey, J. G. Braincase of the Upper Devonian shark *Cladodoides wildungensis* (Chondrichthyes, Elasmobranchii), with observations on the braincase in early chondrichthyans. *Bull. Am. Mus. Nat. Hist*. **288**, 1–103 (2005).

Maisey, J. G. The braincase in Paleozoic symmoriiform and cladoselachian sharks. *Bull. Am. Mus. Nat. Hist*. **307**, 1–122 (2007).

Maisey, J. G. The postorbital palatoquadrate articulation in elasmobranchs. *Journal of Morphology* **269**, 1022-1040 (2008).

Maisey, J. G. The spine-brush complex in symmoriiform sharks (Chondrichthys; Symmoriiformes), with commenst on dorsal fn modularity. *Journal of Vertebrate Paleontology* **29**, 14-24 (2009).

Maisey, J. G. The braincase of the Middle Triassic shark *Acronemus tuberculatus* (Bassani, 1886). *Palaeontology* **54**, 417-428 (2011).

Maisey, J. G. The diversity of tessellated calcification in modern and extinct chondrichthyans. *Revue de Paléobiologie, Geneve* **32,** 355-371 (2013).

Maisey, J. G. & Anderson, M. E. A primitive chondrichthyan braincase from the Early Devonian of South Africa. *Journal of Vertebrate Paleontology* **21**, 702-713 (2001).

Maisey, J. G. & de Carvalho, M. R. A new look at old sharks. *Nature* **385**, 779-780 (1997).

Maisey, J. G., Janvier, P., Pradel, A., Denton, J. S. S., Bronson, A., Miller, R. & Burrow, C. J. *Doliodus* and pucapampellids. In *Evolution and Development of Fishes* (eds Johanson, Z., Underwood, C. & Richter, M.) 87–109 (Cambridge, 2019).

Maisey, J. G. & Lane, J. A. Labyrinth morphology and the evolution of low-frequency phonoreception in elasmobranchs. *C. R. Palevol* **9**, 289-309. (2010)

Maisey, J. G., Miller, R. & Turner, S. The braincase of the chondrichthyan *Doliodus* from the Lower Devonian Campbellton Formation of New Brunswick, Canada. *Acta Zoologica* **90** (supplement 1), 109-122 (2009).

Maisey, J. G., Miller, R., Pradel, A., Denton, J. S. S., Bronson, A. & Janvier, P. Pectoral morphology in *Doliodus*: bridging the 'acanthodian'-chondrichthyan divide. *American Museum Novitates* **3875**, 1-15. (2017).

Maisey, J. G., Turner, S., Naylor, G. J. P. & Miller, R. F. Dental patterning in the earliest sharks: implications for tooth evolution. *Journal of Morphology*. DOI:10.1002/jmor.20242 (2013)

Mallat, J. The origin of the vertebrate jaw: neoclassical ideas versus newer, development-based ideas. *Zool. Sci*. **25,** 990-998 (2008).

Marinelli, W. & Strenger, A. *Vergleichende Anatomie und Morphologie der Wirbeltiere*. III. 174-308 (Franz Deuticke, Wien, 1959).

Miles, R. S. in *Current problems of lower vertebrate phylogeny*. (ed. T. Ørvig) 109-127 (Almqvist & Wiksell, Stockholm, 1968).

Miles, R. S. Acanthodii. in *Encyl. Sci. Tech*. 33–34, (McGraw-Hill,1971).

Miles, R. S. Articulated acanthodian fishes from the Old Red Sandstone of England, with a review of the structure and evolution of the acanthodian shoulder- girdle. *Bull. Br. Mus. Nat. Hist. (Geol.)* **24**, 111–213 (1973a).

Miles, R. S. in *Interrelationships of Fishes* (eds Greenwood, P. H., Miles, R. S. & Patterson, C.) 63–103 (Academic, 1973b).

Miles, R. S. & Westoll, T. S. The placoderm fish *Coccosteus cuspidatus* Miller ex Agassiz from the Middle Old Red Sandstone of Scotland. *Transactions of the Royal Society of Edinburgh* **67**, 373-476 (1968).

Miles, R. S. & Young, G. C. in *Problems in Vertebrate Evolution*. (eds Andrews, S. M., Miles, R. S., Walker & A. D.) 123–198 (Linnean Symposium Series, London, 1977).

Miller, R. F., Cloutier, R. & Turner, S. The oldest articulated chondrichthyan from the Early Devonian period. *Nature* **425**, 501–504 (2003).

Moy-Thomas, J. A. The structure and affinities of *Chondrenchelys problematica* Tr. *Proceedings of the Zoological Society, London***1935**, 391-403 (1935).

Mutter, R. J., de Blanger, K., & Neuman, A. G. Elasmobranchs from the Lower Triassic Sulphur Mountain Formation near Wapiti Lake (BC, Canada). *Zoological Journal of the Linnean Society* **149**, 309-37 (2007).

Mutter, R. J., Neuman, A. G. & de Blanger, K. *Homalodontus* nom. nov. a replacement for *Wapitiodus* Mutter, de Blanger and Neuman 2007 (Homalodontidae nom. nov., ?Hybodontoidea), preoccupied by *Wapitiodus* Orchard, 2005. *Zoological Journal of the Linnean Society* **154**, 419-420 (2008).

Moy-Thomas, J.A. & Miles, R. *Palaeozoic Fishes* 2nd edition (Chapman & Hall, 1971).

Nelson, G. J. in *Current problems of lower vertebrate phylogeny*. (ed. T. Ørvig) 129-143 (Almqvist & Wiksell, Stockholm, 1968).

Nelson, G. J. Gill arches and the phylogeny of fishes, with notes on the classification of vertebrates. *Bull. Am. Mus. Nat. Hist.* **141**, 475–552 (1969).

Nelson, G. J. Pharyngeal denticles (placoid scales) of sharks with notes on the dermal skeleton of vertebrates. *Amer. Mus. Novitates* **2416**, 1-26 (1970).

Newman, M. J., Davidson, R. G., den Blaauwen, J. L. & Burrow, C. J. The Early Devonian acanthodian *Uraniacanthus curtus* (Powrie, 1870) n. comb., from the Midland Valley of Scotland. *Geodiversitas* **34**, 739-759 (2012).

Nielsen, E. Studies on Triassic fishes I. *Meddelelser om Grønland* **138**, 1–403 (1942).

Oelofsen, B. W. in *Indo-Pacific Fish Biology: Proceedings of the Second International Conference on Indo-Pacific Fishes.* (eds Uyeno, T., Arai, R., Taniuchi, T., Matsuura, K.) 107-124 (Ichthyological Society of Japan, Tokyo, 1986).

Ørvig, T. Histological studies of placoderms and fossil elasmobranchs 1. The endoskeleton, with remarks on the hard tissues of lower vertebrates in general. *Arkiv för Zoologie* **2**, 321-454 (1951).

Ørvig, T. New finds of acanthodians, arthrodires, crossopterygians, ganoids and dipnoans in the upper Middle-Devonian Calcareous Flags (Oberer Plattenkalk) of the bergisch-Paffrath Trough (prt 1). *Paläontologische Zeitschrift* **34,** 295-335 (1960).

Ørvig, T. in *Structural and Chemical organization of Teeth*. (ed. Miles, A. E. W.) 45-110 (Academic Press, London1967).

Otto, M. Zur systematischen Stellung der Lophosteiden (Obersilur, Pisces inc. sedis). *Paläontologische Zeitschrift* **65**, 345-350 (1991).

Patterson, C. The phylogeny of the chimaeroids. *Philosophical Transactions of the Royal Society of London*, Series B **249**, 101-219 (1965).

Patterson, C. Morphology and interrelationships of primitive actinopterygian fishes. *American Zoologist* **22**, 241-259 (1982).

Patterson, C. Interpretation of the toothplates of chimaeroid fishes. *Zool. J. Linn. Soc.* **106**, 33-61 (1992).

Pearson, D. M. & Westoll, T. S. The Devonian actinopterygian *Cheirolepis* Agassiz. *Trans. R. Soc. Edinb.* **70**, 337–399 (1979).

Pradel, A. Skull and brain anatomy of Late Carboniferous Sibyrhynchidae (Chondrichthyes, Iniopterygia) from Kansas and Oklahoma (USA). *Geodiversitas* **32**, 595-661 (2010).

Pradel A., Didier, D., Casane, D., Tafforeau, P. & Maisey J. G. Holocephalan embryo provides new information on the evolution of the glossopharyngeal nerve, metotic fissure and parachordal plate in gnathostomes. *PLoS ONE* **8**: e66988 DOI:10.1371/journal.pone.0066988 (2013).

Pradel A., Langer M., Maisey J. G., Geffard-Kuriyama D., Cloetens P., Janvier P., & Tafforeau P. Skull and brain of a 300 million-year-old chimaeroid fish revealed by synchrotron holotomography. *Proceedings of the National Academy of Sciences* **106**, 5224-5228 (2009b).

Pradel, A., Maisey, J. G., Tafforeau, P., & Janvier, P. An enigmatic gnathostome vertebrate skull from the Middle Devonian of Bolivia. *Acta Zoologica* **90** (supplement 1), 123-133 (2009a).

Pradel, A., Maisey, J. G., Tafforeau, P., Mapes, R. H. & Mallatt, J. A Palaeozoic shark with osteichthyan-like branchial arches. *Nature* **509**, 608-611 (2014).

Pradel, A., Tafforeau, P. & Janvier P. Study of the pectoral girdle and fins of the Late Carboniferous sibyrhynchid iniopterygians (Vertebrata, Chondrichthyes, Iniopterygia) from Kansas and Oklahoma (USA) by means of microtomography, with comments on iniopterygian relationships. *Comptes Rendus Palevol* **9**, 377-387 (2010).

Pradel A., Tafforeau P., Maisey J. G. & Janvier P. A new Paleozoic Symmoriiformes (Chondrichthyes) from the Late Carboniferous of Kansas (USA) and Cladistic Analysis of Early Chondrichthyans. *PLoS ONE* **6**: e24938 DOI:10.1371/journal.pone.0024938 (2011).

Qu, Q.-M., Zhu, M. & Wang, W. Scales and dermal skeletal histology of an early bony fish *Psarolepis*

romeri and their bearing on the evolution of rhombic scales and hard tissues. *PLoS One* **8,** e61485 (2013a).

Qu, Q., Sanchez, S., Blom, H., Tafforeau, P. & Ahlberg P. E. Scales and Tooth Whorls of Ancient Fishes Challenge Distinction between External and Oral 'Teeth'. *PLoS ONE* **8**, DOI:10.1371/journal.pone.0071890 (2013b).

Qiao, T., King B., Long J. A., Ahlberg P. E., & Zhu M. Early Gnathostome Phylogeny Revisited: Multiple Method Consensus. *PLoS ONE* **11**, DOI:10.1371/journal.pone.0163157 (2016).

Reif, W.-E. Types of morphogenesis of the dermal skeleton in fossil sharks. *Paläont. Z.* **52**, 110-128 (1978).

Reif, W.-E. Squamation and ecology of sharks. *Cour. Forsch.-Inst. Senckenberg* **78**, 1-255 (1985).

Rieppel, O. A new genus of shark from the Middle Triassic of Monte San Giorgio, Switzerland. *Palaeontology* **25**, 399-412 (1982).

Ritchie, A. *Cowralepis*, a new genus of phyllolepid fish (Pisces, Placodermi) from the late Middle Devonian of New South Wales, Australia. *Proc. Linn. Soc. N.S.W.* **126**, 215–259 (2005).

Rücklin, M., Donoghue, P. C., Johansen, Z., Trinajstic, K., Marone, F. & Stampanoni, M. Development of teeth and jaws in the earliest jawed vertebrates. *Nature* **491**, 748-751 (2012).

Sansom, R. Phylogeny, classification and character polarity of the Osteostraci (Vertebrata). *Journal of Systematic Palaeontology* **7**, 95-115 (2009).

Schaeffer, B. The xenacanth shark neurocranium, with comments on elasmobranch monophyly. *Bull. Am. Mus. Nat. Hist.* **169**, 1–66 (1981).

Schultze, H.-P. & Märss, T. Revisiting *Lophosteus* Pander 1856, a primitive osteichthyan. *Acta Universitatis Latviensis* **679**, 57-78 (2004).

Schultze, H.NP. & J. Zidek. Ein primitiver acanthodier (Pisces) aus dem Unterdevon Lettlands. *Paläontologische Zeitschrift* **56**, 95-105 (1982).

Seidel, R., Lyons, K., Blumer, M., Zalansky, P., Fratzl, P., Weaver, J. C. & Dean, M. N. Ultrastructural and developmental features of the tessellated endoskeleton of elasmobranchs (sharks and rays). *J. Anat.* DOI:10.1111/joa.12508 (2016).

Sequeira, S. E. K. & Coates, M. I. Reassessment of 'Cladodus' neilsoni Traquair: a primitive shark from

Smith, M. M. & Sansom, I. J. in *Development, Function and Evolution of Teeth.* (eds Teaford, M. F., Smith, M. M. & Ferguson, M. W. J.) 65-81 (Cambridge University Press, Cambridge, 2000).

Solér-Gijon, R. & Hampe, O. Evidence of *Triodus* Jordan 1849 (Elasmobranchii: Xenacanthidae) in the Lower Permian of the Autun basin (Muse, France). *N. Jb. Geol. Paläont. Mh.* **1998**, 335-348 (1998).

Stahl, B. J. in *Handbook of Paleoichthyology* Vol. 4 (ed. Schultze, H.-P.) (Friedrich Pfeil, München. 1999).

Stensiö, E. A. On the head of the macropetalichthyids, with certain remarks on the head of the other arthrodires. *Publications of the Field Museum of Natural History (Geology)* **4**, 87-197 (1925).

Stensiö, E. A. Anatomical studies on the arthrodiran head (Part I). *K. Sv. Vet. Akad. Handl. Ser. 4., Bd. 8, Nr. 1. 8* (1963).

Stensiö, E. in *Traité de Paléontologie* (ed. Piveteau, J.) 71-692 (Masson, Paris, 1969).

Swofford, D. L. *PAUP*. Phylogenetic analysis using parsimony (* and other methods), version 4.0a147* (Sinauer, Sunderland, 2003).

Swofford, D. L. & Maddison, W. P. Reconstructing ancestral character states under Wagner parsimony. *Math. Biosci.* **87**, 199-229 (1987).

Thomson, K. S. The endocranium and associated structures in the Middle Devonian rhipidistian fish *Osteolepis*. *Proceedings of the Linnean Society of London* **176**, 181-195 (1965).

Trinajstic, K, Long, J. A., Johanson, Z., Young, G. and Senden, T. New morphological informationon the ptyctodontid fishes (Placodermi, Ptyctodontida) from Western Australia. *Journal of Vertebrate Paleontology* **32**: 757-780 (2012).

Trinajstic, K., Boisvert, C., Long, J., Maksimenko, A. & Johanson, J. Pelvic and repsoductive structures in placoderms (stem gnathostomes). *Biol. Rev*. (2014) 1-35.

Tucker A. S. & Fraser, G. J. Evolution and developmental diversity of tooth regeneration. *Semin Cell Dev Biol* DOI:10.1016/j.semcdb.2013.12.013 (2014).

Turner, S., Burrow, C. J. & Warren, A. *Gyracanthides hawkinsi* sp. nov. (Acanthodii, Gyracanthidae) from the Lower Carboniferous of Queensland, Australia, with a review of gyracanthid taxa. *Palaeontology* **48**, 963-1006 (2005).

Valiukevicius, J. in *Fossil Fishes as Living Animals* (ed. Mark-Kurik, E.) 193-213 (Academy of Sciences of Estonia, Tallinn, 1992).

Warren, A., Currie, B. P., Burrow, C. & Turner, S. A redescription and reinterpretation of *Gyracanthides murrayi* Woodward 1906 (Acanthodii, Gyracanthidae) from the Lower Carboniferous of the Mansfield Basin, Victoria, Australia. *Journal of Vertebrate Paleontology* **20**, 225-242 (2000).

Watson, D. M. S. The acanthodian fishes. *Philosophical Transactions of the Royal Society of London B* **228**, 49-146 (1937).

Wang, N.-Z., Donoghue, P. C. J., Smith, M. M. & Sansom, I. J. Histology of the galeaspid dermoskeleton and endoskeleton, and the origin and early evolution of the vertebrate cranial endoskeleton. *Journal of Vertebrate Paleontology* **25**, 745-756 (2005).

Westoll, T. S. On the structures of the dermal ethmoid shield of *Osteolepis*. *Geological Magazine* **73**, 157-171 (1936).

Williams, M. E. A new specimen of *Tamiobatis vetustus* (Chondrichthyes, Ctenacanthoidea) from the Late Devonian Cleveland Shale of Ohio. *Journal of Vertebrate Paleontology* **18**, 251-260 (1998).

Williams, M. E. Tooth retention in cladodont sharks: with a comparison between primitive grasping and swallowing, and modern cutting and gouging feeding mechanisms. *Journal of Vertebrate Paleontology* **21**, 214-226 (2001).

Woodward, A. S. & White, E. I. The dermal tubercles of the Upper Devonian shark, *Cladoselache*. *Annals and Magazine of Natural History* **11**, 367-368 (1938).

Young, G. C. New information on the structure and relationships of *Buchanosteus* (Placodermi: Euarthrodira) from the Early Devonian of New South Wales. *Zool. J. Linn. Soc*. **66**, 309–352 (1979).

Young, G. C. A new early Devonian placoderm from New South Wales, Australia, with a discussion of placoderm phylogeny. *Palaeontographica* A**167**, 10–76 (1980).

Young, G. C. Devonian sharks from South-Eastern Australia and Antarctica. *Palaeontology* **25**, 817–843 (1982).

Young, G. C. Reconstruction of the jaws and braincase in the Devonian placoderm fish *Bothriolepis*. *Palaeontology* **27**, 625-661 (1984).

Young, G. C. The relationships of placoderm fishes. *Zool. J. Linn. Soc*. **88**, 1–57 (1986).

Young, G. C. Antiarchs (placoderm fishes) from the Devonian Aztec sandstone, southern Victorian Land, Antarctica. *Palaeontographica*, A **202**, 1-125 (1988).

Young, G. C., Lelièvre, H. & Goujet, D. Primitive jaw structure in an articulated brachythoracid arthrodire (placoderm fish; Early Devonian) from southeastern Australia. *Journal of Vertebrate Paleontology* **21**, 670-678 (2001).

Yu, X. A new porolepiform-like fish, *Psarolepis romeri*, gen. et sp. nov. (Sarcopterygii, Osteichthyes) from the Lower Devonian of Yunnan, China. *J. Vertebr. Paleontol*. **18**, 261-264 (1998).

Zangerl, R. in *Handbook of Paleoichthyology* Vol. 3A (ed. Schultze, H.-P.) (Gustav Fischer Verlag, Stuttgart,1981).

Zangerl, R. Two new stethacanthid sharks (Stethacanthidae, Symmoriida) from the Pennsylvanian of Indiana, USA. *Palaeontographica*, A, **213**, 115–141 (1990).

Zangerl, R. & Case, G. R. Iniopterygia, a new order of chondrichthyan fishes from the Pennsylvanian of North America. *Fieldiana: Geology* **6**, 1-67 (1973).

Zangerl, R. & Case, G. R. *Cobelodus aculeatus* (Cope), ananacanthous shark from Pennsylvanian black shales of North America. *Palaeontographica*, A, **154**, 107–157 (1976).

Zhang, G.-R., Wang, J.-Q. & Wang, N.-Z. The structure of pectoral fin and tail of Yunnanolepidoidei, with a discussion of the pectoral fin of chuchinolepids. *Vertebrat. PalAsiatic*. **39**, 1–13 (2001).

Zhu, M. & Ahlberg, P. E. The origin of the internal nostril of tetrapods. *Nature* **432**, 94-97 (2004).

Zhu, M., Ahlberg, P., Pan, Z., Zhu, Y., Qiao, T., Zhao, W., Jia, L. & Lu, J. A Silurian maxillate placoderm illuminates jaw evolution. *Science* **354**, 334-336 (2016).

Zhu, M. & Schultze, H. P. The oldest sarcopterygian fish. *Lethaia* **30**, 293-304 (1997).

Zhu, M. & Schultze, H. P. in *Major Events in Early Vertebrate Evolution (Palaeontology, phylogeny, genetics, and development).* (ed. Ahlberg, P. E.) 289–314 (Taylor and Francis, 2001).

Zhu, M & Yu, X. A primitive fish close to the common ancestor of tetrapods and lungfish. *Nature* 418, 767-770 (2002).

Zhu, M. & Yu, X.-B. in *Recent Advances in the Origin and Early Radiation of Vertebrates* (eds Arratia, G., Wilson, M. V. H. & Cloutier, R.) 271-286 (Verlag Dr. Friedrich Pfeil, 2004).

Zhu, M., Yu, X, & Ahlberg, P. E. A primitive sarcopterygian fish with an eyestalk. *Nature* **410**, 81-84 (2001).

Zhu, M., Yu, X., Ahlberg, P. E., Choo, B., Lu, J., Qiao, Q., Zhao, L. J., Blom, H., & Zhu, Y. A Silurian placoderm with osteichthyan-like marginal jaw bones. *Nature* **52**, 188-193 (2013).

Zhu, M., Yu, X. & Janvier, P. A primitive fossil fish sheds light on the origin of bony fishes. *Nature* **397**, 607–610 (1999).

Zhu, M., Yu, X.-B., Choo, B., Wang, J.-Q. & Jia, L.-T. An antiarch placoderm shows that pelvic girdles arose at the root of jawed vertebrates. *Biol. Lett*. **8**, 453–456 (2012).

Zhu, M., Yu, X.-B., Wang, W., Zhao, W.-J. & Jia, L.-T. A primitive fish provides key characters bearing on deep osteichthyan phylogeny. *Nature* **441**, 77-80 (2006).

Zhu, M., Wang, W. & Yu, X.-B. *Meemannia eos*, a basal sarcopterygian fish from the Lower Devonian of China -expanded description and significance. In *Morphology, Phylogeny and Paleobiologepgraphy of Fossil Fishes*. D. K. Elliot, J. G. Maisey, X.-B. Yu & D.-S. Miao, eds (München: Verlag Dr. Friedrich Pfeil), pp. 199-214. (2010).

Zhu, M., Zhao, W., Jia, L., Lu, J., Qiao, T. & Qu, Q. The oldest articulated osteichthyan reveals a mosaic of gnathostome characters. *Nature* **458**, 469-474 (2009).

Zidek, J. Late Pennsylvanian Chondrichthyes, Acanthodii, and deep-bodied Actinopterygii from the Kinney Quarry, Manzanita Moutains, New Mexico. In *Geology and paleontology of the Kinney Brick Quarry, Late Pennsylvanian, central New Mexico*. J. Zidek ed. Bulletin 138, New Mexico Bureau of Mines & Mineral Resources (Socorro: New Mexico Bureau of Mines & Mineral Resources), pp. 199-214. (1992).
