# Supplementary material for: A symmoriiform from the Late Devonian of Morocco demonstrates a derived jaw function in ancient chondrichthyans
Source: Commun Biol. 2020 Nov 17;3:681. doi: 10.1038/s42003-020-01394-2 (PMC7672094; doi:10.1038/s42003-020-01394-2)
Supplement: Supplementary file 2 — Reporting Summary [file 42003_2020_1394_MOESM2_ESM.pdf]

Reviewers' comments:

Reviewer #1 (Remarks to the Author):

This manuscript describes a very well-preserved specimen of a Late Devonian symmoriiform chondrichthyan, Ferromerum. Importantly the jaws and hyoid arch are particularly well-preserved, allowing for detailed description and a reconstruction of the physical relationships between these units. Suprisingly, the authors suggest a unique lateral component of jaw rotation during feeding. I have only minor comments on the attached, in particular, I'm curious about the calcified cartilage of the skeleton- can this be described further, and would this relate to jaw/feeding function?

Reviewer #2 (Remarks to the Author):

Overall: A most interesting paper that is a valuable contribution to our knowledge of early shark evolution and anatomy and the understanding of generalized gnathostome feeding mechanisms. The descriptions are based on the well-preserved specimen plus extensive segmentation of CT scans which enable 3D restoration of most of the internal structures.
The fact that soft tissues are well preserve d in the specimen (liver, gut) indicates it is an exceptional specimens so there is no hesitation accepting the interpretations of CT data as reliable. The interpretaions about the feeding mechanism, utilising a unique (for sharsks) 'hemimandibular long axis rotation of the jaws' makes this paper particularly significant in showingt the high level of disparity and plasticity in feeding biomechanics for Devonians sharks, a group generally thought to be basal in many respects.

SPECIFIC POINTS /CORRECTIONS

Line 15 subject is singular (the fossil record…) so should be ‶ is poor″ not ‶are′

Linbes 73-80. Diagnosis is based on one sample onl. Spo relative terms like lareg or medium might not mena much unless actual rations included -eg large orbits relative to what -skull or head length? Give a ratio so we can compare with other sharks to see if it really is proportinately larger than say Cobeldus?.

As the majority of fossil sharks are known and defined only by their teeth I would like to see afew statemnst defining the teeth unique features added to the diagnosis. This would be very useful for future workers who might find isolated teeth of this species (eg cut/paste from lines 181-183).

Line 140. Reference to Brazeau & Ahlberg (2006) is to Panderichthys, a highly advanced sarcopterygian with a spiracular opening on top of the head, not laterally. I don’t think this is relevant here to sharks, perhaps better to use a reference to spiracular pouches in chondrichthyans?

Line 163 Fig 5 e,f there is no e, f in Fig 5, do you mean Fig 3 as well? Please correct.

Line 203-04: in diagnosis (lines 78-79) it is states ‶ no basihyal ‶ yet this is not discussed in the decription of the gilliarches, should be included. Perhaps it is softer cartilage or not preserved for some reason?

Phylogeny: noted that the symmoriform clade is weakly supported and discussed as so (line 241).

Figures

The figures are excellent and go beyond what is normally presented in such papers by offering complete models of the 3-D printed scan data which enable greater accuracy in interpretation of

functional biomechanics of the jaws and feeding mechanism,.

Fig. 4 'Iniopera' incorrect-should be –"Inioptera"

Reviewer #3 (Remarks to the Author):

This paper is a remarkable piece of work comprising two main points.
First describe a new early shark (unusually well 3-d preserve), Ferromirum oukherbouchi gen. and sp.nov, from the Late Devonian of the Anti-Atlas. Authors detailly analyse its systematic affinity, as well as the anatomical and phylogenetic implications provide by the new discovery.
The second main point investigates the morphology and biomechanics of the very well, 3D, preserved jaws and hyoid arch of Ferromirum in the context of early chondrichtyans. Authors reliably demonstrate the existence of a novel specialized jaw articulation revealing hemimandibular long-axis rotation. This the pattern of jaw motion is unknown in current fishes and suggest an increased morph- functional diversity in early gnathostomates

Overall impression of the work
Excellent work.

The paper is clearly writed and well structured. Figures are well performed and support the text. I consider the number of figures appropriate
This work would be of high-interest to a broad audience, (not only Paleobiology but also Biomechanics, zoology, anatomy, evolution, ecology ….) and materially contributes to a number of significant debates about early gnathostome evolution.

All of the methods used are correct for the studies in hand. Specimen collection and hosted is well documented and appropriate. Phylogenetic analysis are properly conducted. Data set is updated and extensive and it is available as sup. Information. I agree with the topology of tree in figure 4 (even the nesting of Gladbachus).
Overall, I would recommend this paper for publication in Communications Biology. My only minor concerns two aspects of the manuscript.

1- Some parts of the description of the new specimen are not merely description but include comparisons and inferences from the authors that could be better placed in a Remarks section (or easier, moved to next section Anatomical significance).
2- Authors objectively demonstrate morphological and consequent biomechanics diversity in jaw articulation of early chondrichtyans. However, they also claim for an associate an increased ecological disparity. I can accept this point, but it seems not clearly addressed in the paper (see the annotated document)

For more detailed comments seethe attached word file.

.

Reviewer 1 Review Attachment

A new symmoriiform from the Late Devonian of Morocco: novel jaw function in ancient sharks

L. Frey[1*], M. I. Coates[2], K. Tietjen[3], M. Rücklin[4] and C. Klug[1]

[1]Paläontologisches Institut und Museum, University of Zurich, Karl-Schmid-Strasse 4, CH-8006 Zürich (linda.frey@web.de; chklug@pim.uzh.ch).

[2]Department of Organismal Biology and Anatomy, University of Chicago, 1027 E. 57th St., USA-60637 Chicago.

[3]Biodiversity Institute, University of Kansas, 1345 Jayhawk Blvd, USA-66045 Lawrence.

[4] Naturalis Biodiversity Center, Postbus 9517, 2300 RA, Leiden, The Netherlands.

The fossil record of early chondrichthyans (sharks, rays, chimaeras, extinct relatives) and thus our knowledge of their anatomy and functional morphology are poor because of their predominantly cartilaginous skeletons. Here, we report a new symmoriiform shark, *Ferromirum oukherbouchi*, from the Late Devonian of the Anti-Atlas. Computed tomography scanning reveals the undeformed shape of the jaws and hyoid arch, which are of a kind often used to represent primitive conditions for jawed vertebrates. Of critical importance, these closely fitting cartilages preclude the repeatedly hypothesized presence of a complete gill between mandibular and hyoid arches. We show that the jaw articulation is specialized and drives mandibular rotation outward when the mouth opens, and inward upon closure. The resultant eversion and inversion of the lower dentition presents a greater number of teeth to prey through the bite-cycle. This suggests an increased functional and

ecomorphological disparity among chondrichthyans preceding and surviving the end-Devonian extinctions.

The Symmoriiformes is a widely distributed group of early chondrichthyans ranging from the Late Devonian through to the early Permian, and perhaps extending as far as the Cretaceous 1-4. Like the vast majority of early sharks, symmoriiforms have mostly been understood from two-dimensional skeletal and, more rarely, soft tissue remains, supplemented with insights from hard tissue histology 5-10. Symmoriiforms, occasionally including the classic Devonian genus *Cladoselache*, have often been used to exemplify early chondrichthyan conditions, and, from entrenched views of sharks as intrinsically primitive, generalised gnathostome conditions. Consistent with this treatment, both traditional and some of the more current phylogenetic hypotheses have placed this group on the chondrichthyan stem 11,12. However, an alternative series of analyses resolve symmoriiforms as stem holocephalans 2,3,10,13,14. Notably, the most recent of these employ the abundance of new data from X-ray tomography, especially concerning the neurocranium 3,11,15,16.

Here, we describe a recently discovered symmoriiform shark from the Devonian of Morocco, analyse its systematic affinity, discuss the phylogenetic implications, and investigate the morphology and motion of the superbly preserved jaws and hyoid arch. These jaws display the classic 'cleaver' shape 17 palate seen in a vast array of early crown-gnathostomes, and especially among chondrichthyans 2. However, in this unique specimen, the areas of attachment to the neurocranium and the jaw joint are undistorted, and likewise the slender cartilages of the hyoid arch. Crucially, these conditions allow a three-dimensional reconstruction of the feeding apparatus, and show that the hyoid arch was functionally associated with the palate and mandible. Further to this, physical modelling of the jaws reveals hemimandibular long-axis rotation, an action thus far unrecorded in studies of the feeding mechanics of living chondrichthyans 18, although recently recognised as an essential

component in the evolution of mammalian feeding systems [19]. These results add to new appreciation of chondrichthyans as early specialists in the gnathostome crown clade [20], and add to an emerging picture of functional disparity and likely ecomorphological partitioning among these early members of the modern vertebrate biota [21].

Results and discussion

Stratigraphy. Famennian, *Planitornoceras euryomphalum* to *Afrolobites mrakibensis* Zone; Ibâouane Formation, Lahfira Member, Thylacocephalan Layer (formerly described as Phyllocarid Layer [14,22,23], Madene el Mrakib, Maïder Basin, southeastern Anti-Atlas, Morocco.

Systematic palaeontology. Chondrichthyes Huxley, 1880 [24]

Total-group Holocephali Bonaparte, 1832 [25]

Symmoriiformes Zangerl, 1981 [1]

Ferromirum gen. nov.

Etymology: Derived from *ferrum* (lat. - iron) and *mirus* (lat. - miraculous). *Ferrum* refers to the preservation of the holotype in a reddish ferruginous nodule, which is characteristic for fossils from the Thylacocephalan Layer of the Maïder. *Mirus* refers to our initial misinterpretation of the gill remains of the holotype as crustacean appendages, before preparation and the miracle-like revelation that the specimen was, in fact, a chondrichthyan.

Type species: *F. oukherbouchi* sp. nov.

Diagnosis: A small symmoriid with slender body; head with short triangular rostrum and subterminal gape; supraorbital shelf with concave lateral margin; orbits large with sclerotic ring; narrow interorbital space; narrow suborbital shelf; cleaver-shaped palatoquadrate with anterolaterally directed articulation with postorbital process; scalloped margins of gape suggest around nine upper and lower tooth families; slender ceratohyal with posteroventral

lateral flange engaging with Meckel's cartilage; paired hypohyals anteriorly directed; no basihyal; distinguished from all other symmoriids by presence of pectoral-level, slender dorsal fin spine, smooth with posteriorly curved apex.

Ferromirum oukherbouchi sp. nov.

Etymology. The species name *oukherbouchi* honours the finder of the specimen Said Oukherbouch (Tafraoute).

Holotype. PIMUZ A/I 4806

Material. Holotype alone.

Locality. Madene El Mrakib, Maïder Basin, southeastern Anti-Atlas, Morocco.

Formation and age. Ibâouane Formation (middle Famennian, Upper Devonian), *Planitornoceras euryomphalum* to *Afrolobites mrakibensis* Zone.

Diagnosis. As for genus.

Description. The estimated body length of *Ferromirum oukherbouchi* gen. et sp. nov. is 330 mm. The specimen was prepared and exposed from its ventral side, revealing parts of the left orbit, mandibular, hyoid and branchial arches, pectoral and pelvic girdles (Fig. 1a-e; Supplementary Fig. 1). Substantial replacements of soft tissues are present throughout the body. Anteriorly, these reveal that the snout forms a short, pointed rostrum, resembling examples preserved in *Falcatus* and *Damocles* [7,8]. As in these genera, there is no evidence of skeletal support for the rostral apex, which likely housed arrays of electro- and mechanoreceptive organs (possible remains are visible as minute circular pits on its venter; Supplementary Fig. 2i). The trunk region includes two elongate lobes of the liver (Fig. 1d), extending for perhaps 50% of the visceral cavity length. Part of the digestive tract is evident as a spiral valve, exposed in the midline between the caudalmost extremities of both liver lobes (Fig. 1a,b). Directly anterior to the pelvic plates, a large bolus of material might represent a mass of pre-rectal gut contents (Fig. 1a,b,e).

Comment [WU1]: Can you label these? I'm not sure what you're referring to in the SI Figure 2i

Comment [WU2]: How does this compare to recent chondrichthyans?

Comment [WU3]: Also in Figure 1d- there's no question mark in this figure, but there is in Fig. 1b?

The computed tomograms reveal details of the braincase, jaws, hyoid arch, gill skeleton, pectoral girdle and dorsal fin spine (Figs. 2, 3, Supplementary Fig. 2i). However, the radiographic contrast between calcified cartilage and the surrounding matrix is often poor. The general shape of the neurocranium resembles that of *Ozarcus* 15,16 and *Dwykaselachus* 3, but the *Ferromirum* gen. nov. neurocranium has suffered post mortem compaction, possibly losing around 25% of its dorsoventral height (Figs 2d, 3c,d). The orbit is large: the maximum span equals the rostrocaudal lengths of otic and occipital regions combined 3. Remains of a slender sclerotic ring are exposed (Fig. 1a-c), but more detailed morphological information is not preserved. Sclerotic rings are known in *Cladoselache* 26, *Denaea* 6, *Falcatus* 7 and *Damocles* 8. A large opening for the optic nerve II perforates the mid-ventral part of the interorbital wall (Fig. 3b), and the slightly expanded anterior margin of the narrow suborbital shelf (Fig. 3b) signals presence of an articulation surface for the palatine ramus of the palatoquadrate.

Comment [WU4]: Can you describe this calcified cartilage in more detail? And in particular, how this might be related to jaw function and feeding- a stiffer jaw, versus less stiff?

The postorbital process and arcade (Fig. 3a-d) does not appear to have projected as far laterally and ventrally as those of *Dwykaselachus*, *Ozarcus* and *Akmonistion*. Rather, the process in *Ferromirum*, although likely incomplete, appears shorter, perhaps slightly more robust, proximally, and resembles that of *Gutturensis* 27. Similarly, the *Ferromirum* supraorbital shelf is narrow with a concave lateral margin, a feature also shared by *Ozarcus* as well as *Gutturensis*. Little detail of the otic region is recognizable. The condition of the endolymphatic duct(s) is unclear, but there is no trace of a parietal fossa (Fig. 3a). Traces of the otico-occipital fissure are preserved, revealing that the dorsal portion of the occipital unit is wedged between the otic capsules. The otic wall is too poorly preserved to reveal the presence of a periotic process. The ventral surface of the neurocranium (Fig. 3b) includes the characteristic narrow waist of symmoriids 3,16. The otic region and the glossopharyngeal canals are floored by a hypotic lamina (Fig. 3b). The openings of any canals or grooves for

the dorsal aortae, expected to be present in the basicranium, are not visible. However, the occipital plate (Fig. 3d) retains a discernible foramen magnum.

Unlike the braincase, the three-dimensional form of the mandibular arch (Figs. 2a-d, 3e-g, 5e,f, Supplementary Fig. 2, 3) is outstandingly well preserved. The cleaver-shaped 17 palatoquadrate has the high otic process and low palatine process 1,28 common to many early sharks. Otic and palatine portions each account for around half of the total length. A narrow, semi-elliptical surface on the leading edge of the otic process (Fig. 3f) articulates with the postorbital process and arcade of the neurocranium. The external surface of the quadrate and otic portion of the palatoquadrate is strongly concave, forming a broad and deep attachment space for the adductor (quadratomandibularis) muscles. The medial surface is correspondingly convex, but with a gently rounded, oblique ridge marking the ventral boundary of an area that might have been occupied by the spiracular pouch (cf. Brazeau & Ahlberg 29). The otic process rim is prominent throughout all of the posterior and most of the dorsal boundary, thinning-out only at the anterodorsal extremity, just below a ridge and groove that likely supported a mandibular branch of nerve VII (Fig. 3). In lateral aspect (Fig 3f, g), the sigmoid ventral margin of the palatoquadrate is concave downward in the quadrate region and convex downward in the palatine (cf. *Orthacanthus*, 30). In dorsal view the palatine process is mediolaterally broad, forming a substantial portion of the orbit floor (Fig. 2c). The ventral surface bears a shallow dental trough (Fig. 3e, Supplementary Fig. 2c), divided into about nine concavities for generative tooth sets. Anteriorly, a slight additional medial expansion of palatine process bears a ridge and groove articulation with the suborbital process of the neurocranium. The primary articulation with Meckel's cartilage, the quadrate condyle, is at the posterolateral extremity of the palatoquadrate (Fig. 3e, Supplementary Fig. 3). The secondary, medial articulation, the glenoid recess for the mandibular knob or process of Meckel's cartilage, is offset both dorsally and anteriorly (Supplementary Fig. 3). The axis connecting these two surfaces subtends an angle of about 45 degrees relative to the long axis

of Meckel's cartilage, and slopes dorsomedially to ventrolaterally at an angle of 45 degrees relative to a horizontal plain connecting left and right quadrates (Fig. 3e).

Comment [WU5]: plane

The lateral, external surface of Meckel`s cartilage (Fig. 3f) is deeply concave for the posterior two thirds of its length, providing a space for adductor muscle insertion. Dorsally, the laterally expanded margin (anterior to the adductor recess) forms a platform for the dentition. The platform is narrow anteriorly but broadens posteriorly: the breadth is considerable (Supplementary Fig. 3), unlike narrower dental platforms present in taxa such as xenacanths 31. Like the palatoquadrate, the dental trough is divided into about nine shallow concavities (Fig. 5c, e,f). A slight posterior rise of the dental platform resembles a coronoid process, but this is simply the posterior limit of the gently concave dental platform, matching the convexity of the corresponding palatine process. The articular region of the lower jaw is situated on the dorsal margin at the posterior extremity (Fig. 5e,f, Supplementary Fig.1). The chief articular facet is a posterolateral concavity, the articular cotylus, which receives the articular process of the palatoquadrate. Anterior to the articular cotylus, the mesial margin is produced into a strong, dorsally directed mandibular knob (Fig. 3g, Supplementary Fig. 3), and this is received by the articular cotylus of the palatoquadrate. As for the upper jaw, the axis (of rotation) of this hinge lies at an angle of about 45° relative to the long axis of the lower jaw. The medial surface of the mandible is generally convex, except of the posteroventral margin, which forms a smooth concavity. In dorsal view, the long axis of Meckel's cartilage is remarkably straight, and shows none of the characteristic curvature evident in the lower jaws of *Tristychius* 21, *Gogoselachus* 32, or xenacanths 30,31. The anterior terminus of the mandible is slender, rounded and shallow, signalling the absence of any substantial, stable symphysial connection: the two halves of the lower jaw meet more or less point-to-point.

The dentition is exposed but damaged (Supplementary Fig. 4). It consists of numerous, small, symmetrical, cladodont teeth, the largest observable bases of which are barely 2 mm

across. Each tooth bears a prominent median cusp flanked by diminutive slender lateral cusps; broken sections through some of the larger tooth bases suggest the presence of intermediate cusps.

Like *Ozarcus*, 16 the hyoid arch includes paired hyomandibulae, ceratohyals, and hypohyals (Figs 2a-c, 3e, g). Each hyomandibula is gently curved, anteriorly expanded and laterally compressed. The shape matches that of the Tennessee cladoselachian 33 rather than the more linear outline of the *Ozarcus* hyomandibula, and extends forward to just behind the orbit. However, unlike *Ozarcus*, the hyomandibula meets the ceratohyal directly posterior and medial to the articulation of the mandibular arch.

The ceratohyal is slender and elongate, with a dorsally curved posterior process resembling a walking stick handle (Fig. 3g, Supplementary Fig. 5). This 'handle' fits snugly within a shallow recess between the articular surfaces at the posterior of Meckel's cartilage, from which it ascends to meet the hyomandibula. The ceratohyal-hyomandibula articulation aligns, dorsoventrally, with the apex of the mandibular knob of Meckel's cartilage: i.e., level with the secondary articulation of the mandibular arch. There is a deep fossa in the posterior part of the external surface of the ceratohyal, at the point of maximum dorsal curvature (Supplementary Fig. 5). The ventrolateral margin of the fossa is extended laterally to form a gently convex flange or process that fits neatly within a matching recess in the ventromedial surface of Meckel's cartilage (Figs 1c, 2a). The hypohyal is simple, short, cylindrical and directed anteriorly. There is no trace of a basihyal.

Five gill arches are preserved, including an apparently complete set of ceratobranchials from left and right sides (Fig. 2a-c). Epibranchials, too, include up to five members. All paired cartilages of the gill skeleton are simple rods. There are no remains of hypobranchials or pharyngobranchials. The basibranchial series is represented by a broad posterior copula 34, somewhat like that of *Gutturensis* 27.

The scapulocoracoid (Fig. 2, Supplementary Fig. 6) resembles those of other symmoriiform chondrichthyans 1,10. The flat scapula blade has a well-developed anterior process at the dorsal apex, but the posterolateral process, although broken, appears rounded. The ventral part of the scapula is mediolaterally broad as it blends into the roof of the articular surface for the pectoral fin. In ventral view, the base of the scapulacoracoid appears triangular and its posterior portion shows a concavity for the articulation with the proximal radials of the pectoral fin. The coracoid region is convex anteriorly and concave posteriorly. A procoracoid has not been detected, although present in other symmoriids 5,10.

The pelvic girdle is known only from a small, simple triangular plate visible in the posterior of a pyrite concretion, which is located near the middle of the body (Fig. 1b, e). In other symmoriiform chondrichthyans such as *Akmonistion*, *Cobelodus*, *Denaea,* and *Symmorium*, the pelvic plate varies from subtriangular to oval 1,10.

A dorsal fin spine is preserved at the level of the pectoral girdle (Fig. 2, Supplementary Fig. 6f). The fin spine resembles those of cladoselachians 1,35 in having a strong, caudally recurved dorsal apex and smooth surface bearing no ridges or tubercles. However, the overall shape and proportions of the spine are considerably narrower and longer than cladoselachian examples in lateral view. Dorsal fin conditions are unknown.

Phylogenetic significance. Results of phylogenetic analysis place the symmoriiform sharks, including *Ferromirum*, as a clade branching from the holocephalan stem, consistent with recent and related results 3,13,14,36 (Fig. 4). Changes are mostly confined to the chondrichthyan stem branching pattern. Notably, *Gladbachus* no longer branches from close to the base of the total group 13, but is instead sister group to *Pucapampella* plus *Gydoselache*, *Doliodus*, and crown chondrichthyans, corroborating results found by Dearden *et al*. 36. The structure of the chondrichthyan crown is reasonably robust, and signals from data partitions are mostly consistent with the overall result. Exclusion tests limiting the

characters to neurocranial conditions (characters 1-4; 100-180) recover the branching structure of the chondrichthyan crown obtained from the complete data set (Adams consensus). Similarly, symmoriiforms emerge as stem-holocephalans in searches excluding neurocranial characters. However, in these trees, *Squalus* is the immediate sister of the holocephalan total-group, and putative stem-elasmobranchs (identified in analyses of the complete data set) are excluded from the chondrichthyan crown. Nevertheless, these putative stem-elasmobranchs persist in forming a monophyletic clade that branches from the chondrichthyan stem apex, and this result reoccurs in trees obtained from characters restricted to scales (characters 5-48), teeth (characters 65-86), and fin spines (characters 215-230).

Relationships within the symmoriiforms are unstable, and all resolution is lost in a consensus of tree lengths of only one extra step. Even so, the nested position of *Ferromirum* suggests that multiple symmoriiform lineages extended back into the Late Devonian (Fig. 4). Notably, this pattern is found throughout the major divisions of early members of the chondrichthyan crown-clade, and our results imply that much of the diversity of late Palaeozoic Chondrichthyes results from cladogenic events occurring before the Devonian-Carboniferous boundary.

Anatomical significance. The exceptional 3D-preservation of the jaws and hyoid arch of *Ferromirum* provides new insights into early chondrichthyan cranial structure and function. Initially using computer models with subsequent assembly of a physical model derived from STL files, we found that the jaws and hyoid apparatus could be rescaled to achieve a remarkably precise fit to the undistorted 3D-neurocranium of the much younger, early Permian symmoriiform *Dwykaselachus oosthuizeni* 3 (Fig. 5a-d, Supplementary Fig. 3, 7, 8). This result both tests and corroborates an implicit assumption that the articulations between the neurocranium and the mandibular plus hyoid arches barely changed throughout the history of the Symmoriiformes. Moreover, this striking morphological conservatism spanning a ~75

million-year age difference between these specimens, echoes genomic evidence of remarkably slow evolutionary rates within the holocephalan lineage relative to all other gnathostome clades 37.

Importantly, in both *Ferromirum* as preserved (Figs 1, 2) and in the *Ferromirum-Dwykaselachus* composite model (Fig. 5), the hyomandibula-ceratohyal joint is directly medial to the mandibular arch joint. This is quite unlike the recent interpretation of *Ozarcus* 16, where the joints and arches are spaced one behind the other. Thus, unlike *Ozarcus*, *Ferromirum* includes no space for an enlarged pseudobranch-bearing spiracular pouch or fully respiratory gill pouch. The precise fit between the flanged rear of the ceratohyal and the ventromedial profile of Meckel's cartilage shown in Figure 1c is also present in an undescribed specimen of *Cobelodus aculeatus* (FMNH PF 7351) in the Field Museum (Chicago, IL) collection of sharks from the Mecca fauna of the Pennsylvanian black shales of central North America 5,6. This specimen, like *Ferromirum*, is preserved in a barely disturbed posture with the ventral surface exposed. Comparison with *Chlamydoselachus* suggests that the ceratohyal flange provides the origin of the mandibulo-ceratohyal ligament 38. Taken together, these data strongly imply that the apparent gap between mandibular and hyoid arches in *Ozarcus* and *Cobelodus* 5,16,39 is an artefact of post-mortem lateral compression. Furthermore, rather than having a non-suspensory function, the symmoriiform hyoid arch is specialized and intimately involved in the jaw mechanism, *contra* the tenets of the aphetohyoidean hypothesis and related scenarios of visceral arch evolution 1,40-42.

Comment [WU6]: Why is a comparison with a crown-group elasmobranch relevant?

Functional morphology of the mandibular and hyoid arches. The physical model (Fig. 5; Supplementary Fig. 8), combining hyoid and mandibular arches of *Ferromirum* and the braincase of *Dwykaselachus*, allowed direct investigation of jaw and hyoid arch motion. The amphistylic 43, or, rather, archaeostylic 44 mode of jaw suspension includes an articulation between the leading edge of the palatoquadrate otic process and the convex rear of the postorbital arcade, and between the palatoquadrate palatine ramus and the orbital articulation. The sigmoid profile of the jaw joint (Fig. 6a) forms a hinge, but, as noted in the description, the rotational axis between primary and secondary articulations is offset relative to the cardinal axes of the cranium (Supplementary Fig. 3). The resulting jaw motion seems counter-intuitive. Meckel's cartilage rolls laterally (eversion: biting surface outwards) along its long axis while the jaw is opening, and medially (inversion: biting surface inwards) as the jaw closes (Fig. 6a,b). There is no broad mandibular symphysis to restrict this rotation during jaw depression and elevation (Fig. 5b). At a gape of 60 degrees, each side of the lower jaw has rolled outwards by approximately 20 degrees relative to its orientation when the jaws are closed (Fig. 6b), and the span of left and right mandibles in ventral view is slightly narrowed: the symphysial angle reduced from 50 (jaws closed) to 45 degrees (jaws open). Thus, as the gape opens, the dentition is everted and a greater proportion of the tooth battery is presented to the surrounding water column and prey. Comparison with analysis of tooth whorl function in *Helicoprion* 45 suggests that older teeth, closer to being shed or displaced onto the lateral surface of the jaw, would have a slighter greater velocity advantage as the jaws close and the dentition is rolled inwards. Such teeth are likely to impale or snag prey before such items are pushed or drawn into the mouth. Lingually located younger and larger teeth might also became functional within the bite because of this rotation. Dental batteries of left and right sides would be rotated medially through a combined angle of 40 degrees (Fig. 6c), scooping material into the gape where the younger, newer, teeth, deeper in the jaws with sharper and larger crowns, could be used to puncture and push prey deeper into the buccal space.

Comment [WU7]: Is this also related to the large jaw platform described above?

Comment [WU8]: Do you see any evidence of tooth root fusion that would prevent tooth shedding?

Comment [WU9]: Can you expand upon this? It's not clear what you mean. Maybe discuss this velocity advantage more fully in Helicoprion?

The role of the hyoid arch is not completely understood, but it is clear that it fits closely within the gently recessed rear of the palate and mandible. The archaeostylic palate might be considered self-supporting, but the hyomandibula likely served as an important structural brace limiting dorsoventral movement. Furthermore, manipulation of the model reveals that slight raising of the distal end of the hyomandibula contributes to depression of the ceratohyal and mandible.

Comment [WU10]: Does it also contribute to the lateral movement of the jaws as the ceratohyal is depressed? Or a brace for this movement?

To the best of our knowledge, the pattern of jaw motion described here is unknown in living fishes sensu lato, although a hemimandibular roll is considered crucial to oral processing in early mammals 19. Among Palaeozoic sharks, *Ferromirum* is quite unlike the specialized saw-jawed stem-holocephalan *Helicoprion* 45 and the suction feeding stem-elasmobranch *Tristychius* 21. However, key aspects of the feeding apparatus in *Ferromirum* are widespread among early chondrichthyans 1,2,5-8: a slender ceratohyal, an elongate hyomandibula, a jaw joint far behind the orbit (at the extremity of a cleaver-shaped palate), and no trace of labial cartilages. For these reasons, we suggest that *Ferromirum* likely provides a glimpse of more general functional conditions in early sharks. Notably, in the Maïder Basin, *Ferromirum* is one of several chondrichthyans occurring within sediments rich in thylacocephalan crustaceans 22,46 (Fig. 7). It appears likely that ram feeding, employing a large gape lined with generative sets of cladodont teeth, with those of the lower jaw rotating symmetrically inwards as the mouth snaps shut, would provide an effective means of capturing and retaining such seemingly abundant invertebrate prey.

Preservation quality has limited previous studies of early chondrichthyan jaw mechanics 44 but insights from exceptionally preserved 3D-material might be used to identify morphological correlates of similar function among richer collections of flattened specimens. 2D-jaw shape measures of biomechanical traits have already been used to estimate functional disparity and implied ecological variety across early gnathostomes 47. Thus, from the present work, the location of a mesial process (mandibular knob) relative to the primary articulation

surface might be used similarly as a predictor of 3D jaw motion; especially hemimandibular long-axis rotation if also associated with a slender mandibular symphysis. In this regard, the jaws of symmoriiforms such as *Denaea* and *Symmorium* 6 closely match those of *Ferromirum*. Moreover, the mandibular mesial process is a well-established chondrichthyan synapomorphy 47 occurring deep within the stem lineage. It would be interesting to learn how the distribution of this process, and specialized jaw hinge, correlates with the evolution of the classic, tooth-whorl dominated, shark dentition.

Methods

Studied material. The type and only specimen (PIMUZ A/I 4806) of *Ferromirum oukherbouchi* gen. et sp. nov. is housed at the Palaeontological Institute and Museum of the University of Zurich, Switzerland. The specimen was prepared out of a ferruginous reddish nodule (rich in haematite) from the Famennian (Late Devonian) of Madene El Mrakib in the Maïder region of the southeastern Anti-Atlas (Morocco).

Phylogenetic analysis. We performed a heuristic search in PAUP* 4.167 49 using a parsimony ratchet 50 with an initial 10,000 random sequence additions. The character matrix consists of 64 taxa, 56 ingroup and eight outgroup taxa, coded for 230 morphological characters. The analysis recovered 90 equally most parsimonious trees (MPTs) of 535 steps (consistency index 0.46; retention index 0.78; RC 0.36). We assessed nodal support through bootstrapping and Bremer Decay indices. Please see Supplementary Note 1 for further details.

X-ray micro-tomography. High resolution data were obtained using an industrial computed tomography scanner (Nikon XT H 225 ST) at the University of Zurich, Switzerland. Data acquisition and image reconstruction parameters are: 224 kV, 474µA; filter: 4 mm of copper; isotropic voxel dimensions of 0.091 mm; 16-bit TIFF images were acquired; XX 8-bit TIFF

images were used for reconstruction. Images were analysed and 3D models reconstructed using Mimics v.17 (http://www.biomedical.materialise.com/mimics; Materialise, Leuven, Belgium). The 3D-object was edited (smoothing, colours and lightning) in MeshLab v. 2016 (http://www.meshlab.net; 41) and blender v2.79b (https://www.blender.org; Amsterdam, Netherlands).

Virtual and physical models. 3D anatomical reconstructions of *Ferromirum* palatoquadrate, Meckel`s cartilage, hyomandibula and ceratohyal, and *Dwykaselachus* neurocranium used Mimics v. 18 (biomedical.materialise.com/mimics; Materialise, Leuven, Belgium) for three-dimensional modelling, STL polygon creation and kinematic simulations. Further editing of the STLs (colour, texture, lighting), kinematics and mirroring for the final restorations (virtual images, virtual manipulations, and 3D printouts of STL files) used 3D Studio Max (Autodesk.com/products/3ds-max; Autodesk, San Rafael, USA).

Nomenclatural acts. This published work has been registered in ZooBank, the proposed online registration system for the International Code of Zoological Nomenclature (ICZN). The ZooBank LSIDs (Life Science Identifiers) can be resolved and associated information viewed through any standard web browser by appending LSID to the prefix "http://zoobank.org/." The LSIDs for this publication are: zoobank.org.pub:XXXXXXXX

Reporting summary. Further information supporting the results and discussion of this study is available in the Nature Research Reporting Summary linked to this article.

Data availability

The authors declare that all data supporting the findings of this study are available at Dryad XX.

References

1. Zangerl, R. *Handbook of Paleoichthyology volume 3A: Chondrichthyes I, Paleozoic Elasmobranchii.* (ed. Schultze, H.P) (Gustave Fischer, 1981).
2. Janvier, P. *Early Vertebrates* (Clarendon, 1996).
3. Coates, M. I., Gess, R. W., Finarelli, J. A, Criswell, K. E, & Tietjen, K. A symmoriiform chondrichthyan braincase and the origin of chimaeroid fishes. *Nature* **541**, 209-211 (2017).
4. Guinot, G., Adnet, S., Cavin, L. & Cappetta, H. Cretaceous stem chondrichthyans survived the end-Permian mass extinction. *Nat. Comm.* **4**, 2669 (2013).
5. Zangerl, R. & Case, G. R. *Cobelodus aculeatus* (Cope), an anacanthous shark from Pennsylvanian Black Shales of North America. *Palaeontographica A* **154**, 107–57 (1976).
6. Williams, M. E. The ‘‘cladodont level’’ sharks of the Pennsylvanian Black Shales of central North America. *Palaeontographica A* **190**, 83–158 (1985).
7. Lund, R. The morphology of *Falcatus falcatus* (St. John and Worthen), a Mississippian stethacanthid chndrichthyan from the Bear Gulch Limestone of Montana. *J. Vert. Paleontol.* **5**, 1-19 (1985).
8. Lund, R. On *Damocles serratus* nov. gen. et sp., (Elasmobranchii: Cladodontida) from the Upper Mississippian Bear Gulch Limestone of Montana. *J. Vert. Paleontol.* **6**, 12-19 (1986).
9. Coates, M. I., Sansom, I. J., Sequeira, S. E. K. & Smith, M. M. Spines, teeth and histology: novel structures and tissues in ancient sharks. *Nature* **396**, 729-730 (1998).
10. Coates, M. I. & Sequeira, S. E. K. A new stethacanthid chondrichthyan from the Lower Carboniferous of Bearsden, Scotland. *J. Vertebr. Paleontol.* **21**, 754–766 (2001).

11. Pradel, A., Tafforeau, P., Maisey, J. G. & Janvier P. A new Paleozoic Symmoriiformes (Chondrichthyes) from the Late Carboniferous of Kansas (USA) and Cladistic Analysis of Early Chondrichthyans. *PLoS ONE* **6**: e24938 (2011).
12. Maisey, J. G., Miller, R., Pradel, A., Denton, J. S. S., Bronson A. & Janvier, P. Pectoral morphology in *Doliodus*: bridging the 'acanthodian'-chondrichthyan divide. *Am. Mus. Novit*. **3875**, 1-15 (2017).
13. Coates, M. I., Finarelli, J. A., Sansom, I. J., Andreev, P. S., Criswell, K. E, Tietjen, K., Rivers, M. L. & La Riviere, P. J. An early chondrichthyan and the evolutionary assembly of a shark body plan. *Proc. R. Soc. Ser. B.* **285**: 20172418 (2018).
14. Frey, L., Coates, M. I., Ginter, M., Hairapetian, V. Rücklin, M., Jerian, I. & Klug, C. The early elasmobranch *Phoebodus*: phylogenetic relationships, ecomorphology, and a new time-scale for shark evolution. *Proc. R. Soc. Ser. B.* **286**: 20191336 (2019).
15. Maisey, J. G. The braincase in Paleozoic symmoriiform and cladoselachian sharks. *Bull. Am. Mus. Nat. Hist*. **307**, 1-122 (2007).
16. Pradel, A., Maisey, J. G., Tafforeau, P., Mapes, R. H. Mapes & Mallatt, J. A Palaeozoic shark with osteichthyan-like branchial arches. *Nature* **509**, 608-611 (2014).
17. Schaeffer B. In *Problèmes actuels de paléontologie, évolution des vertébrés*. (ed. Lehman, J. P.) 101-109 (Colloque Internationaux CNRS, 1975).
18. Motta, P. J. & Huber, D. R. In *Biology of sharks and their relatives*, (eds. Carrier, J. C., Musick, J. A., Heithaus, M. R.) 153-209 (CRC Press, 2012).
19. Bhullar, B-A. S., Manafzadeh, A. R., Miyamae, J. A., Hoffman, E. A., Brainerd, E. L., Musinsky, C. & Crompton, A. W. Rolling of the jaw is essential for mammalian chewing and tribosphenic molar function. *Nature* **566**, 528-532. (2019)
20. Brazeau, M. D. & Friedman, M. The origin and early phylogenetic history of jawed vertebrates. *Nature* **520**, 490-497 (2015).

21. Coates, M. I., Tietjen, K., Olsen, A. M. & Finarelli, J. A. High-performance suction feeding in an early elasmobranch. *Sci. Adv*. 2019; **5**: eaax2742 (2019).

22. Frey, L., Rücklin, M., Korn, D. & Klug, C. Late Devonian and Early Carboniferous alpha diversity, ecospace occupation, vertebrate assemblages and bio-events of southeastern Morocco. *Palaeogeogr. Palaeoclimatol. Palaeoecol.* **496**, 1-17 (2018).

23. Frey, L., Pohle, A., Rücklin, M. & Klug, C. Fossil-Lagerstätten and preservation of vertebrates and invertebrates from the Devonian of Morocco (eastern Anti-Atlas). *Lethaia*, 25 pp, https://doi.org/10.1111/let.12354 (2019).

24. Huxley, T. *A manual of the anatomy of vertebrated animals*. (D-Appleton and Co., 1880).

25. Bonaparte C. L. J. L. *Iconografia della fauna italica per le quatro classi degli animali vertebrati. Tomo III: Pesci*. (Salviucci, 1838).

26. Dean, B. Studies on fossil fishes (sharks, chimaeroids and arthrodires. *Memoirs of the AMNH* **9(5)**, 209-287 (1909C).

27. Sequeira, S. E. K. & Coates, M. I. Reassessment of *'Cladodus' neilsoni* Traquair: a primitive shark from the Lower Carboniferous of East Kilbride, Scotland. *Palaeontology* **43**, 153-172 (2000).

28. Schaeffer B. The xenacanth shark neurocranium, with comments on elasmobranch monophyly. *Bull. Am. Mus. Nat. Hist.* **169**, 1–66 (1981).

29. Brazeau, M. D. & Ahlberg, P. E. Tetrapod-like middle ear architecture in a Devonian fish. *Nature* **439**, 318-321 (2006).

30. Hotton, N. Jaws and teeth of American xenacanth sharks. *J. Paleontol.* **26**, 489-500 (1952).

31. Hampe, O. Revision of the Xenacanthida (Chondrichthyes: Elasmobranchii) from the Carboniferous of the British Isles. *Earth Environ. Sci. Trans. R. Soc. Edinb.* **93**, 191-237 (2002).

32. Long, J. A., Burrow, C. J., Ginter, M., Maisey, J. G., Trinajstic, K. M., Coates, M. I. et al. First shark from the Late Devonian (Frasnian) Gogo Formation, Western Australia sheds new light on the development of tessellated calcified cartilage. *PLoS ONE* **10**, e0126066 (2015).

33. Maisey, J. G. Visceral skeleton and musculature of a Late Devonian shark. J. *Vertebr. Paleontol.* **9**, 174-190 (1989).

34. Nelson, G. J. Gill arches and the phylogeny of fishes, with notes on the classification of vertebrates. *Bull. Am. Mus. Nat. Hist.* **141**, 475–552 (1969).

35. Harris, J. E. The dorsal fin spine of *Cladoselache*. *Sci. publ. Clevel. Mus. Nat. Hist.* **8**, 1-6 (1938).

36. Dearden, R. P., Stockey, C. & Brazeau, M. D. The pharynx of the stem-chondrichthyan *Ptomacanthus* and the early evolution of the gnathostome gill skeleton. *Nat. Commun*. **10**, 2050. https://doi.org/10.1038/s41467-019-10032-3 (2019).

37. Venkatesh, B. *et al.* Elephant shark genome provides unique insights into gnathostome evolution. *Nature* **505**, 174-179 (2014).

38. Allis, E. P. The cranial anatomy of *Chlamydoselachus anguineus*. *Acta Zool*. **4**, 123-221(1923).

39. Zangerl, R. & Williams, M. E. New evidence of the nature of the jaw suspension in Palaeozoic anacanthous sharks. *Palaeontology* **18**, 333-341 (1975).

40. Gegenbaur, C. *Untersuchungen zur vergleichenden Anatomie der Wirbelthiere. III. Das Kopfskelet der Selachier, ein Beitrag zur Erkenntniss der Genese des Kopfskeletes der Wirbelthiere* (Engelmann, Leipzig, 1872).

41. De Beer 1937 G. R. DeBeer, *The Development of the Vertebrate Skull* (Univ. Chicago, 1937).

42. Mallat, J. Ventilation and the origin of jawed vertebrates: a new mouth. *Zool. J. Linn. Soc.* **117**, 329-404 (1996).

43. Huxley, T. H. Contributions to morphology. Ichthyopsida. No 1. On *Ceratodus forsteri*, with observations on the classification of fishes. *Proc. Zool. Soc. London* **1876**, 24-59 (1876).

44. Maisey, J. G. The postorbital palatoquadrate articulation in elasmobranchs. *J. Morphol.* **269**, 1022-1040 (2008).

45. Ramsay, J. B., Wilga, C. D., Tapanila, L., Pruitt, J., Pradel, A., Schlader, R. & Didier, D. A. Eating with a saw for a jaw: functional morphology of the jaws and tooth-whorl in *Helicoprion davisii*. *J. Morphol.* **276**, 47-64 (2015).

46. Jobbins, M., Haug, C. & Klug, C. First African thylacocephalans from the Famennian of Morocco and their role in Late Devonian food webs. *Sci. Rep.* **10**, 5129 (2020).

47. Anderson, P. S. L., Friedman, M., Brazeau, M. D. & Rayfield, E. J. Initial radiation of jaws demonstrated stability despite faunal and environmental change. *Nature* **476**, 206-209 (2011).

48. Davis, J.A. Finarelli, M.I. Coates, *Acanthodes* and shark-like conditions in the last common ancestor of modern gnathostomes. *Nature* **486**, 247-250 (2012).

49. Swofford, D. L. *PAUP*: Phylogenetic Analysis Using Parsimony* (*and other methods). (Sinauer Associates, 2003).

50. Quicke, D. L. J., Taylor, J. & Purvis, A. Changing the landscape: a new strategy for estimating large phylogenies. *Syst. Biol.* **50**, 60-66.

Acknowledgements

We are deeply indebted to Saïd Oukherbouch (Tafraoute, Morocco) who discovered the holotype and who supported us during field work. The holotype was prepared by the team of the Tahiri Museum of Fossils and Minerals (Erfoud, Morocco). Anita Schweizer (Zurich) and Alexandra Wegemann (University of Zurich) kindly acquired CT-scans for us. Many thanks to Thodoris Argyriou (University of Zurich) who helped segmenting the image stack. Michal Ginter (Warsaw) helped with the interpretation of the teeth.

Author contributions: L.F.: segmentation of computer tomographs, preparing figures, drafting manuscript. C.K., M.C., L.F.: creating the project. C.K.: photography, illustrations, formatting. K.T.: segmentation of computer tomographs, preparing figures, 3D-printing. M.C.: phylogenetic analysis, assembly and interpretation of 3D-models, photography, preparing figures. All authors contributed to the text.

Funding: The project (number 200020_184894) was financially supported by the Swiss National Science Foundation. M.R. was supported by NOW Vidi grant no. 864.14.009.

Competing interests: The authors declare no competing interests.

Additional information

Supplementary Information is available for this paper at XX.

Correspondence and requests should be addressed to CK and MC.

Figures

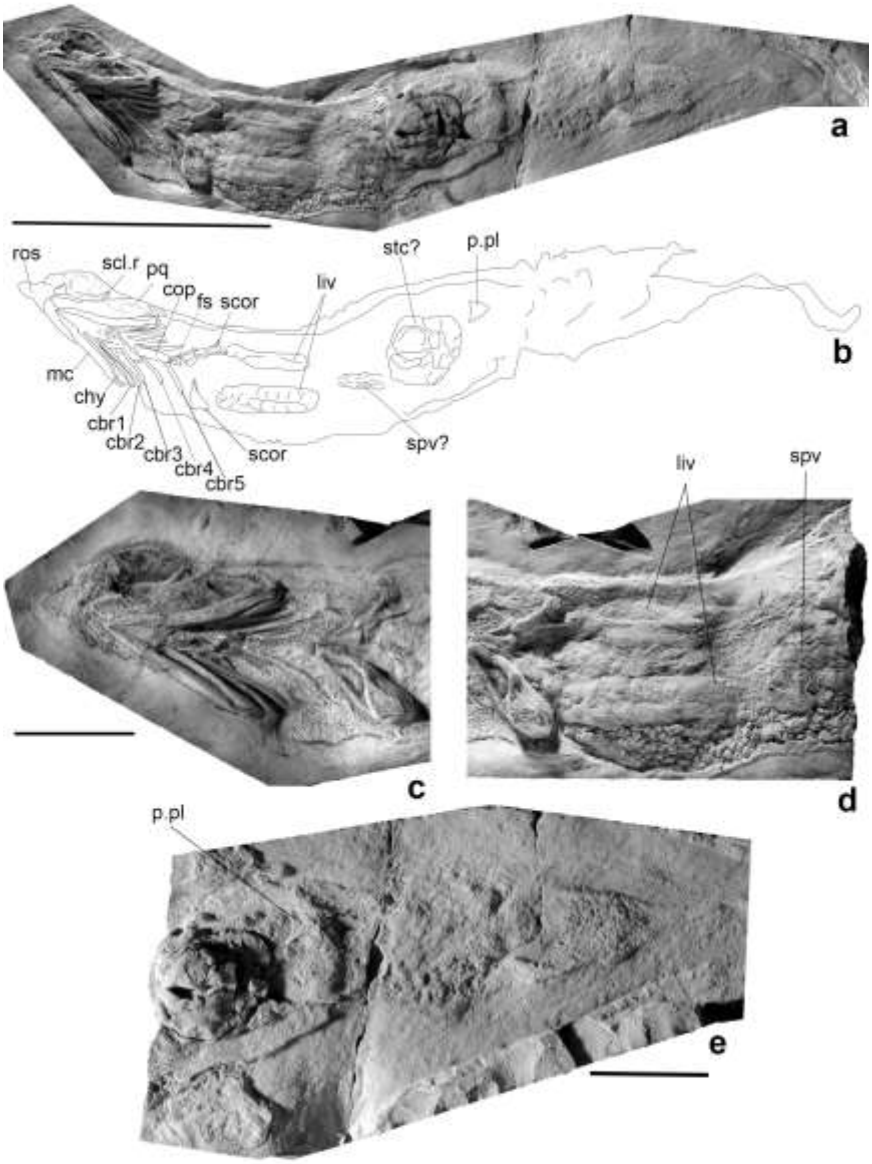

Fig. 1 *Ferromirum oukherbouchi* gen. et sp. nov., PIMUZ A/I 4806, early/ middle Famennian, Madene el Mrakib. **a** Photo and **b** line drawing of the specimen. **c** Head region including parts of the rostrum, sclerotic ring, mandibular arch, hyoid arch, branchial skeleton and shoulder girdle in ventral view. **d** Soft tissue remains including liver and spiral valves. **e** Pelvic and caudal region. Scale bars, 100 mm (**a**, **b**), and 30 mm (**c**-**e**). chy, ceratohyal; cop, copula; cbr, ceratobranchials; fs, fin spine; liv, liver; mc, Meckel`s cartilage; p.pl, pelvic plate; pq, palatoquadrate; ros, rostrum; scl.r, sclerotic ring; scor, scapulocoracoid; stc?, stomach content; spv, spiral valves.

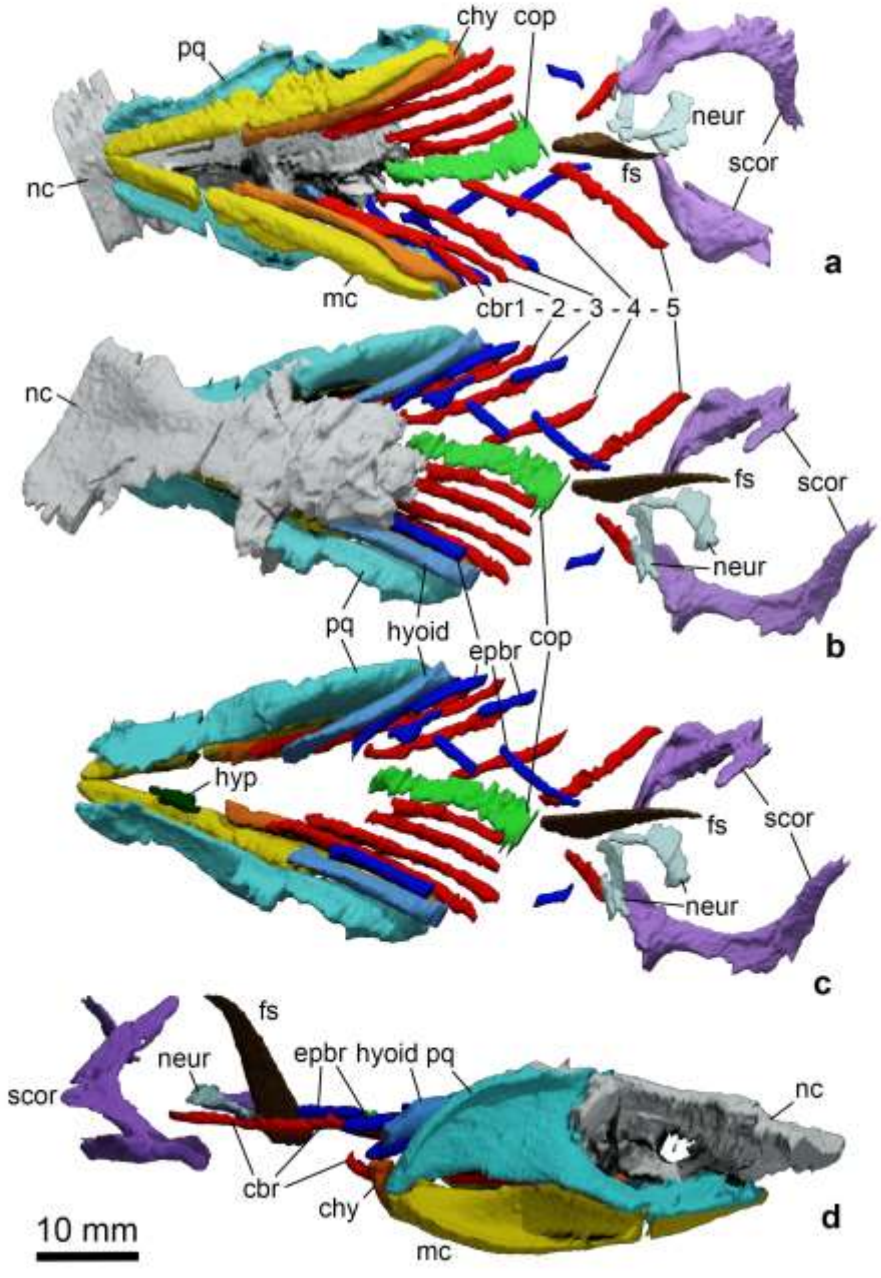

Fig. 2. *Ferromirum oukherbouchi* gen. et sp. nov. PIMUZ A/I 4806, virtual rendering of specimen based on CT-data showing the neurocranium, visceral arches, pectoral girdle and dorsal fin spine. **a** Ventral and **b** dorsal view with and **c** without braincase. **d** lateral view. Colour coding: grey, neurocranium (nc); turquoise, palatoquadrate (pq); yellow, Meckel's cartilage (mc); dark green, hypohyal (hyp); light blue, hyoid (hyoid); orange, ceratohyal (chy); blue, epibranchials (epbr); red, ceratobranchials (cpbr); green, copula (cop); brown, fin spine (fs); purple, pectoral girdle (scor); light turquoise, ? neural arches (neur).

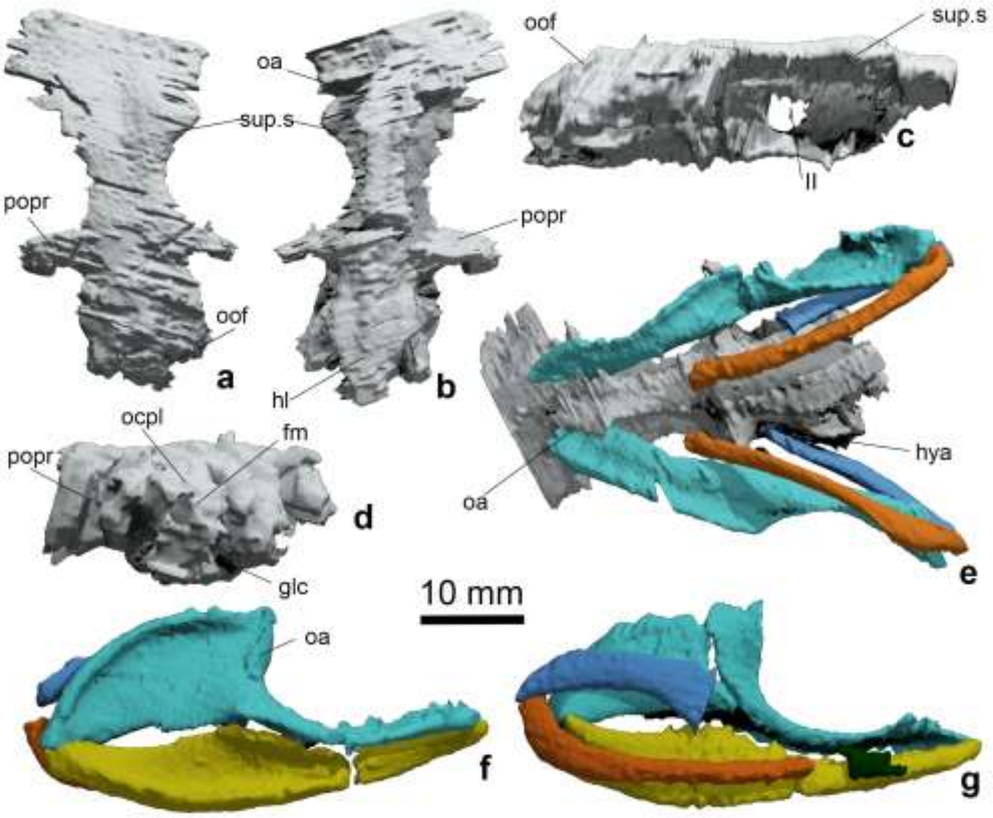

Fig. 3. *Ferromirum oukherbouchi* gen. et sp. nov., PIMUZ A/I 4806. Neurocranium in **a** dorsal, **b** ventral, **c** lateral, and **d** posterior, occipital views. **e** Articulation between braincase, palatoquadrate and hyoid arch in ventral view. **f**, **g** Arrangement of mandibular and hyoid arches in lateral and medial views respectively. Colour coding: grey, braincase; turquoise, palatoquadrate; yellow, Meckel's cartilage; dark green, hypohyal; light blue, hyoid; orange, ceratohyal. Note that the rostral roof includes an excess of poorly resolved cartilage or matrix left in place in the computer renderings. fm, foramen magnum; hl, hypotic lamina; hya, hyomandibular articulation; glc, glossopharyngeal canal; oa, orbital articulation; ocpl, occipital plate; oof, otico-occipital fissure; popr, postorbital process; sup.s, supraorbital shelf; **II**, optic nerve.

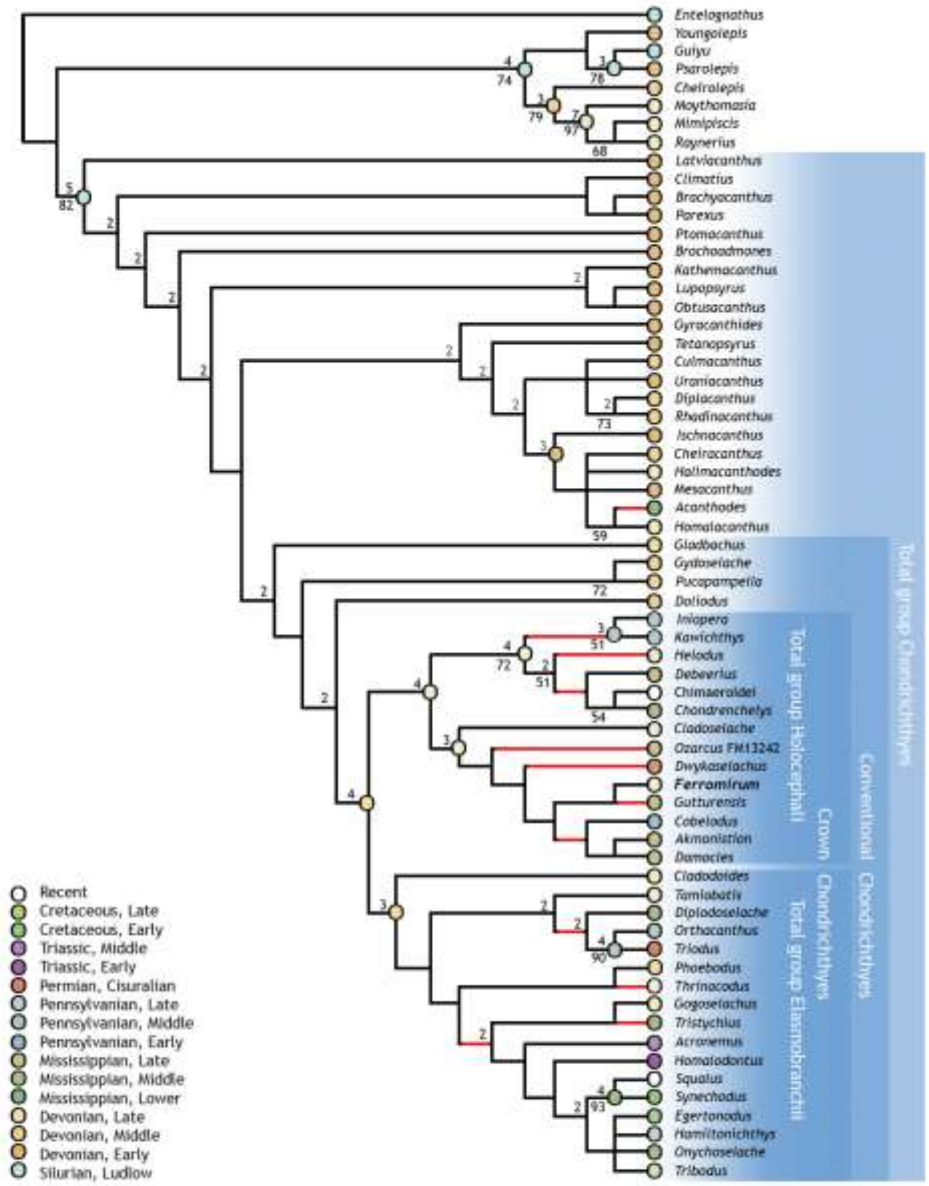

Fig. 4. Strict consensus of 90 trees showing phylogenetic affiliation of *Ferromirum* gen. nov. among early chondrichthyans. Bremer decay values above nodes; bootstrap support scores below. Circle colours (nodes and termini) denote earliest occurrence of taxon; red branches denote clade crossing Devonian-Carboniferous boundary.

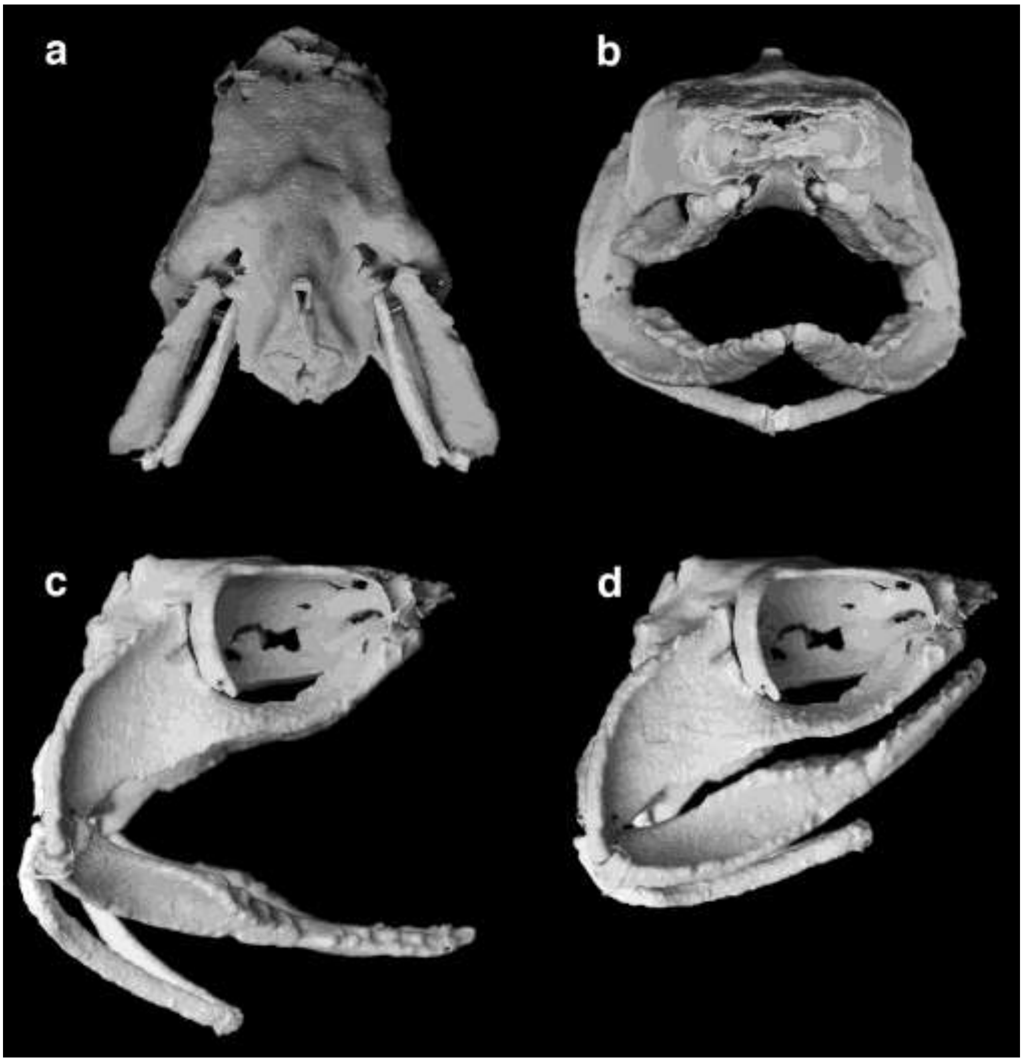

Fig. 5. Composite model of *Ferromirum* gen. et sp. nov. jaws and hyoid arch and *Dwykaselachus* neurocranium, in **a** dorsal, **b** anterior, **c** lateral with mandible and ceratohyal depressed, and **d** with mandible and ceratohyal raised.

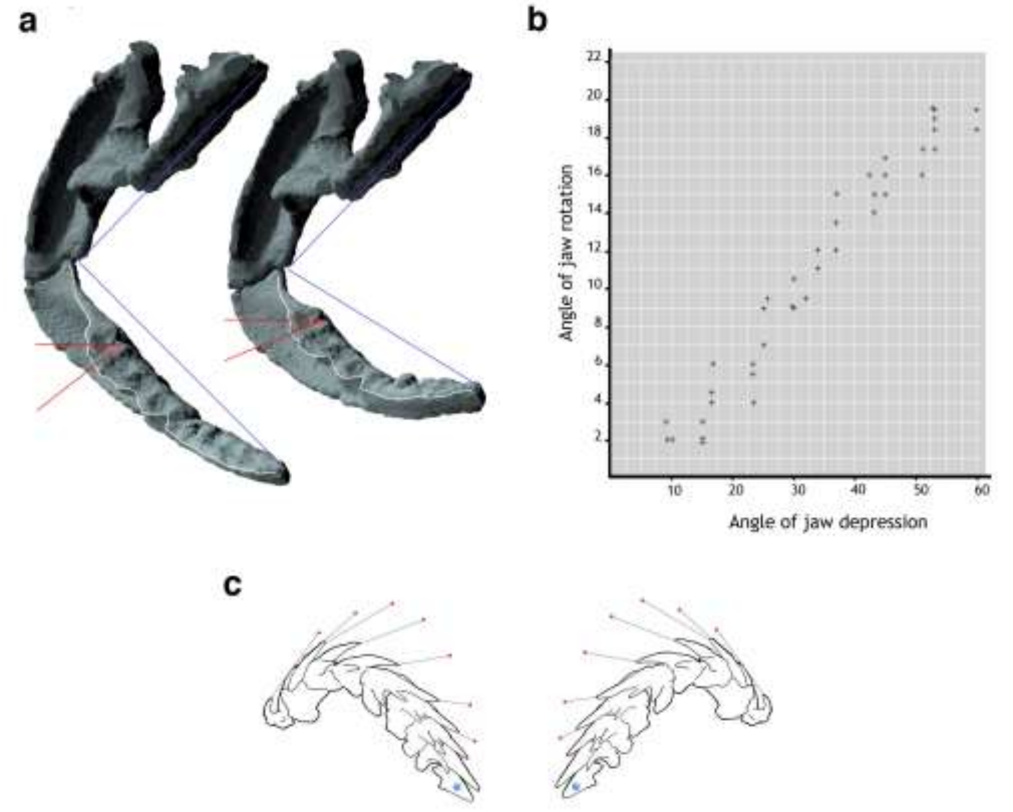

Fig. 6. **a** *Ferromirum* gen. et sp. nov. computer rendering of jaws in different degrees of Meckel's cartilage depression, in anterolateral view. **b** Graphed angle of Meckel's cartilage rotation relative to angle of depression, measurements taken from model in Fig. 5. **c** Line drawing of opposing lower jaw tooth sets, adapted from *Ozarcus* (16). Lines and red dots and mark displacement of crowns when rotated through 22 degrees (graphic convention adapted from ref. 45).

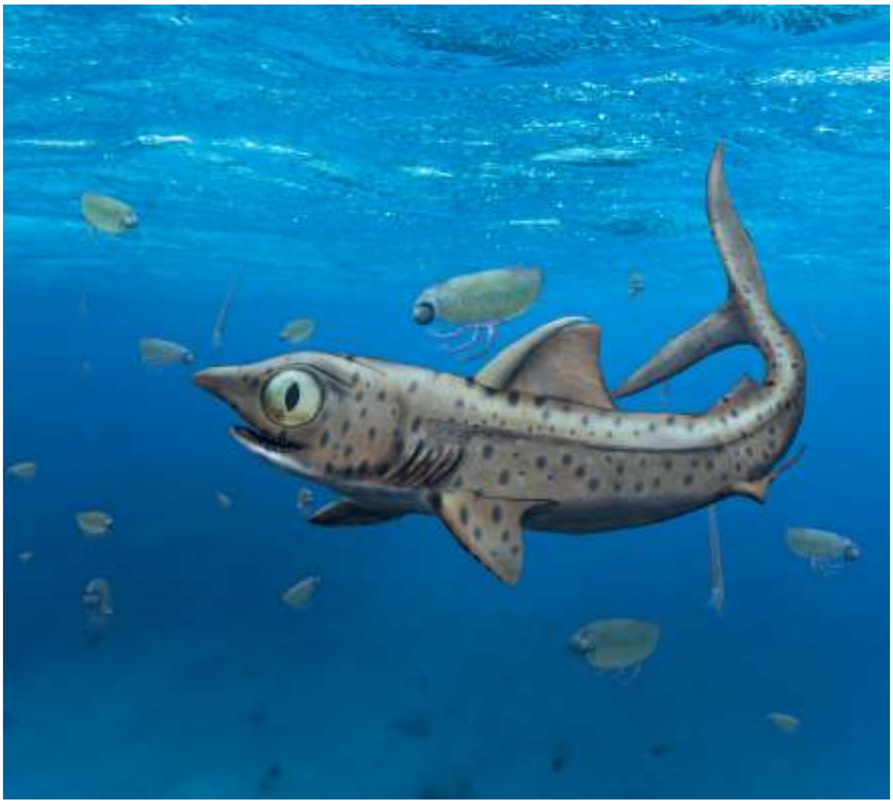

Fig. 7. Reconstruction of *Ferromirum oukherbouchi* gen. et sp. nov. and associated invertebrates (orthocerid cephalopods and thylacocephalans: *Concavicaris submarinus*) from the Famennian of the Maider region (Morocco).

A new symmoriiform from the Late Devonian of Morocco: novel jaw function in ancient sharks

L. Frey[1*], M. I. Coates[2], K. Tietjen[3], M. Rücklin[4] and C. Klug[1]

[1]Paläontologisches Institut und Museum, University of Zurich, Karl-Schmid-Strasse 4, CH-8006 Zürich (linda.frey@web.de; chklug@pim.uzh.ch).

[2]Department of Organismal Biology and Anatomy, University of Chicago, 1027 E. 57th St., USA-60637 Chicago.

[3]Biodiversity Institute, University of Kansas, 1345 Jayhawk Blvd, USA-66045 Lawrence.

[4] Naturalis Biodiversity Center, Postbus 9517, 2300 RA, Leiden, The Netherlands.

The fossil record of early chondrichthyans (sharks, rays, chimaeras and extinct relatives) and thus our knowledge of their anatomy and functional morphology are poor because of their predominantly cartilaginous skeletons. Here, we report a new symmoriiform shark, *Ferromirum oukherbouchi* gen. and sp.nov, from the Late Devonian of the Anti-Atlas. Computed tomography scanning reveals the undeformed shape of the jaws and hyoid arch, which are of a kind often used to represent primitive conditions for jawed vertebrates. Of critical importance, these closely fitting cartilages preclude the repeatedly hypothesized presence of a complete gill between mandibular and hyoid arches. We show that the jaw articulation is specialized and drives mandibular rotation outward when the mouth opens, and inward upon closure. The resultant eversion and inversion of the lower dentition presents a greater number of teeth to prey through the bite-cycle. This suggests an increased functional and

Comment [HBS1]: Rays and Chimaeras are not early chondrichtyans!
And ..according with the title …What is *Ferromirum a shark or an extint relative*….

I recommend rethink this unconcise first sentence. May be:
The fossil record of chondrichthyans (modern sharks, rays, chimaeras and their extinct relatives) is poor because of their predominantly cartilaginous skeletons.. Therefor, our knowledge of their anatomy and functional morphology is limited, moreover when we considered earlies representatives of the group.

Comment [HBS2]: Here and through all the mns.

Comment [HBS3]: Really?

ecomorphological disparity among chondrichthyan ~~jaws~~jaws preceding and surviving the end-Devonian extinctions.

The Symmoriiformes is a widely distributed group of early chondrichthyans ranging from the Late Devonian through to the early Permian, and perhaps extending as far as the Cretaceous 1-4. Like the vast majority of early ~~sharks~~chondrychtians, symmoriiforms have mostly been understood from two-dimensional skeletal and, more rarely, soft tissue remains, supplemented with insights from hard tissue histology 5-10. Symmoriiforms, occasionally including the classic Devonian genus *Cladoselache*, have often been used to exemplify early chondrichthyan conditions, and, from entrenched views of sharks as intrinsically primitive, generalised gnathostome conditions. Consistent with this treatment, both traditional and some of the more current phylogenetic hypotheses have placed this group on the chondrichthyan stem 11,12. However, an alternative series of analyses resolve symmoriiforms as stem holocephalans 2,3,10,13,14. Notably, the most recent of these employ the abundance of new data from X-ray tomography, especially concerning the neurocranium 3,11,15,16.

Comment [HBS4]: Just an observation. I have checked material of reference 4. Material is scarce and poor. Microestructure of enameloid its typical of euselachian teeth. I have serious doubts they belong to symmoriiformes.

Comment [HBS5]: These means the later hypothesis or both? (I think both, but please clarify)

Here, we describe a ~~recently discovered~~new symmoriiform shark from the Devonian of Morocco, analyse its systematic affinity, discuss the phylogenetic implications, and investigate the morphology and ~~motion~~ biomechanic of the superbly preserved jaws and hyoid arch. These jaws display the classic 'cleaver' shape 17 palate seen in a vast array of early crown-gnathostomes, and especially among chondrichthyans 2. However, in this unique specimen, the areas of attachment to the neurocranium and the jaw joint are undistorted, and likewise the slender cartilages of the hyoid arch. Crucially, these conditions allow a three-dimensional reconstruction of the feeding apparatus, and ~~show~~ suggest that the hyoid arch was functionally associated with the palate and mandible. Further to this, physical modelling of the jaws reveals hemimandibular long-axis rotation, an action thus far unrecorded in studies of the feeding mechanics of living chondrichthyans 18, although recently recognised

as an essential component in the evolution of mammalian feeding systems 19. These results add to new appreciation of chondrichthyans as early specialists in the gnathostome crown clade 20, and add to an emerging picture of functional disparity and likely ecomorphological partitioning among these early members of the modern vertebrate biota 21.

Results and discussion

~~**Stratigraphy.** Famennian, *Planitornoceras euryomphalum* to *Afrolobites mrakibensis* Zone; Ibâouane Formation, Lahfira Member, Thylacocephalan Layer (formerly described as Phyllocarid Layer 14,22,23, Madene el Mrakib, Maïder Basin, southeastern Anti-Atlas, Morocco.~~

Systematic palaeontology. Chondrichthyes Huxley, 1880 24

Total-group Holocephali Bonaparte, 1832 25

Symmoriiformes Zangerl, 1981 1

Ferromirum gen. nov.

Etymology: Derived from *ferrum* (lat. - iron) and *mirus* (lat. - miraculous). *Ferrum* refers to the preservation of the holotype in a reddish ferruginous nodule, which is characteristic for fossils from the Thylacocephalan Layer of the Maïder. *Mirus* refers to our initial misinterpretation of the gill remains of the holotype as crustacean appendages, before preparation and the miracle-like revelation that the specimen was, in fact, a chondrichthyan.

Type species: *F. oukherbouchi* sp. nov.

Diagnosis: A small symmoriid with slender body; head with short triangular rostrum and subterminal gape; supraorbital shelf with concave lateral margin; orbits large with sclerotic ring; narrow interorbital space; narrow suborbital shelf; cleaver-shaped palatoquadrate with anterolaterally directed articulation with postorbital process; scalloped margins of gape

Comment [HBS6]: Picture of ecomorphological disparity is also emerging early in jawless fishes:

Categorical versus geometric morphometric approaches to characterizing the evolution of morphological disparity in Osteostraci (Vertebrata, stem Gnathostomata)
Ferrón et al 2020
Patterns of ecological diversification in thelodonts
Ferrón et al 2018

Comment [HBS7]: All this paragraph seems part of the abstract of the work. I do not think" introduction" is the appropriate section to include it

Comment [HBS8]: If you describe a new taxon better include this as Type locality and horizon.
See below

suggest around nine upper and lower tooth families; slender ceratohyal with posteroventral lateral flange engaging with Meckel's cartilage; paired hypohyals anteriorly directed; no basihyal; distinguished from all other symmoriids by presence of pectoral-level, slender dorsal fin spine, smooth with posteriorly curved apex.

Ferromirum oukherbouchi sp. nov.

Etymology. The species name *oukherbouchi* honours the finder of the specimen Said Oukherbouch (Tafraoute).

Holotype. PIMUZ A/I 4806

Material. Holotype alone.

Locality. Madene El Mrakib, Maïder Basin, southeastern Anti-Atlas, Morocco.

Formation and age. Ibâouane Formation (middle Famennian, Upper Devonian), *Planitornoceras euryomphalum* to *Afrolobites mrakibensis* Zone.

Diagnosis. As for genus.

Description. The estimated body length of *Ferromirum oukherbouchi* gen. et sp. nov. is 330 mm. The specimen was prepared and exposed from its ventral side, revealing parts of the left orbit, mandibular, hyoid and branchial arches, pectoral and pelvic girdles (Fig. 1a-e; Supplementary Fig. 1). Substantial replacements of soft tissues are present throughout the body. Anteriorly, these reveal that the snout forms a short, pointed rostrum, resembling examples preserved in *Falcatus* and *Damocles* 7,8. As in these genera, there is no evidence of skeletal support for the rostral apex, which likely housed arrays of electro- and mechanoreceptive organs (possible remains are visible as minute circular pits on its venter; Supplementary Fig. 2i). The trunk region includes two elongate lobes of the liver (Fig. 1d), extending for perhaps 50% of the visceral cavity length. Part of the digestive tract is evident as a spiral valve, exposed in the midline between the caudalmost extremities of both liver

Comment [HBS9]: ???

Comment [HBS10]: Discussion or comparison, not description.
Also several sentences below-
I mark some in yellow but please check

lobes (Fig. 1a,b). Directly anterior to the pelvic plates, a large bolus of material might represent a mass of pre-rectal gut contents (Fig. 1a,b,e).

The computed tomograms reveal details of the braincase, jaws, hyoid arch, gill skeleton, pectoral girdle and dorsal fin spine (Figs. 2, 3, Supplementary Fig. 2i). However, the radiographic contrast between calcified cartilage and the surrounding matrix is often poor. The general shape of the neurocranium resembles that of *Ozarcus* 15,16 and *Dwykaselachus* 3, but the *Ferromirum* gen. nov. neurocranium has suffered post mortem compaction, possibly losing around 25% of its dorsoventral height (Figs 2d, 3c,d). The orbit is large: the maximum span equals the rostrocaudal lengths of otic and occipital regions combined 3. Remains of a slender sclerotic ring are exposed (Fig. 1a-c), but more detailed morphological information is not preserved. Sclerotic rings are known in *Cladoselache* 26, *Denaea* 6, *Falcatus* 7 and *Damocles* 8. A large opening for the optic nerve II perforates the mid-ventral part of the interorbital wall (Fig. 3b), and the slightly expanded anterior margin of the narrow suborbital shelf (Fig. 3b) signals presence of an articulation surface for the palatine ramus of the palatoquadrate.

The postorbital process and arcade (Fig. 3a-d) does not appear to have projected as far laterally and ventrally as those of *Dwykaselachus*, *Ozarcus* and *Akmonistion*. Rather, the process in *Ferromirum*, although likely incomplete, appears shorter, perhaps slightly more robust, proximally, and resembles that of *Gutturensis* 27. Similarly, the *Ferromirum* supraorbital shelf is narrow with a concave lateral margin, a feature also shared by *Ozarcus* as well as *Gutturensis*. Little detail of the otic region is recognizable. The condition of the endolymphatic duct(s) is unclear, but there is no trace of a parietal fossa (Fig. 3a). Traces of the otico-occipital fissure are preserved, revealing that the dorsal portion of the occipital unit is wedged between the otic capsules. The otic wall is too poorly preserved to reveal the presence of a periotic process. The ventral surface of the neurocranium (Fig. 3b) includes the characteristic narrow waist of symmoriids 3,16. The otic region and the glossopharyngeal

Formatted: Highlight

Formatted: Highlight

canals are floored by a hypotic lamina (Fig. 3b). The openings of any canals or grooves for the dorsal aortae, expected to be present in the basicranium, are not visible. However, the occipital plate (Fig. 3d) retains a discernible foramen magnum.

Unlike the braincase, the three-dimensional form of the mandibular arch (Figs. 2a-d, 3e-g, 5e,f, Supplementary Fig. 2, 3) is outstandingly well preserved. The cleaver-shaped 17 palatoquadrate has the high otic process and low palatine process 1,28 common to many early sharks. Otic and palatine portions each account for around half of the total length. A narrow, semi-elliptical surface on the leading edge of the otic process (Fig. 3f) articulates with the postorbital process and arcade of the neurocranium. The external surface of the quadrate and otic portion of the palatoquadrate is strongly concave, forming a broad and deep attachment space for the adductor (quadratomandibularis) muscles. The medial surface is correspondingly convex, but with a gently rounded, oblique ridge marking the ventral boundary of an area that might have been occupied by the spiracular pouch (cf. Brazeau & Ahlberg 29). The otic process rim is prominent throughout all of the posterior and most of the dorsal boundary, thinning-out only at the anterodorsal extremity, just below a ridge and groove that likely supported a mandibular branch of nerve VII (Fig. 3). In lateral aspect (Fig 3f, g), the sigmoid ventral margin of the palatoquadrate is concave downward in the quadrate region and convex downward in the palatine (cf. *Orthacanthus*, 30). In dorsal view the palatine process is mediolaterally broad, forming a substantial portion of the orbit floor (Fig. 2c). The ventral surface bears a shallow dental trough (Fig. 3e, Supplementary Fig. 2c), divided into about nine concavities for generative tooth sets. Anteriorly, a slight additional medial expansion of palatine process bears a ridge and groove articulation with the suborbital process of the neurocranium. The primary articulation with Meckel's cartilage, the quadrate condyle, is at the posterolateral extremity of the palatoquadrate (Fig. 3e, Supplementary Fig. 3). The secondary, medial articulation, the glenoid recess for the mandibular knob or process of Meckel's cartilage, is offset both dorsally and anteriorly (Supplementary Fig. 3). The axis

Comment [HBS11]: I can´t see this in figure 2c

connecting these two surfaces subtends an angle of about 45 degrees relative to the long axis of Meckel's cartilage, and slopes dorsomedially to ventrolaterally at an angle of 45 degrees relative to a horizontal plain connecting left and right quadrates (Fig. 3e).

The lateral, external surface of Meckel`s cartilage (Fig. 3f) is deeply concave for the posterior two thirds of its length, providing a space for adductor muscle insertion. Dorsally, the laterally expanded margin (anterior to the adductor recess) forms a platform for the dentition. The platform is narrow anteriorly but broadens posteriorly: the breadth is considerable (Supplementary Fig. 3), unlike narrower dental platforms present in taxa such as xenacanths 31. Like the palatoquadrate, the dental trough is divided into about nine shallow concavities (Fig. 5c, e,f). A slight posterior rise of the dental platform resembles a coronoid process, but this is simply the posterior limit of the gently concave dental platform, matching the convexity of the corresponding palatine process. The articular region of the lower jaw is situated on the dorsal margin at the posterior extremity (Fig. 5e,f, Supplementary Fig.1). The chief articular facet is a posterolateral concavity, the articular cotylus, which receives the articular process of the palatoquadrate. Anterior to the articular cotylus, the mesial margin is produced into a strong, dorsally directed mandibular knob (Fig. 3g, Supplementary Fig. 3), and this is received by the articular cotylus of the palatoquadrate. As for the upper jaw, the axis (of rotation) of this hinge lies at an angle of about 45° relative to the long axis of the lower jaw. The medial surface of the mandible is generally convex, except of the posteroventral margin, which forms a smooth concavity. In dorsal view, the long axis of Meckel's cartilage is remarkably straight, and shows none of the characteristic curvature evident in the lower jaws of *Tristychius* 21, *Gogoselachus* 32, or xenacanths 30,31. The anterior terminus of the mandible is slender, rounded and shallow, signalling the absence of any substantial, stable symphysial connection: the two halves of the lower jaw meet more or less point-to-point.

The dentition is exposed but damaged (Supplementary Fig. 4). It consists of numerous, small, symmetrical, cladodont teeth, the largest observable bases of which are barely 2 mm across. Each tooth bears a prominent median cusp flanked by diminutive slender lateral cusps; broken sections through some of the larger tooth bases suggest the presence of intermediate cusps.

Like *Ozarcus*, 16 the hyoid arch includes paired hyomandibulae, ceratohyals, and hypohyals (Figs 2a-c, 3e, g). Each hyomandibula is gently curved, anteriorly expanded and laterally compressed. The shape matches that of the Tennessee cladoselachian 33 rather than the more linear outline of the *Ozarcus* hyomandibula, and extends forward to just behind the orbit. However, unlike *Ozarcus*, the hyomandibula meets the ceratohyal directly posterior and medial to the articulation of the mandibular arch.

The ceratohyal is slender and elongate, with a dorsally curved posterior process resembling a walking stick handle (Fig. 3g, Supplementary Fig. 5). This 'handle' fits snugly within a shallow recess between the articular surfaces at the posterior of Meckel's cartilage, from which it ascends to meet the hyomandibula. The ceratohyal-hyomandibula articulation aligns, dorsoventrally, with the apex of the mandibular knob of Meckel's cartilage: i.e., level with the secondary articulation of the mandibular arch. There is a deep fossa in the posterior part of the external surface of the ceratohyal, at the point of maximum dorsal curvature (Supplementary Fig. 5). The ventrolateral margin of the fossa is extended laterally to form a gently convex flange or process that fits neatly within a matching recess in the ventromedial surface of Meckel's cartilage (Figs 1c, 2a). The hypohyal is simple, short, cylindrical and directed anteriorly. There is no trace of a basihyal.

Five gill arches are preserved, including an apparently complete set of ceratobranchials from left and right sides (Fig. 2a-c). Epibranchials, too, include up to five members. All paired cartilages of the gill skeleton are simple rods. There are no remains of hypobranchials

or pharyngobranchials. The basibranchial series is represented by a broad posterior copula 34, somewhat like that of *Gutturensis* 27.

The scapulocoracoid (Fig. 2, Supplementary Fig. 6) resembles those of other symmoriiform chondrichthyans 1,10. The flat scapula blade has a well-developed anterior process at the dorsal apex, but the posterolateral process, although broken, appears rounded. The ventral part of the scapula is mediolaterally broad as it blends into the roof of the articular surface for the pectoral fin. In ventral view, the base of the scapulacoracoid appears triangular and its posterior portion shows a concavity for the articulation with the proximal radials of the pectoral fin. The coracoid region is convex anteriorly and concave posteriorly. A procoracoid has not been detected, although present in other symmoriids 5,10.

The pelvic girdle is known only from a small, simple triangular plate visible in the posterior of a pyrite concretion, which is located near the middle of the body (Fig. 1b, e). In other symmoriiform chondrichthyans such as *Akmonistion*, *Cobelodus*, *Denaea*, and *Symmorium*, the pelvic plate varies from subtriangular to oval 1,10.

A dorsal fin spine is preserved at the level of the pectoral girdle (Fig. 2, Supplementary Fig. 6f). The fin spine resembles those of cladoselachians 1,35 in having a strong, caudally recurved dorsal apex and smooth surface bearing no ridges or tubercles. However, the overall shape and proportions of the spine are considerably narrower and longer than cladoselachian examples in lateral view. Dorsal fin conditions are unknown.

Phylogenetic significance. Results of phylogenetic analysis place the symmoriiform sharks, including *Ferromirum*, as a clade branching from the holocephalan stem, consistent with recent and related results 3,13,14,36 (Fig. 4). Changes are mostly confined to the chondrichthyan stem branching pattern. Notably, *Gladbachus* no longer branches from close to the base of the total group 13, but is instead sister group to *Pucapampella* plus *Gydoselache*, *Doliodus*, and crown chondrichthyans, corroborating results found by Dearden

Comment [HBS12]: I miss something here

et al. 36. The structure of the chondrichthyan crown is reasonably robust, and signals from data partitions are mostly consistent with the overall result. Exclusion tests limiting the characters to neurocranial conditions (characters 1-4; 100-180) recover the branching structure of the chondrichthyan crown obtained from the complete data set (Adams consensus). Similarly, symmoriiforms emerge as stem-holocephalans in searches excluding neurocranial characters. However, in these trees, *Squalus* is the immediate sister of the holocephalan total-group, and putative stem-elasmobranchs (identified in analyses of the complete data set) are excluded from the chondrichthyan crown. Nevertheless, these putative stem-elasmobranchs persist in forming a monophyletic clade that branches from the chondrichthyan stem apex, and this result reoccurs in trees obtained from characters restricted to scales (characters 5-48), teeth (characters 65-86), and fin spines (characters 215-230).

Comment [HBS13]: Name them

Relationships within the symmoriiforms are unstable, and all resolution is lost in a consensus of tree lengths of only one extra step. Even so, the nested position of *Ferromirum* suggests that multiple symmoriiform lineages extended back into the Late Devonian (Fig. 4). Notably, this pattern is found throughout the major divisions of early members of the chondrichthyan crown-clade, and our results imply that much of the diversity of late Palaeozoic Chondrichthyes results from cladogenic events occurring before the Devonian-Carboniferous boundary.

Comment [HBS14]: I fully agree

Anatomical significance. The exceptional 3D-preservation of the jaws and hyoid arch of *Ferromirum* provides new insights into early chondrichthyan cranial structure and function. Initially using computer models with subsequent assembly of a physical model derived from STL files, we found that the jaws and hyoid apparatus could be rescaled to achieve a remarkably precise fit to the undistorted 3D-neurocranium of the much younger, early Permian symmoriiform *Dwykaselachus oosthuizeni* 3 (Fig. 5a-d, Supplementary Fig. 3, 7, 8). This result both tests and corroborates an implicit assumption that the articulations between

the neurocranium and the mandibular plus hyoid arches barely changed throughout the history of the Symmoriiformes. Moreover, this striking morphological conservatism spanning a ~75 million-year age difference between these specimens, echoes genomic evidence of remarkably slow evolutionary rates within the holocephalan lineage relative to all other gnathostome clades 37.

Importantly, in both *Ferromirum* as preserved (Figs 1, 2) and in the *Ferromirum*-*Dwykaselachus* composite model (Fig. 5), the hyomandibula-ceratohyal joint is directly medial to the mandibular arch joint. This is quite unlike the recent interpretation of *Ozarcus* 16, where the joints and arches are spaced one behind the other. Thus, unlike *Ozarcus*, *Ferromirum* includes no space for an enlarged pseudobranch-bearing spiracular pouch or fully respiratory gill pouch. The precise fit between the flanged rear of the ceratohyal and the ventromedial profile of Meckel's cartilage shown in Figure 1c is also present in an undescribed specimen of *Cobelodus aculeatus* (FMNH PF 7351) in the Field Museum (Chicago, IL) collection of sharks from the Mecca fauna of the Pennsylvanian black shales of central North America 5,6. This specimen, like *Ferromirum*, is preserved in a barely disturbed posture with the ventral surface exposed. Comparison with *Chlamydoselachus* suggests that the ceratohyal flange provides the origin of the mandibulo-ceratohyal ligament 38. Taken together, these data strongly imply that the apparent gap between mandibular and hyoid arches in *Ozarcus* and *Cobelodus* 5,16,39 is an artefact of post-mortem lateral compression. Furthermore, rather than having a non-suspensory function, the symmoriiform hyoid arch is specialized and intimately involved in the jaw mechanism, *contra* the tenets of the aphetohyoidean hypothesis and related scenarios of visceral arch evolution 1,40-42.

Comment [HBS15]: Well…you only compare here two taxa. Probably this is true but include more supporting information.

Comment [HBS16]: This sentence is too much speculative. Conservation of a single structure not means slow genetic evolutionary rates. Please USE verbs like suggest or could be indicative

Comment [HBS17]: How is the fit between the flanged rear of the ceratohyal and the ventromedial profile of Meckel's cartilage in this taxa?

Functional morphology of the mandibular and hyoid arches. The physical model (Fig. 5; Supplementary Fig. 8), combining hyoid and mandibular arches of *Ferromirum* and the braincase of *Dwykaselachus*, allowed direct investigation of jaw and hyoid arch motion. The amphistylic 43, or, rather, archaeostylic 44 mode of jaw suspension includes an articulation between the leading edge of the palatoquadrate otic process and the convex rear of the postorbital arcade, and between the palatoquadrate palatine ramus and the orbital articulation. In *Ferromirum* ~~T~~the sigmoid profile of the jaw joint (Fig. 6a) forms a hinge, but, as noted in the description, the rotational axis between primary and secondary articulations is offset relative to the cardinal axes of the cranium (Supplementary Fig. 3). The resulting jaw motion seems counter-intuitive. Meckel's cartilage rolls laterally (eversion: biting surface outwards) along its long axis while the jaw is opening, and medially (inversion: biting surface inwards) as the jaw closes (Fig. 6a,b). There is no broad mandibular symphysis to restrict this rotation during jaw depression and elevation (Fig. 5b). At a gape of 60 degrees, each side of the lower jaw has rolled outwards by approximately 20 degrees relative to its orientation when the jaws are closed (Fig. 6b), and the span of left and right mandibles in ventral view is slightly narrowed: the symphysial angle reduced from 50 (jaws closed) to 45 degrees (jaws open). Thus, as the gape opens, the dentition is everted and a greater proportion of the tooth battery is presented to the surrounding water column and prey. Comparison with analysis of tooth whorl function in *Helicoprion* 45 suggests that older teeth, closer to being shed or displaced onto the lateral surface of the jaw, would have a slighter greater velocity advantage as the jaws close and the dentition is rolled inwards. Such teeth are likely to impale or snag prey before such items are pushed or drawn into the mouth. Lingually located younger and larger teeth might also became functional within the bite because of this rotation. Dental batteries of left and right sides would be rotated medially through a combined angle of 40 degrees (Fig. 6c), scooping material into the gape where the younger, newer, teeth, deeper in the jaws with

Formatted: Font: Not Bold

Comment [HBS18]: Noted that this would imply a optimization of the existing biting strategies rather than the development of new ecomorphological disparity

sharper and larger crowns, could be used to puncture and push prey deeper into the buccal space.

The role of the hyoid arch is not completely understood, but it is clear that it fits closely within the gently recessed rear of the palate and mandible. The archaeostylic palate might be considered self-supporting, but the hyomandibula likely served as an important structural brace limiting dorsoventral movement. Furthermore, manipulation of the model reveals that slight raising of the distal end of the hyomandibula contributes to depression of the ceratohyal and mandible.

To the best of our knowledge, the pattern of jaw motion described here is unknown in living fishes *sensu lato*, although a hemimandibular roll is considered crucial to oral processing in early mammals 19. Among Palaeozoic sharks, *Ferromirum* is quite unlike the specialized saw-jawed stem-holocephalan *Helicoprion* 45 and the suction feeding stem-elasmobranch *Tristychius* 21. However, key aspects of the feeding apparatus in *Ferromirum* are widespread among early chondrichthyans 1,2,5-8: a slender ceratohyal, an elongate hyomandibula, a jaw joint far behind the orbit (at the extremity of a cleaver-shaped palate), and no trace of labial cartilages. For these reasons, we suggest that *Ferromirum* likely provides a glimpse of more general functional conditions in early ~~sharks~~holocephalans. Notably, in the Maïder Basin, *Ferromirum* is one of several chondrichthyans occurring within sediments rich in thylacocephalan crustaceans 22,46 (Fig. 7). It appears likely that ram feeding, employing a large gape lined with generative sets of cladodont teeth, with those of the lower jaw rotating symmetrically inwards as the mouth snaps shut, would provide an effective means of capturing and retaining such seemingly abundant invertebrate prey.

Preservation quality has limited previous studies of early chondrichthyan jaw mechanics 44 but insights from exceptionally preserved 3D-material might be used to identify morphological correlates of similar function among richer collections of flattened specimens. 2D-jaw shape measures of biomechanical traits have already been used to estimate functional

Comment [HBS19]: - Rate of Tooth replacement rates in early chondrichthyans has been prove to be very slow (Botella et al. 2009
-Moreover tooth retention is known to occur (at least) in cladoselachians -ME Williams - 2001
In consequence older (more labial) teeth could be very worn (in fact they are !!).
The mechanism you suggest here could allow to newer and unwear teeth become functional

Formatted: Font: Italic

Comment [HBS20]: Mostly **symmoriiform, but what about not holocephalans?**

disparity and implied ecological variety across early gnathostomes 47. Thus, from the present work, the location of a mesial process (mandibular knob) relative to the primary articulation surface might be used similarly as a predictor of 3D jaw motion; especially hemimandibular long-axis rotation if also associated with a slender mandibular symphysis. In this regard, the jaws of symmoriiforms such as *Denaea* and *Symmorium* 6 closely match those of *Ferromirum*. Moreover, the mandibular mesial process is a well-established chondrichthyan synapomorphy 47 occurring deep within the stem lineage. It would be interesting to learn how the distribution of this process, and specialized jaw hinge, correlates with the evolution of the classic, tooth-whorl dominated, shark dentition.

Methods

Studied material. The type and only specimen (PIMUZ A/I 4806) of *Ferromirum oukherbouchi* gen. et sp. nov. is housed at the Palaeontological Institute and Museum of the University of Zurich, Switzerland. The specimen was prepared out of a ferruginous reddish nodule (rich in haematite) from the Famennian (Late Devonian) of Madene El Mrakib in the Maïder region of the southeastern Anti-Atlas (Morocco).

Phylogenetic analysis. We performed a heuristic search in PAUP* 4.167 49 using a parsimony ratchet 50 with an initial 10,000 random sequence additions. The character matrix consists of 64 taxa, 56 ingroup and eight outgroup taxa, coded for 230 morphological characters. The analysis recovered 90 equally most parsimonious trees (MPTs) of 535 steps (consistency index 0.46; retention index 0.78; RC 0.36). We assessed nodal support through bootstrapping and Bremer Decay indices. Please see Supplementary Note 1 for further details.

Comment [HBS21]: (Adams consensus) for consensus tree

X-ray micro-tomography. High resolution data were obtained using an industrial computed tomography scanner (Nikon XT H 225 ST) at the University of Zurich, Switzerland. Data

acquisition and image reconstruction parameters are: 224 kV, 474µA; filter: 4 mm of copper; isotropic voxel dimensions of 0.091 mm; 16-bit TIFF images were acquired; XX 8-bit TIFF images were used for reconstruction. Images were analysed and 3D models reconstructed using Mimics v.17 (http://www.biomedical.materialise.com/mimics; Materialise, Leuven, Belgium). The 3D-object was edited (smoothing, colours and lightning) in MeshLab v. 2016 (http://www.meshlab.net; 41) and blender v2.79b (https://www.blender.org; Amsterdam, Netherlands).

Virtual and physical models. 3D anatomical reconstructions of *Ferromirum gen. nov.* palatoquadrate, Meckel`s cartilage, hyomandibula and ceratohyal, and *Dwykaselachus* neurocranium used Mimics v. 18 (biomedical.materialise.com/mimics; Materialise, Leuven, Belgium) for three-dimensional modelling, STL polygon creation and kinematic simulations. Further editing of the STLs (colour, texture, lighting), kinematics and mirroring for the final restorations (virtual images, virtual manipulations, and 3D printouts of STL files) used 3D Studio Max (Autodesk.com/products/3ds-max; Autodesk, San Rafael, USA).

Nomenclatural acts. This published work has been registered in ZooBank, the proposed online registration system for the International Code of Zoological Nomenclature (ICZN). The ZooBank LSIDs (Life Science Identifiers) can be resolved and associated information viewed through any standard web browser by appending LSID to the prefix "http://zoobank.org/." The LSIDs for this publication are: zoobank.org.pub:XXXXXXXX

Reporting summary. Further information supporting the results and discussion of this study is available in the Nature Research Reporting Summary linked to this article.

Data availability

The authors declare that all data supporting the findings of this study are available at Dryad XX.

References

1. Zangerl, R. *Handbook of Paleoichthyology volume 3A: Chondrichthyes I, Paleozoic Elasmobranchii.* (ed. Schultze, H.P) (Gustave Fischer, 1981).
2. Janvier, P. *Early Vertebrates* (Clarendon, 1996).
3. Coates, M. I., Gess, R. W., Finarelli, J. A, Criswell, K. E, & Tietjen, K. A symmoriiform chondrichthyan braincase and the origin of chimaeroid fishes. *Nature* **541**, 209-211 (2017).
4. Guinot, G., Adnet, S., Cavin, L. & Cappetta, H. Cretaceous stem chondrichthyans survived the end-Permian mass extinction. *Nat. Comm.* **4**, 2669 (2013).
5. Zangerl, R. & Case, G. R. *Cobelodus aculeatus* (Cope), an anacanthous shark from Pennsylvanian Black Shales of North America. *Palaeontographica A* **154**, 107–57 (1976).
6. Williams, M. E. The ‘‘cladodont level’’ sharks of the Pennsylvanian Black Shales of central North America. *Palaeontographica A* **190**, 83–158 (1985).
7. Lund, R. The morphology of *Falcatus falcatus* (St. John and Worthen), a Mississippian stethacanthid chndrichthyan from the Bear Gulch Limestone of Montana. *J. Vert. Paleontol.* **5**, 1-19 (1985).
8. Lund, R. On *Damocles serratus* nov. gen. et sp., (Elasmobranchii: Cladodontida) from the Upper Mississippian Bear Gulch Limestone of Montana. *J. Vert. Paleontol.* **6**, 12-19 (1986).
9. Coates, M. I., Sansom, I. J., Sequeira, S. E. K. & Smith, M. M. Spines, teeth and histology: novel structures and tissues in ancient sharks. *Nature* **396**, 729-730 (1998).
10. Coates, M. I. & Sequeira, S. E. K. A new stethacanthid chondrichthyan from the Lower Carboniferous of Bearsden, Scotland. *J. Vertebr. Paleontol.* **21**, 754–766 (2001).

11. Pradel, A., Tafforeau, P., Maisey, J. G. & Janvier P. A new Paleozoic Symmoriiformes (Chondrichthyes) from the Late Carboniferous of Kansas (USA) and Cladistic Analysis of Early Chondrichthyans. *PLoS ONE* **6**: e24938 (2011).
12. Maisey, J. G., Miller, R., Pradel, A., Denton, J. S. S., Bronson A. & Janvier, P. Pectoral morphology in *Doliodus*: bridging the 'acanthodian'-chondrichthyan divide. *Am. Mus. Novit*. **3875**, 1-15 (2017).
13. Coates, M. I., Finarelli, J. A., Sansom, I. J., Andreev, P. S., Criswell, K. E, Tietjen, K., Rivers, M. L. & La Riviere, P. J. An early chondrichthyan and the evolutionary assembly of a shark body plan. *Proc. R. Soc. Ser. B.* **285**: 20172418 (2018).
14. Frey, L., Coates, M. I., Ginter, M., Hairapetian, V. Rücklin, M., Jerian, I. & Klug, C. The early elasmobranch *Phoebodus*: phylogenetic relationships, ecomorphology, and a new time-scale for shark evolution. *Proc. R. Soc. Ser. B.* **286**: 20191336 (2019).
15. Maisey, J. G. The braincase in Paleozoic symmoriiform and cladoselachian sharks. *Bull. Am. Mus. Nat. Hist*. **307**, 1-122 (2007).
16. Pradel, A., Maisey, J. G., Tafforeau, P., Mapes, R. H. Mapes & Mallatt, J. A Palaeozoic shark with osteichthyan-like branchial arches. *Nature* **509**, 608-611 (2014).
17. Schaeffer B. In *Problèmes actuels de paléontologie, évolution des vertébrés*. (ed. Lehman, J. P.) 101-109 (Colloque Internationaux CNRS, 1975).
18. Motta, P. J. & Huber, D. R. In *Biology of sharks and their relatives*, (eds. Carrier, J. C., Musick, J. A., Heithaus, M. R.) 153-209 (CRC Press, 2012).
19. Bhullar, B-A. S., Manafzadeh, A. R., Miyamae, J. A., Hoffman, E. A., Brainerd, E. L., Musinsky, C. & Crompton, A. W. Rolling of the jaw is essential for mammalian chewing and tribosphenic molar function. *Nature* **566**, 528-532. (2019)
20. Brazeau, M. D. & Friedman, M. The origin and early phylogenetic history of jawed vertebrates. *Nature* **520**, 490-497 (2015).

21. Coates, M. I., Tietjen, K., Olsen, A. M. & Finarelli, J. A. High-performance suction feeding in an early elasmobranch. *Sci. Adv*. 2019; **5**: eaax2742 (2019).

22. Frey, L., Rücklin, M., Korn, D. & Klug, C. Late Devonian and Early Carboniferous alpha diversity, ecospace occupation, vertebrate assemblages and bio-events of southeastern Morocco. *Palaeogeogr. Palaeoclimatol. Palaeoecol.* **496**, 1-17 (2018).

23. Frey, L., Pohle, A., Rücklin, M. & Klug, C. Fossil-Lagerstätten and preservation of vertebrates and invertebrates from the Devonian of Morocco (eastern Anti-Atlas). *Lethaia*, 25 pp, https://doi.org/10.1111/let.12354 (2019).

24. Huxley, T. *A manual of the anatomy of vertebrated animals*. (D-Appleton and Co., 1880).

25. Bonaparte C. L. J. L. *Iconografia della fauna italica per le quatro classi degli animali vertebrati. Tomo III: Pesci*. (Salviucci, 1838).

26. Dean, B. Studies on fossil fishes (sharks, chimaeroids and arthrodires. *Memoirs of the AMNH* **9(5)**, 209-287 (1909C).

27. Sequeira, S. E. K. & Coates, M. I. Reassessment of *'Cladodus' neilsoni* Traquair: a primitive shark from the Lower Carboniferous of East Kilbride, Scotland. *Palaeontology* **43**, 153-172 (2000).

28. Schaeffer B. The xenacanth shark neurocranium, with comments on elasmobranch monophyly. *Bull. Am. Mus. Nat. Hist.* **169**, 1–66 (1981).

29. Brazeau, M. D. & Ahlberg, P. E. Tetrapod-like middle ear architecture in a Devonian fish. *Nature* **439**, 318-321 (2006).

30. Hotton, N. Jaws and teeth of American xenacanth sharks. *J. Paleontol.* **26**, 489-500 (1952).

31. Hampe, O. Revision of the Xenacanthida (Chondrichthyes: Elasmobranchii) from the Carboniferous of the British Isles. *Earth Environ. Sci. Trans. R. Soc. Edinb.* **93**, 191-237 (2002).

32. Long, J. A., Burrow, C. J., Ginter, M., Maisey, J. G., Trinajstic, K. M., Coates, M. I. et al. First shark from the Late Devonian (Frasnian) Gogo Formation, Western Australia sheds new light on the development of tessellated calcified cartilage. *PLoS ONE* **10**, e0126066 (2015).

33. Maisey, J. G. Visceral skeleton and musculature of a Late Devonian shark. J. *Vertebr. Paleontol.* **9**, 174-190 (1989).

34. Nelson, G. J. Gill arches and the phylogeny of fishes, with notes on the classification of vertebrates. *Bull. Am. Mus. Nat. Hist.* **141**, 475–552 (1969).

35. Harris, J. E. The dorsal fin spine of *Cladoselache*. *Sci. publ. Clevel. Mus. Nat. Hist.* **8**, 1-6 (1938).

36. Dearden, R. P., Stockey, C. & Brazeau, M. D. The pharynx of the stem-chondrichthyan *Ptomacanthus* and the early evolution of the gnathostome gill skeleton. *Nat. Commun*. **10**, 2050. https://doi.org/10.1038/s41467-019-10032-3 (2019).

37. Venkatesh, B. *et al.* Elephant shark genome provides unique insights into gnathostome evolution. *Nature* **505**, 174-179 (2014).

38. Allis, E. P. The cranial anatomy of *Chlamydoselachus anguineus*. *Acta Zool*. **4**, 123-221(1923).

39. Zangerl, R. & Williams, M. E. New evidence of the nature of the jaw suspension in Palaeozoic anacanthous sharks. *Palaeontology* **18**, 333-341 (1975).

40. Gegenbaur, C. *Untersuchungen zur vergleichenden Anatomie der Wirbelthiere. III. Das Kopfskelet der Selachier, ein Beitrag zur Erkenntniss der Genese des Kopfskeletes der Wirbelthiere* (Engelmann, Leipzig, 1872).

41. De Beer 1937 G. R. DeBeer, *The Development of the Vertebrate Skull* (Univ. Chicago, 1937).

42. Mallat, J. Ventilation and the origin of jawed vertebrates: a new mouth. *Zool. J. Linn. Soc.* **117**, 329-404 (1996).

43. Huxley, T. H. Contributions to morphology. Ichthyopsida. No 1. On *Ceratodus forsteri*, with observations on the classification of fishes. *Proc. Zool. Soc. London* **1876**, 24-59 (1876).

44. Maisey, J. G. The postorbital palatoquadrate articulation in elasmobranchs. *J. Morphol.* **269**, 1022-1040 (2008).

45. Ramsay, J. B., Wilga, C. D., Tapanila, L., Pruitt, J., Pradel, A., Schlader, R. & Didier, D. A. Eating with a saw for a jaw: functional morphology of the jaws and tooth-whorl in *Helicoprion davisii*. *J. Morphol.* **276**, 47-64 (2015).

46. Jobbins, M., Haug, C. & Klug, C. First African thylacocephalans from the Famennian of Morocco and their role in Late Devonian food webs. *Sci. Rep.* **10**, 5129 (2020).

47. Anderson, P. S. L., Friedman, M., Brazeau, M. D. & Rayfield, E. J. Initial radiation of jaws demonstrated stability despite faunal and environmental change. *Nature* **476**, 206-209 (2011).

48. Davis, J.A. Finarelli, M.I. Coates, *Acanthodes* and shark-like conditions in the last common ancestor of modern gnathostomes. *Nature* **486**, 247-250 (2012).

49. Swofford, D. L. *PAUP*: Phylogenetic Analysis Using Parsimony* (*and other methods). (Sinauer Associates, 2003).

50. Quicke, D. L. J., Taylor, J. & Purvis, A. Changing the landscape: a new strategy for estimating large phylogenies. *Syst. Biol.* **50**, 60-66.

Acknowledgements

We are deeply indebted to Saïd Oukherbouch (Tafraoute, Morocco) who discovered the holotype and who supported us during field work. The holotype was prepared by the team of the Tahiri Museum of Fossils and Minerals (Erfoud, Morocco). Anita Schweizer (Zurich) and Alexandra Wegemann (University of Zurich) kindly acquired CT-scans for us. Many thanks to Thodoris Argyriou (University of Zurich) who helped segmenting the image stack. Michal Ginter (Warsaw) helped with the interpretation of the teeth.

Author contributions: L.F.: segmentation of computer tomographs, preparing figures, drafting manuscript. C.K., M.C., L.F.: creating the project. C.K.: photography, illustrations, formatting. K.T.: segmentation of computer tomographs, preparing figures, 3D-printing. M.C.: phylogenetic analysis, assembly and interpretation of 3D-models, photography, preparing figures. All authors contributed to the text.

Funding: The project (number 200020_184894) was financially supported by the Swiss National Science Foundation. M.R. was supported by NOW Vidi grant no. 864.14.009.

Competing interests: The authors declare no competing interests.

Additional information

Supplementary Information is available for this paper at XX.

Correspondence and requests should be addressed to CK and MC.

Figures

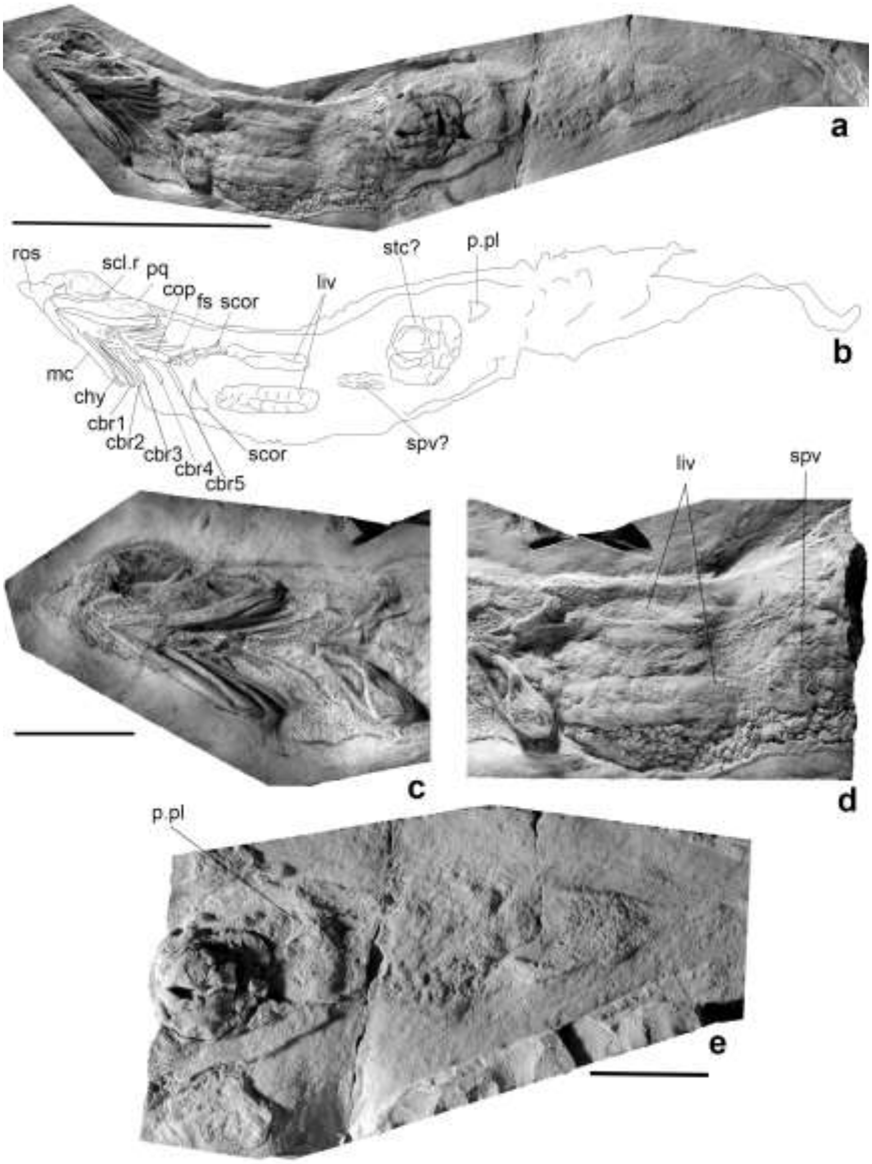

Fig. 1 *Ferromirum oukherbouchi* gen. et sp. nov., PIMUZ A/I 4806, early/ middle Famennian, Madene el Mrakib. **a** Photo and **b** line drawing of the specimen. **c** Head region including parts of the rostrum, sclerotic ring, mandibular arch, hyoid arch, branchial skeleton and shoulder girdle in ventral view. **d** Soft tissue remains including liver and spiral valves. **e** Pelvic and caudal region. Scale bars, 100 mm (**a**, **b**), and 30 mm (**c**-**e**). chy, ceratohyal; cop, copula; cbr, ceratobranchials; fs, fin spine; liv, liver; mc, Meckel`s cartilage; p.pl, pelvic plate; pq, palatoquadrate; ros, rostrum; scl.r, sclerotic ring; scor, scapulocoracoid; stc?, stomach content; spv, spiral valves.

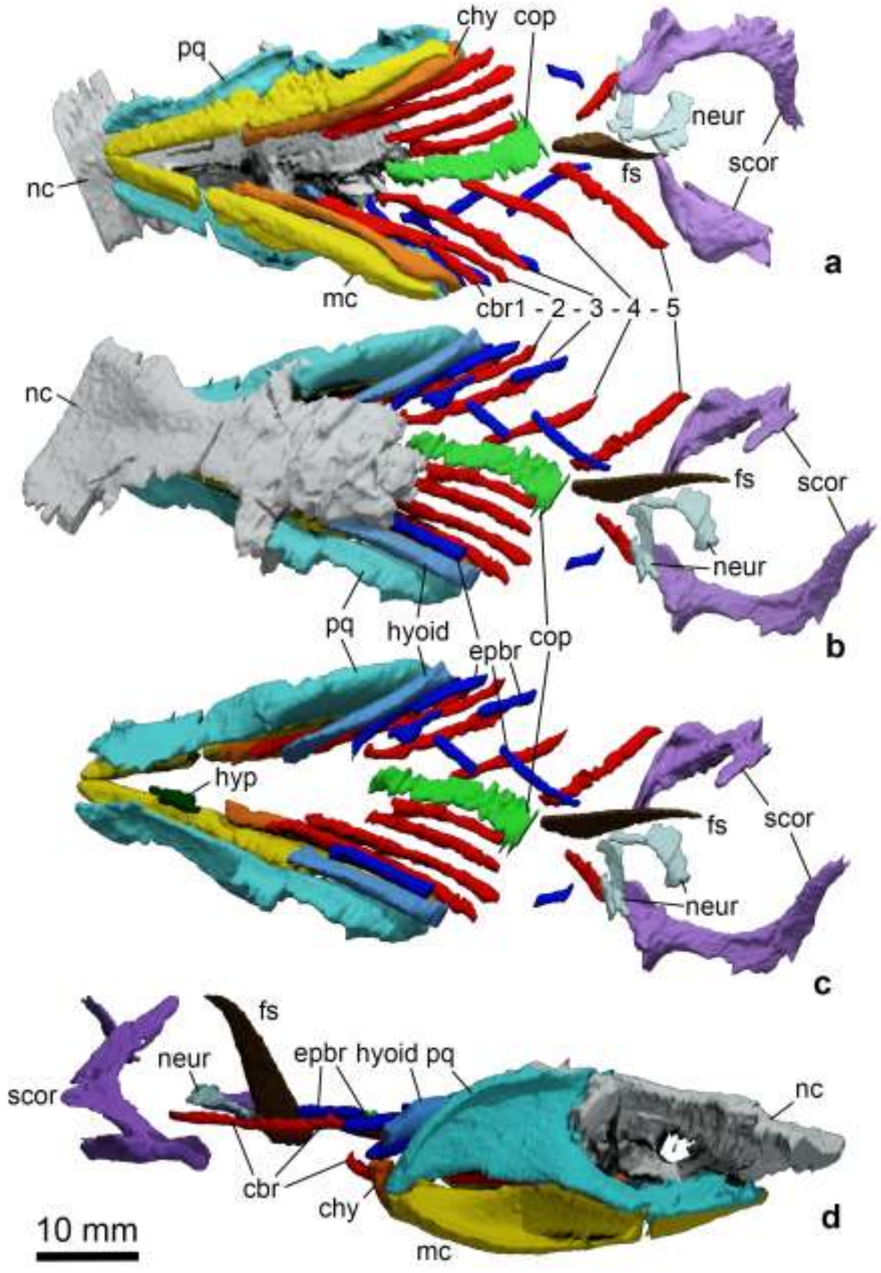

Fig. 2. *Ferromirum oukherbouchi* gen. et sp. nov. PIMUZ A/I 4806, virtual rendering of specimen based on CT-data showing the neurocranium, visceral arches, pectoral girdle and dorsal fin spine. **a** Ventral and **b** dorsal view with and **c** without braincase. **d** lateral view. Colour coding: grey, neurocranium (nc); turquoise, palatoquadrate (pq); yellow, Meckel's cartilage (mc); dark green, hypohyal (hyp); light blue, hyoid (hyoid); orange, ceratohyal (chy); blue, epibranchials (epbr); red, ceratobranchials (cpbr); green, copula (cop); brown, fin spine (fs); purple, pectoral girdle (scor); light turquoise, ? neural arches (neur).

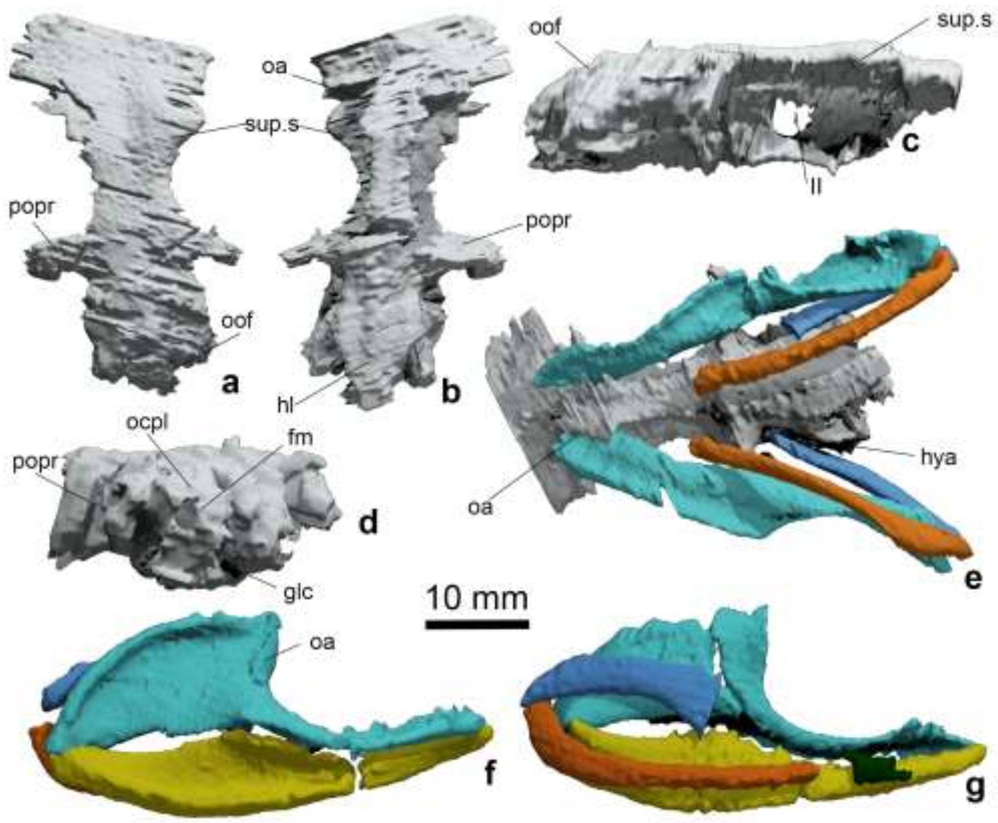

Fig. 3. *Ferromirum oukherbouchi* gen. et sp. nov., PIMUZ A/I 4806. Neurocranium in **a** dorsal, **b** ventral, **c** lateral, and **d** posterior, occipital views. **e** Articulation between braincase, palatoquadrate and hyoid arch in ventral view. **f**, **g** Arrangement of mandibular and hyoid arches in lateral and medial views respectively. Colour coding: grey, braincase; turquoise, palatoquadrate; yellow, Meckel's cartilage; dark green, hypohyal; light blue, hyoid; orange, ceratohyal. Note that the rostral roof includes an excess of poorly resolved cartilage or matrix left in place in the computer renderings. fm, foramen magnum; hl, hypotic lamina; hya, hyomandibular articulation; glc, glossopharyngeal canal; oa, orbital articulation; ocpl, occipital plate; oof, otico-occipital fissure; popr, postorbital process; sup.s, supraorbital shelf; **II**, optic nerve.

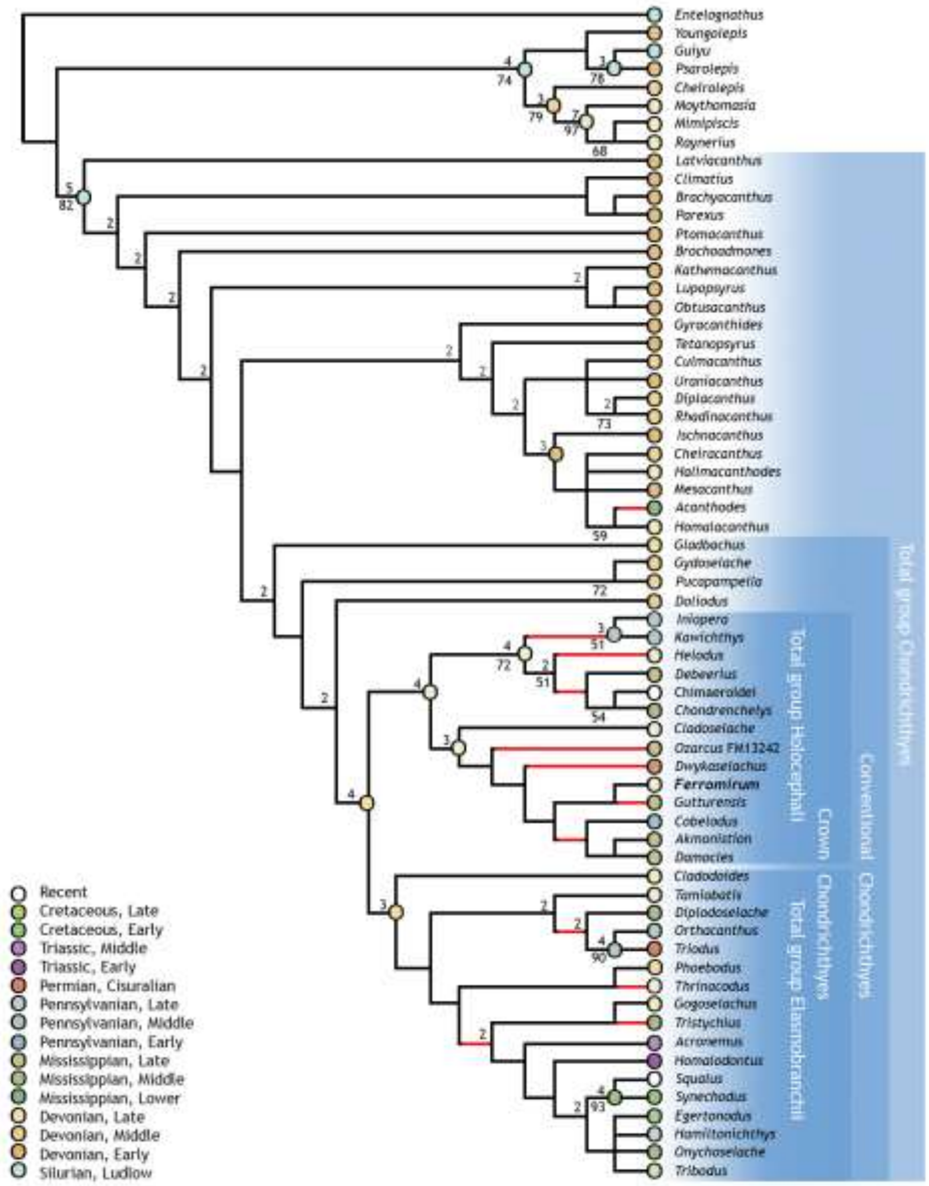

Fig. 4. Strict consensus of 90 trees showing phylogenetic affiliation of *Ferromirum* gen. nov. among early chondrichthyans. Bremer decay values above nodes; bootstrap support scores below. Circle colours (nodes and termini) denote earliest occurrence of taxon; red branches denote clade crossing Devonian-Carboniferous boundary.

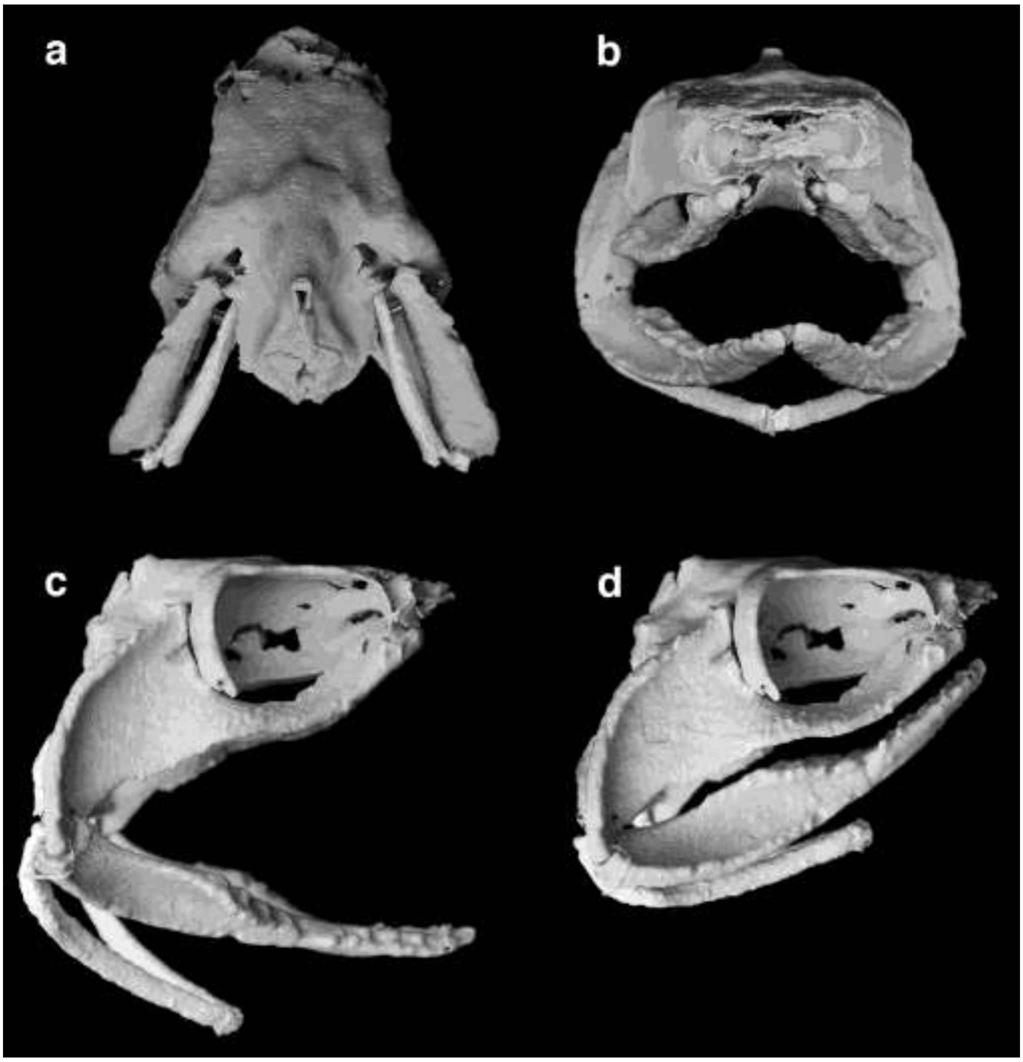

Fig. 5. Composite model of *Ferromirum* gen. et sp. nov. jaws and hyoid arch and *Dwykaselachus* neurocranium, in **a** dorsal, **b** anterior, **c** lateral with mandible and ceratohyal depressed, and **d** with mandible and ceratohyal raised.

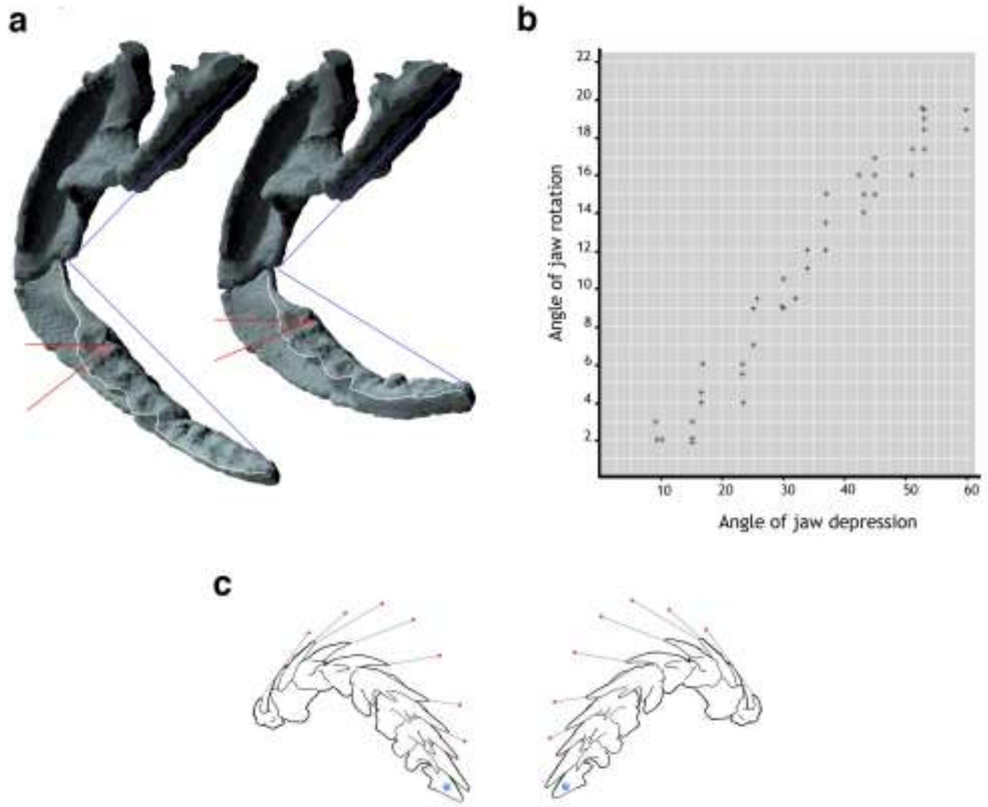

Fig. 6. **a** *Ferromirum* gen. et sp. nov. computer rendering of jaws in different degrees of Meckel's cartilage depression, in anterolateral view. **b** Graphed angle of Meckel's cartilage rotation relative to angle of depression, measurements taken from model in Fig. 5. **c** Line drawing of opposing lower jaw tooth sets, adapted from *Ozarcus* (16). Lines and red dots and mark displacement of crowns when rotated through 22 degrees (graphic convention adapted from ref. 45).

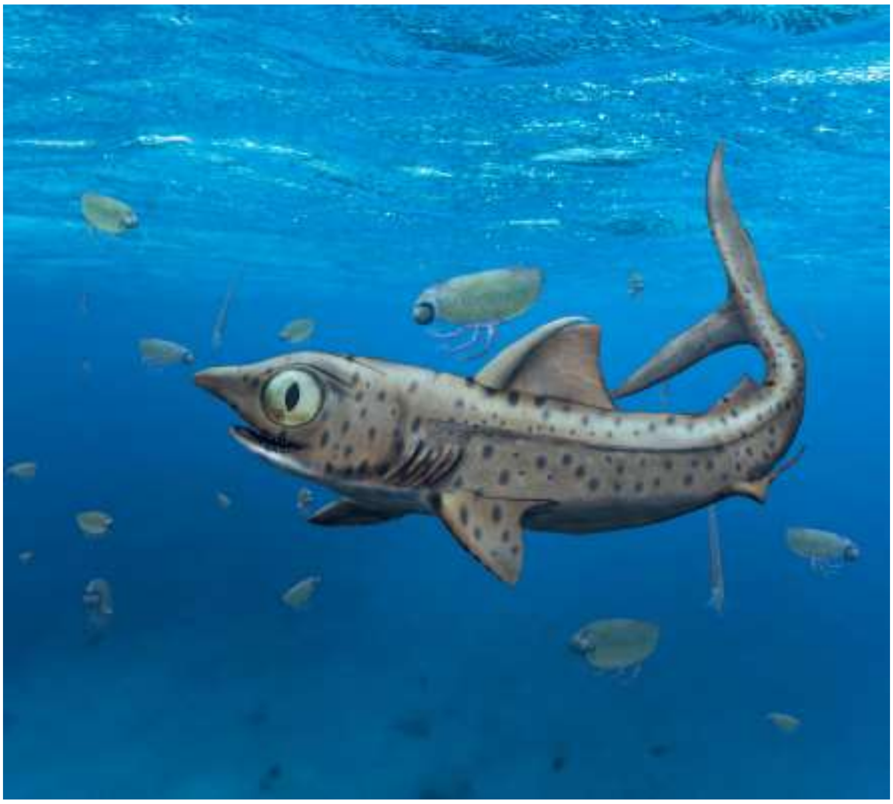

Fig. 7. Reconstruction of *Ferromirum oukherbouchi* gen. et sp. nov. and associated invertebrates (orthocerid cephalopods and thylacocephalans: *Concavicaris submarinus*) from the Famennian of the Maider region (Morocco).

A new symmoriiform from the Late Devonian of Morocco: novel jaw function in ancient sharks

by

L. Frey, M. I. Coates, K. Tietjen, M. Rücklin and C. Klug

Reviewers' comments:

Reviewer #1 (Remarks to the Author):

This manuscript describes a very well-preserved specimen of a Late Devonian symmoriiform chondrichthyan, Ferromerum. Importantly the jaws and hyoid arch are particularly well-preserved, allowing for detailed description and a reconstruction of the physical relationships between these units. Surprisingly, the authors suggest a unique lateral component of jaw rotation during feeding. I have only minor comments on the attached, in particular, I'm curious about the calcified cartilage of the skeleton- can this be described further, and would this relate to jaw/ feeding function?

The cartilage itself is only moderately well-preserved. We feel that this would deserve its own publication and goes beyond the scope of our paper.

Remarks from reviewer attachment 1:

1. Can you label these? I'm not sure what you're referring to in the SI Figure 2i

Arrows were inserted.

2. "The trunk region includes two elongate lobes of the liver (Fig. 1d), extending for perhaps 50% of the visceral cavity length." How does this compare to recent chondrichthyans?

We added some remarks.

3. Also in Figure 1d- there's no question mark in this figure, but there is in Fig. 1b?

Since the spiral valve shows its spiral structure, we are confident about this interpretation and removed the question mark.

4. Can you describe this calcified cartilage in more detail? And in particular, how this might be related to jaw function and feeding- a stiffer jaw, versus less stiff?

The preservation of the cartilage is insufficient to provide information about its structure or strength.

5. Why is a comparison with a crown-group elasmobranch relevant?

Chlamydoselachus *is used for comparison because, unlike other extant chondrichthyans, but like our fossil subject* Ferromirum*, it has a long gape (with long lower and upper jaws). Furthermore,* Chlamydoslachus *musculoskeletal anatomy has been described in detail, thus allowing similarly detailed comparison with features of the ceratohyal in* Ferromirum - *in particular the possible location of a mandibulo-ceratohyal ligament.*

6. Is this also related to the large jaw platform described above?

Could be, but a more detailed (and thus speculative) model would be required. We added the adjective 'broad' to tooth battery to include this thought.

7. Do you see any evidence of tooth root fusion that would prevent tooth shedding?

There is no evidence for fusion, thus no mention.

8. Can you expand upon this? It's not clear what you mean. Maybe discuss this velocity advantage more fully in *Helicoprion*?

We added an explanatory phrase. However, we do not feel that explaining Helicoprion feeding *biomechanics is within the scope of this paper. The reference (45) provides a detailed and clear biomechanical description of the kinematics.*

9. Does it also contribute to the lateral movement of the jaws as the ceratohyal is depressed? Or a brace for this movement?

No, there appears to be little scope for lateral movement of the jaws, not least because of the dorsoventrally extensive articulation between the arcade and the anterior surface of the postorbital process of the palatoquadrate. Most freedom of movement, when the hyoid arch is excluded, is in the d-v dimension, pivoting the PQ from the ethmoid articulation and sliding it up and down the postorbital arcade. This movement is what the hyoid arch attachment prevents. If anything, when the ceratohyal is depressed, the dorsally offset proximal articulation tends to push the distal end of the hyomandibula dorsally (and, vice versa, as noted in the text, "manipulation of the model reveals that slight raising of the distal end of the hyomandibula contributes to depression of the ceratohyal and mandible".

Reviewer #2 (Remarks to the Author):

Overall: A most interesting paper that is a valuable contribution to our knowledge of early shark evolution and anatomy and the understanding of generalized gnathostome feeding mechanisms. The descriptions are based on the well-preserved specimen plus extensive segmentation of CT scans which enable 3D restoration of most of the internal structures.
The fact that soft tissues are well preserve d in the specimen (liver, gut) indicates it is an exceptional specimens so there is no hesitation accepting the interpretations of CT data as reliable. The interpretations about the feeding mechanism, utilizing a unique (for sharks) 'hemimandibular long axis rotation of the jaws' makes this paper particularly significant in showing the high level of disparity and plasticity in feeding biomechanics for Devonians sharks, a group generally thought to be basal in many respects.

SPECIFIC POINTS /CORRECTIONS

1. Line 15 subject is singular (the fossil record…) so should be " is poor" not "are'
Done

2. Lines 73-80. Diagnosis is based on one sample only. So relative terms like large or medium might not mean much unless actual rations included -eg large orbits relative to what -skull or head length? Give a ratio so we can compare with other sharks to see if it really is proportionately larger than say *Cobelodus*?.
Done, some ratios included now.

3. As the majority of fossil sharks are known and defined only by their teeth I would like to see a few statements defining the teeth unique features added to the diagnosis. This would be very useful for future workers who might find isolated teeth of this species (eg cut/paste from lines 181-183).
Dental characters were added to the diagnosis.

4. Line 140. Reference to Brazeau & Ahlberg (2006) is to *Panderichthys*, a highly advanced sarcopterygian with a spiracular opening on top of the head, not laterally. I don't think this is relevant here to sharks, perhaps better to use a reference to spiracular pouches in chondrichthyans?
With respect, the reference is chosen precisely because this source is the first, to the best of our knowledge, to identify the oblique ridge on the medial surface of the PQ, and to associate this with a large spiracular pouch. While their paper is about tetrapods, the figures illustrate this phenomenon in Eusthenopteron *as a means of explaining the general condition (for gnathostomes as much as osteichthyans) preceding tetrapod middle ear evolution. Credit where it's due - and in this instance, to Brazeau & Ahlberg: illustration and explanation clear. A brief noted has been added, in parenthesis.*

5. Line 163 Fig 5 e,f there is no e, f in Fig 5, do you mean Fig 3 as well? Please correct.
Done

6. Line 203-04: in diagnosis (lines 78-79) it is stated " no basihyal " yet this is not discussed in the

description of the gill arches, should be included. Perhaps it is softer cartilage or not preserved for some reason?
Done. A brief note and comparison with Ozarcus *has been added.*

7. Phylogeny: noted that the symmoriform clade is weakly supported and discussed as so (line 241).

The figures are excellent and go beyond what is normally presented in such papers by offering complete models of the 3-D printed scan data which enable greater accuracy in interpretation of functional biomechanics of the jaws and feeding mechanism,.

8. Fig. 4 ‘*Iniopera*’ incorrect-should be –“Inioptera”

Here, the OTU is Iniopera, *the sybyrhynchid iniopterygian.*

Reviewer #3 (Hector Botella; Remarks to the Author):

This paper is a remarkable piece of work comprising two main points.
First description of a new early shark (unusually well 3-d preserved), Ferromirum oukherbouchi gen. and sp. nov, from the Late Devonian of the Anti-Atlas. Authors analyzed its systematic affinity, as well as the anatomical and phylogenetic implications provide by the new discovery.
The second main point investigates the morphology and biomechanics of the very well 3D-preserved jaws and hyoid arch of *Ferromirum* in the context of early chondrichthyans. Authors reliably demonstrate the existence of a novel specialized jaw articulation revealing hemimandibular long-axis rotation. This pattern of jaw motion is unknown in current fishes and suggest an increased morphofunctional diversity in early gnathostomates

The paper is clearly written and well structured. Figures are well performed and support the text. I consider the number of figures appropriate. This work would be of high-interest to a broad audience, (not only Paleobiology but also biomechanics, zoology, anatomy, evolution, ecology ….) and contributes to a number of significant debates about early gnathostome evolution.

All of the methods used are correct for the studies in hand. Specimen collection and repository are well documented and appropriate. Phylogenetic analysis are properly conducted. Data set is updated and extensive and it is available as Sup. Information. I agree with the topology of tree in figure 4 (even the nesting of *Gladbachus*). Overall, I would recommend this paper for publication in Communications Biology. My only minor concerns refer to two aspects of the manuscript.

1- Some parts of the description of the new specimen are not merely description but include comparisons and inferences from the authors that could be better placed in a Remarks section (or easier, moved to next section Anatomical significance).

2- Authors objectively demonstrate morphological and consequent biomechanics diversity in jaw articulation of early chondrichtyans. However, they also claim for an associate an increased ecological disparity. I can accept this point, but it seems not clearly addressed in the paper (see the annotated document)

Remarks from reviewer attachment 1:

1. Rays and Chimaeras are not early chondrichthyans! And... according to the title …What is *Ferromirum* a shark or an extinct relative… I recommend rethink this unprecise first sentence.

We slightly changed the title and the first phrase to be more precise.

2. Gen. et sp. nov.: Here and through all the ms.

It is present except in the abstract because we are already at the limit of the word count for the abstract, thus it was omitted. We suggest the editors decide on this.

3. "This suggests an increased functional and ecomorphological disparity among chondrichthyan jaws preceding and surviving the end-Devonian extinctions." Insert jaws.

We wanted to make a more general statement here and explicitly did not limit this to jaws but to the whole animal. Already in the Devonian, there is quite some disparity in dentition, jaw function and body form, which does support such a broader statement.

4. All this paragraph seems part of the abstract of the work. I do not think" introduction" is the appropriate section to include it.

We do understand the reviewer's point, but we feel it is necessary to elaborate on this topic here since space in the abstract is very limited and it is an important aspect of the paper. However, we have shortened the first phrase of this paragraph in order to reduce the abstract-impression.

5. Picture of ecomorphological disparity is also emerging early in jawless fishes:

Ferrón et al 2020: Categorical versus geometric morphometric approaches to characterizing the evolution of morphological disparity in Osteostraci (Vertebrata, stem Gnathostomata).

Ferrón et al 2018: Patterns of ecological diversification in thelodonts

These are interesting papers and indeed also in jawless forms, an ecological diversification is present, but we discussed jawed vertebrate ecomorphological disparity. We do not see the relevance here and decide not to include the citations.

6. "As in these genera, there is no evidence of skeletal support for the rostral apex, which likely housed arrays of electro- and mechanoreceptive organs". Discussion or comparison, not description. Also several sentences below - I mark some in yellow but please check.

This is handled quite variably. We feel the text is more concise like this. Up to the editors.

7. "putative stem-elasmobranchs (identified in analyses of the complete data set) are excluded from the chondrichthyan crown": Name them.

In order to avoid writing out a lengthy list of genera names the text has been expanded, with an additional reminder to view Figure 4. Now page 10.

8. Well…you only compare here two taxa. Probably this is true but include more supporting information.

We added a reference.

9. This sentence is too much speculative. Conservation of a single structure not means slow genetic evolutionary rates. Please USE verbs like suggest or could be indicative.

We use only the verb 'echo' because this is no more than a tantalizing repetition -but note the recent report of the successful hybrid of sturgeon and paddlefish - taxa that are also morphologically conservative across ~100+ million years. Morph conservatism is not an entirely unreasonable proxy for genetic conservatism.

10. How is the fit between the flanged rear of the ceratohyal and the ventromedial profile of Meckel's cartilage in this taxon?

Done.

11. The mechanism you suggest here could allow to newer and unwear teeth become functional.

Such a remark was inserted.

12. Mostly symmoriiform, but what about non-holocephalans?

Done.

REVIEWERS' COMMENTS:

Reviewer #1 (Remarks to the Author):

I am happy that the authors have addressed all queries.

Reviewer #2 (Remarks to the Author):

The points raised in my review have now all been dealt with satisfactorily in the revised MS, so I am happy to recommend the MS for publication.

Reviewer #3 (Remarks to the Author):

I consider that my comments have been included in the new version of the manuscript or answered by the authors in the rebutal letter.
So the article can be accepted now
